# Protocol for a four parallel-arm, single-blind, cluster-randomised trial to assess the effectiveness of three types of dual active ingredient treated nets compared to pyrethroid-only long-lasting insecticidal nets to prevent malaria transmitted by pyrethroid insecticide-resistant vector mosquitoes in Tanzania

**DOI:** 10.1136/bmjopen-2020-046664

**Published:** 2021-03-08

**Authors:** Jacklin F. Mosha, Manisha A. Kulkarni, Louisa A. Messenger, Mark Rowland, Nancy Matowo, Catherine Pitt, Eliud Lukole, Monica Taljaard, Charles Thickstun, Alphaxard Manjurano, Franklin W. Mosha, Immo Kleinschmidt, Natacha Protopopoff

**Affiliations:** 1 Parasitology, National Institute for Medical Research Mwanza Research Centre, Mwanza, United Republic of Tanzania; 2 Epidemiology & Public Health, University of Ottawa Faculty of Medicine, Ottawa, Ontario, Canada; 3 Disease Control, London School of Hygiene & Tropical Medicine, London, UK; 4 Global Health and Development, London School of Hygiene & Tropical Medicine, London, UK; 5 Clinical Epidemiology Program, Ottawa Hospital Research Institute, Ottawa, Ontario, Canada; 6 Parasitology, Kilimanjaro Christian Medical University College, Moshi, United Republic of Tanzania; 7 Infectious Disease Epidemiology, London School of Hygiene & Tropical Medicine, London, UK

**Keywords:** parasitology, infectious diseases, infection control, epidemiology, health economics, public health

## Abstract

**Introduction:**

The massive scale-up of long-lasting insecticidal nets (LLINs) has led to major reductions in malaria burden in many sub-Saharan African countries. This progress is threatened by widespread insecticide resistance among malaria vectors. This cluster-randomised controlled trial (c-RCT) compares three of the most promising dual active ingredients LLINs (dual-AI LLINs), which incorporate mixtures of insecticides or insecticide synergists to standard LLINs in an area of pyrethroid insecticide resistance.

**Methods:**

A four-arm, single-blinded, c-RCT will evaluate the effectiveness of three types of dual-AI LLINs (1) Royal Guard, combining two insecticides, pyriproxyfen and the pyrethroid alpha-cypermethrin; (2) Interceptor G2, combining chlorfenapyr and alpha-cypermethrin; (3) Olyset Plus, an LLIN combining a synergist, piperonyl butoxide and the pyrethroid permethrin, compared with; (4) Interceptor LN, a standard LLIN containing the pyrethroid alpha-cypermethrin as the sole AI. The primary outcomes are malaria infection prevalence in children aged 6 months–14 years and entomological inoculation rate (EIR), as a standard measure of malaria transmission at 24 months postintervention and cost-effectiveness.

**Ethics and dissemination:**

Ethical approval was received from the institutional review boards of the Tanzanian National Institute for Medical Research, Kilimanjaro Christian Medical University College, London School of Hygiene and Tropical Medicine, and University of Ottawa. Study findings will be actively disseminated via reports and presentations to stakeholders, local community leaders, and relevant national and international policy makers as well as through conferences, and peer-reviewed publications.

**Trial registration number:**

NCT03554616.

**Strengths and limitations of this study**

- This study is the first randomised controlled trial (RCT) to evaluate and compare the effectiveness of the next generation of dual-treated long-lasting insecticidal nets (LLINs), Royal Guard (a pyrethoid-pyriproxifen LLIN) and Interceptor G2 (a pyrethroid-chlorfenapyr LLIN) against standard LLIN to prevent malaria infection prevalence and incidence in an area of pyrethroid insecticide resistance.
- Results of this study will be presented to the WHO for policy recommendations and depending on the study outcomes has the potential to shape malaria vector control strategies across sub-Saharan Africa for the next decade.
- This is the second RCT to evaluate the new class of pyrethroid-piperonyl butoxide LLINs (Olyset Plus) in the Great Lakes Zone of Tanzania, expanding the evidence basis for deployment of this intervention class in areas of pyrethroid resistance.
- The trialling of the three main categories of dual active ingredients LLIN against the standard pyrethroid LLIN in the same human cultural community against the same vector complex should offer the best opportunity to unravel relative effectiveness on malaria and effect on selection of insecticide resistance.
- Some limitations include the size of the cluster buffer areas, which might not prevent all contamination between intervention arms, and the use of malaria rapid diagnostic tests (mRDT) to assess malaria infections rather than double-read blood slides, which may be more sensitive.

## Introduction

Long-lasting insecticidal nets (LLINs) are the primary method of malaria control in sub-Saharan Africa. The WHO estimates that over 50% of the population of sub-Saharan Africa now sleep under LLINs.^1^ Together with improved diagnosis and treatment, LLINs have helped reduce malaria incidence by 42% and mortality by 66% in Africa in the last 15 years.^1^ Pyrethroids are the only class of insecticide used routinely on LLINs, so the rapid spread of pyrethroid resistance across vector populations threatens to reverse the successes achieved so far, and may be a factor contributing to the current stagnation in malaria disease burden.^2^

Several studies have demonstrated that LLINs are becoming less effective at killing mosquitoes in areas of moderate to high pyrethroid resistance compared with those with susceptible vector populations.^3–8^ The search for new insecticides suitable for LLIN treatment is vital to sustain effective malaria vector control.

During the last decade, the WHO has encouraged manufacturers to develop new types of bed nets to control resistant mosquitoes. The chemical industry responded initially by developing a dual active ingredients LLIN (dual-AI LLIN), combining a pyrethroid insecticide with the synergist piperonyl butoxide (PBO), which inhibits cytochrome P450 oxidases (CYP) responsible for pyrethroid resistance. Pyrethroid-PBO LLINs (py-PBO LLINs) have been available since 2009,^9^ but had only been recommended by WHO for limited deployment until^10^ a recent cluster-randomised controlled trial (RCT) demonstrated a 44% reduction in malaria infection prevalence in children in the py-PBO LLIN arm (Olyset Plus) compared with the standard pyrethroid LLIN (s-LLIN) after 2 years.^8^ In 2017, based on this study, the WHO recognised the public health value of this new class of LLIN and recommended their scale up in area with pyrethroid resistance.^11^ A second large-scale RCT in Uganda confirmed the superior protection of py-PBO LLIN against malaria compared with s-LLINs.^12^

Other manufacturers have responded to the WHO call by producing a different kind of dual-AI LLIN that incorporates a mixture of insecticides from different insecticide classes. Mixtures of two insecticides on the same LLIN with differing modes of action have the potential to delay the evolution of resistance and extend the lifespan of both active ingredients on the LLIN. The two latest products are a pyrethroid-pyriproxyfen LLIN (py-PPF LLIN: Olyset Duo and Royal Guard) and a pyrethroid-chlorfenapyr LLIN (py-CFP LLIN: Interceptor G2). Both types of product have shown superior efficacy compared with s-LLINs in small-scale experimental hut trials.^13–20^ The Py-PPF LLINs have demonstrated enhanced efficacy vector oviposition suppression and up to 95% reduction in vector reproductive rate in Benin.^14 21^ In addition, Py-PPF LLIN Olyset Duo, when compared with s-LLINs in an RCT in Burkina Faso, had a significantly greater impact on clinical malaria.^22^ The py-CFP LLIN, when compared with s-LLINs, has shown enhanced efficacy through higher killing against resistant mosquito species in experimental hut trials, producing mortalities with *Anopheles gambiae* s.l. of 71% versus 20% in Benin^20^ and 78% versus 17% in Burkina Faso,^18^ and 71% versus 45% with *Anopheles arabiensis*^23^ and 70% versus 37% with *Anopheles funestus* s.l. in Tanzania.^24^ Neither of these new AIs, pyriproxifen or chlorfenapyr, are related to one another nor show cross resistance. However, to receive a WHO public health recommendation, these ‘next-generation’ LLINs still need to be evaluated in two RCT^25^ to demonstrate their effectiveness against malaria in human populations in areas characterised by different insecticide resistance intensities and major vector species.

In Tanzania, insecticide resistance has spread rapidly.^26–28^ In regions where resistance is particularly strong, such as North-West Tanzania, the prevalence of malaria infection remains high (40% in children under 5 years old), despite universal coverage of s-LLINs.^29^ This finding echoes reports from Uganda, where no reduction in malaria incidence was observed after the distribution of s-LLINs.^30^ As operational failure of standard nets is occurring more frequently in areas with pyrethroid resistance, including the Great Lakes Zone,^30^ the newly developed ‘next-generation’ LLINs now require urgent comparative evaluation.

Here, we describe the study design and methodology of an RCT, assessing the effectiveness and cost-effectiveness of three novel vector control interventions (dual-AI LLINs incorporating mixtures of insecticide classes or insecticide synergists), compared with the standard best practice of pyrethroid-only LLINs, to prevent malaria in an area of pyrethroid resistance in Tanzania. Each putative new class needs to show high effectiveness versus the s-LLIN. None should select for stronger resistance to pyrethroid. Ideally, none should select for resistance to the other new classes of dual-AI LLIN being tested. A four-arm trial is a highly efficient design, should demonstrate the relative effectiveness of each against malaria, and the merits of each product against the same fauna of vector species and human cultural group. The trial should provide insight into future rotational strategies of deployment, their potential to manage insecticide resistance while controlling malaria.

The durability and bio-efficacy of the dual-AI LLIN are also being evaluated as per WHO guidelines^31^ and will be presented in a separate protocol, published elsewhere. The study protocol is reported in line with the Standard Protocol Items: Recommendations for Interventional Trials 2013 statement.^32^

## Study objectives

The primary clinical and entomological objectives are to assess the effectiveness of py-PPF-LLINs, py-CFP-LLINs and py-PBO-LLINs compared with s-LLINs:

- On malaria infection prevalence in children from 6 months–14 years over 2 years postintervention.
- On the entomological inoculation rate (EIR) of malaria vectors collected indoors over 2 years post intervention.

The primary economic objective is to assess the cost-effectiveness of py-PPF-LNs, py-CFP-LNs, py-PBO-LNs and s-LLINs relative to one another.

**Figure 1:**
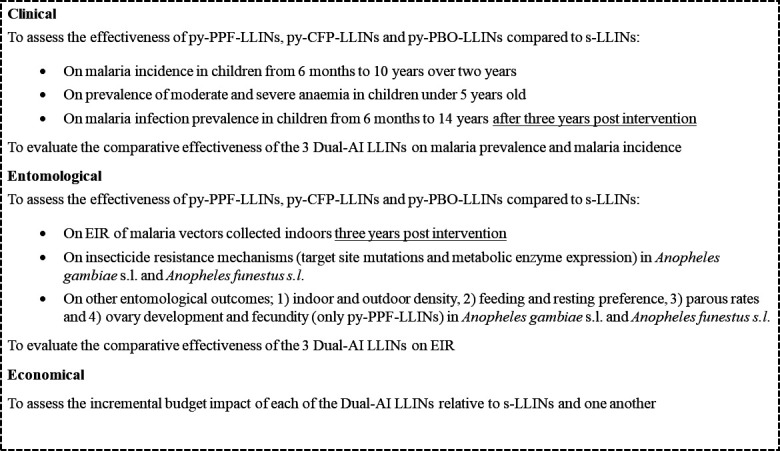
Secondary objectives. Dual-AI-LLINs, dual active ingredients LLINs; EIR, entomological inoculation rate; LLINs, long-lasting insecticidal nets; py-PBO-LLINs, pyrethroid-piperonyl butoxide LLINs; py-PPF-LLINs, pyrethroid-pyriproxyfen LLINs; s-LLINs, standard pyrethroid LLINs

All secondary objectives are detailed in figure 1.

## Methods and analysis

### Study setting

Five potential study sites in Tanzania’s Victoria lake zone were evaluated based on four criteria: report of a minimum of 30% malaria infection prevalence in total population, *A. gambiae* sensu stricto (s.s.) or *A. funestus* s.s. as the main vectors, insecticide pyrethroid resistance in standard WHO bioassays (<50% mortality), and no indoor residual spraying (IRS) planned for the next 3 years. Data from published and unpublished sources (including the National Malaria Control Programme, NMCP; and the President’s Malaria Initiative, PMI) were examined and complemented with data collection in November 2017, as appropriate. Only the district of Misungwi (2°51′00.0″S, 33°04′60.0″E), on the southern border of Lake Victoria in Tanzania met all the criteria.

Misungwi covers an area of 2122 km^2^ and includes 27 wards, 78 villages and a population of 351 607 people.^33^ Average altitude in the study area is 1150 m. The annual rainfall ranges from 0.5 mm to 58.8 mm, split in two rainy seasons (October to December and March to May) and interrupted by a distinct long dry season (June to August/September) and a second short dry season in late December to February.^34^

Misungwi has moderate to high malaria transmission. In a study conducted in 2010, prevalence was 52% across all age groups.^35^ During the preliminary assessment in May 2018, presence of all main Tanzanian malaria vectors, *A. gambiae* s.s., *A. arabiensis* and *A. funestus* s.s., were found in the area (unpublished data). WHO insecticide resistance tests were also performed and 24-hour mortality in wild caught *A. gambiae* s.s. exposed to permethrin and deltamethrin was 7% and 19%, respectively. There was also evidence of pyrethroid resistance in *A. funestus* s.s. (mortality 50%) and *A. arabiensis* (mortality 65.3%). Pyrethroid insecticide resistance has also been observed in the adjacent district of Magu and other lake zone regions, such as Geita and Kagera.^28^

The main vector control interventions in Misungwi are universal coverage of LLINs and IRS. The last distribution of LLINs and IRS campaign using Actellic 300CS were carried out in Misungwi district in 2015. In 2017, larviciding was done in some parts of the district.^36^ The district is also following national malaria control measures such as intermittent preventive treatment of malaria in pregnant women.

### Study design

**Figure 2:**
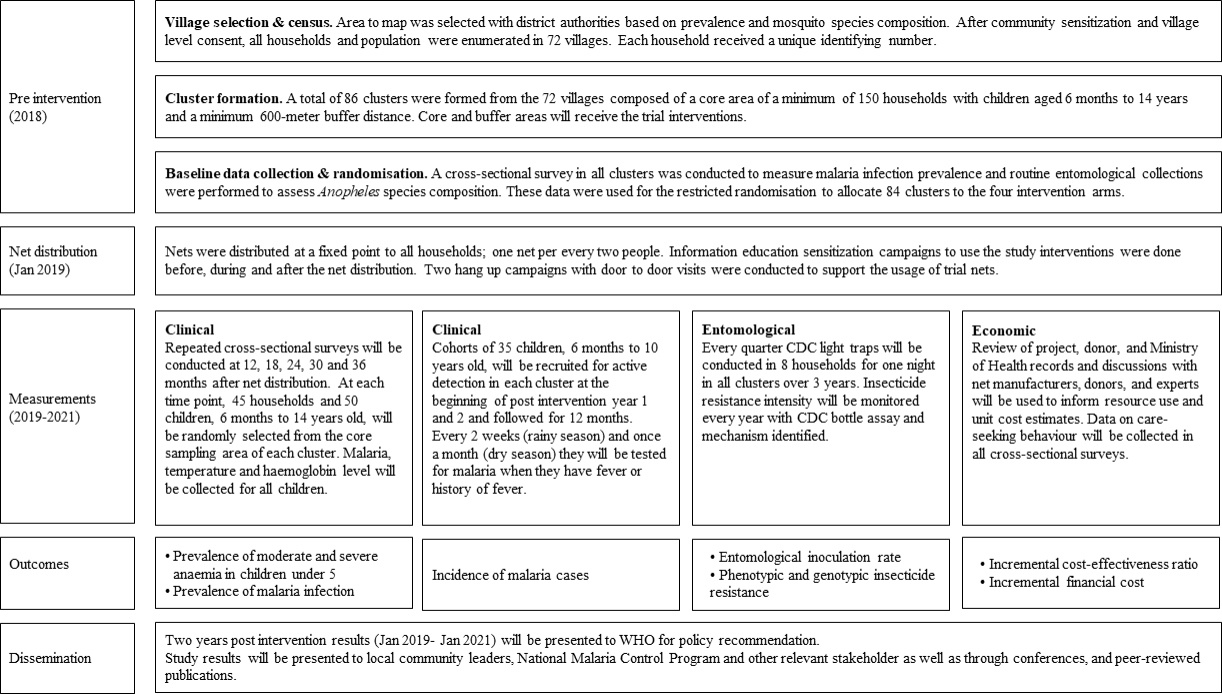
Trial study design. CDC, Centers for Disease Control and Prevention.

An overview of the trial design is given in figure 2. The design is a four parallel-arm, single-blind, superiority cluster randomised trial with village hamlet as the unit of randomisation and repeated cross-sectional survey. The four study arms are:

- Mixture py-PPF-LLIN: Royal Guard (intervention 1).
- Mixture py-CFP-LLIN: Interceptor G2 (intervention 2).
- Py-PBO-LLIN: Olyset Plus (intervention 3).
- S-LLIN: Interceptor (control arm).

The inhabitants of each cluster are blinded to the type of nets they received, as are the field staff who will collect entomological and clinical data. Nets of each type are similar in appearance apart from a colour-coded loop and a unique identifying number. Only the principal investigator and data manager know which code represents each intervention.

### Mapping and cluster formation

Every building of 72 villages comprising 453 hamlets from 17 wards was mapped using a global positioning system handheld unit (Garmin Legend e-trex) and ExpertGPS V.3.8 (TopoGrafix) software. A short Open Data Kit (ODK) programmed questionnaire, including name of the head of the household, number of people living in the house and number of children in each age group (6 months–59 months, 5 years–10 years, 11 years –14 years) was recorded for each mapped household.

Wards to map were selected based on both malaria incidence data collected from all health facilities in Misungwi, and *Anopheles* species composition (only areas with the main malaria vectors *A. funestus* s.s. and *A. gambiae* s.s were considered), assessed during pilot mosquito monitoring from all villages.

**Figure 3:**
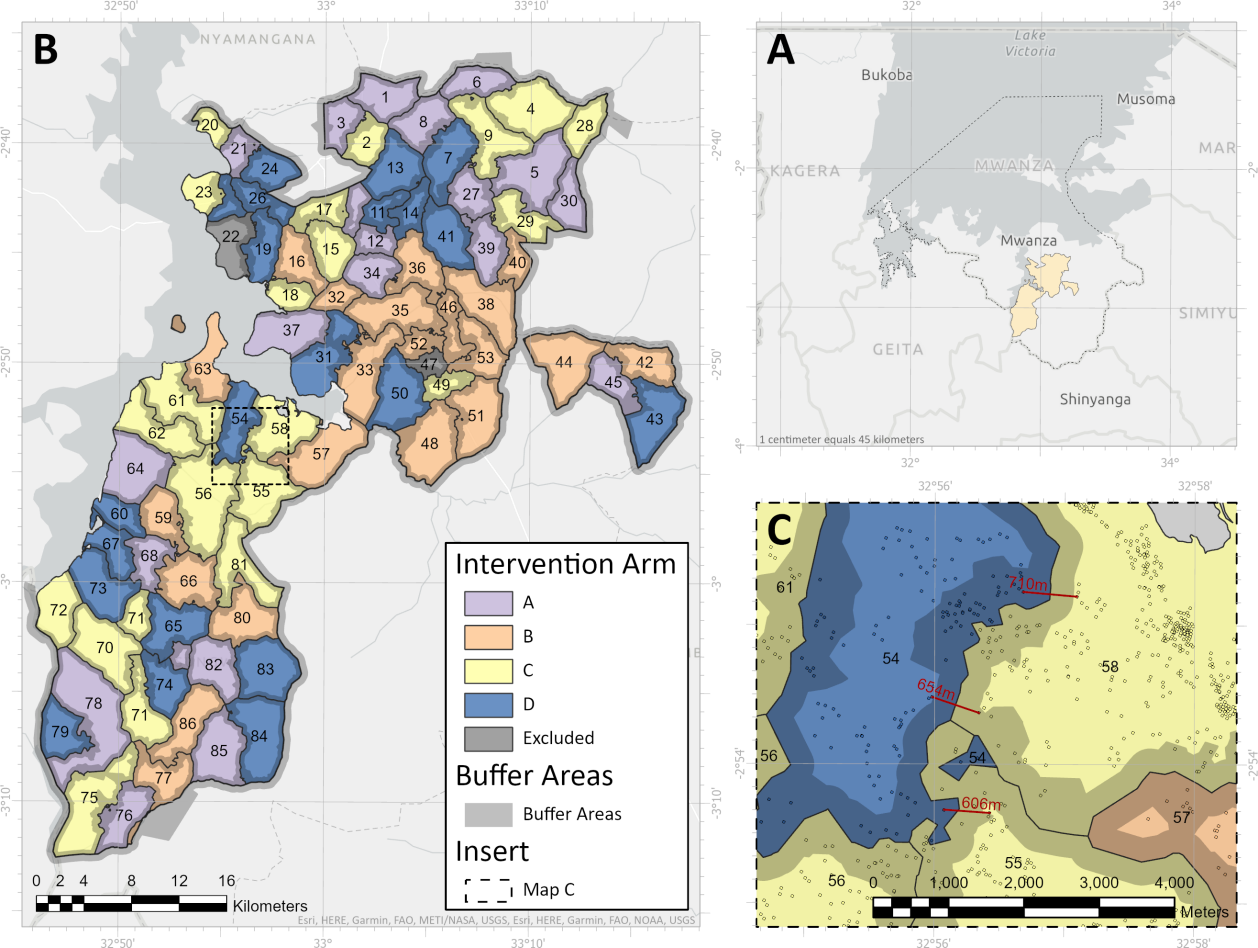
Study area. Map showing Misungwi study area in Mwanza region, North Tanzania (A), the 85 study clusters identified with core and buffer area and intervention allocation (B). (C) Closer map on the minimum 600 000 m area between houses in adjacent clusters.

As in previous studies,^8^ clusters were designed with core and buffer areas to reduce the likelihood of spill-over of intervention effects from one cluster to another. Nets will be distributed to all households in a given cluster (ie, core and buffer areas), but monitoring of outcomes will be restricted to households situated in the cluster core. A total of 86 clusters were formed from the 72 villages (figure 3) using the spatial analyst toolbox in ArcGIS (ESRI, Redlands, the USA) based on the following criteria: no subdivision of village hamlets, minimum of 150 households with children aged 6 months–14 years in the core area, and a minimum 600-metre buffer distance. The number of households in each cluster varied from 172 to 2390 (urban area of Misungwi) with an average of 492 households. A 600-metre buffer (ie, 600 m between the margins of core areas for any two adjacent clusters) was allocated thereby retaining 73.6% (31 125/42 314) of the households in the core area for sampling and data collection, with 134–828 core area households per cluster (figure 3C). Only two clusters did not meet the criteria of 150 households in the core area, and were therefore excluded.

### Randomisation

After the completion of the baseline survey, covariate constrained randomisation was used to allocate the 84 clusters across the four study arms. Covariate constrained allocation ensures that the arms are balanced overall by excluding allocations where predetermined factors are not balanced within set margins.^37^ The following factors were constrained: baseline (preintervention) malaria infection prevalence in children aged 0.5–14 years, previous LLIN usage, socioeconomic status (SES), population size, suitable conditions for *A. gambiae* s.s. and for *A. funestus* s.l. Suitability for each *Anopheles* species was determined using an ecological niche modelling approach (online supplemental file 1) as it was not possible to assess the species composition in all clusters before the randomisation. From among all possible allocations meeting the balancing constraints, one allocation was selected at random. The randomisation was performed by independent statistician.

10.1136/bmjopen-2020-046664.supp1 Supplementary data

### Interventions

In all four trial arms, net distribution was carried out in an identical manner by the Tanzania Communication and Development Center, a Tanzanian not-for-profit organisation, and supervised by the NMCP to follow national net distribution campaign guidelines.^38^ All households, enumerated in the core and buffer area, were allocated one net for every two people as recommended by the Tanzania NMCP.^38^ Information, education and communication (IEC/SBCC) activities were conducted by a Tanzanian NGO, Tulonge Afia, before, during and after the net distribution to increase usage in the study area and supported by the US President Malaria Initiative. Householders were not asked to return their old net but to use the new net provided. To maximise effective LLIN coverage, two door-to-door hang-up campaigns were done after 2 weeks and again 3 months after distribution.

All nets distributed were blue and rectangular (180 cm length×160 cm wide×180 cm high). They differed by study arm as described in the following sections.

#### Control

Interceptor LN (BASF Corporation, Germany) is a pyrethroid-only LLIN with alpha-cypermethrin at a target dose of 200 mg/m^2^, coated onto polyester filaments. Pyrethroids are neurotoxic insecticides, which target the nervous system of insects. Interceptor LN was chosen as a direct comparison to Interceptor G2 LN and Royal Guard LN, as they are all impregnated with the same pyrethroid (alpha-cypermethrin); some insecticide resistance mechanisms involving CYPs are specific to type I (permethrin) or type II (alpha-cypermethrin) pyrethroids.

#### Intervention 1

Royal Guard LN (Disease Control Technologies) is a mixture LLIN made of polyethylene incorporating 225 mg/m^2^ PPF and 216 mg/m^2^ alpha-cypermethrin. PPF is known to disrupt female mosquito reproduction and fertility of eggs, stopping the production of the next generation of mosquitoes.^39^ PPF may be transferred by auto-dissemination; the transfer of small but toxic amounts of this highly potent insecticide on the tarsi (feet) of female mosquitoes to breeding sites where it can also act as a larvicide.^40^

#### Intervention 2

Interceptor G2 LN is a mixture LLIN made of polyester coated with a wash-resistant formulation of 200 mg/m^2^ chlorfenapyr and 100 mg/m^2^ alpha-cypermethrin. Chlorfenapyr disrupts cellular respiration and oxidative phosphorylation in mitochondria, and due to this unique mode of action is toxic against mosquitoes that are resistant to standard neurotoxic insecticides like pyrethroids.^41^

#### Intervention 3

Olyset Plus LN (Sumitomo Chemical) is a mixture LLIN combining PBO (400 mg/m^2^) and the repellent pyrethroid permethrin (800 mg/m^2^) incorporated into polyethylene fibres. PBO is a chemical synergist which acts by inhibiting mixed function oxidases, preventing detoxification of the pyrethroid insecticide. Two RCTs demonstrated that Olyset Plus LLINs were more effective than Olyset Net LN (the standard of care in Tanzania), in areas with pyrethroid resistance.^8 12^

### Study outcomes

#### Primary outcomes

The primary outcomes (table 1) will be:

**Table 1:** Trial outcome measurements

Outcome	Measurement	Collection	Frequency
**Clinical outcomes**			
Malaria infection prevalence	Rapid diagnostic test	Cross-sectional survey	Baseline, 12, 18, 24, 30 and 36 months postintervention
Anaemia	Haematocrit	Cross-sectional survey	Baseline, 12, 18, 24, 30 and 36 months postintervention
Temperature	1. Digital ear thermometer all children 2. Temperature and history of fever	1. Cross-sectional survey 2. Cohort follow-up	1. Baseline, 12, 18, 24, 30 and 36 months postintervention 2. Every month
Malaria case	Rapid diagnostic test taken when fever ≥37.5°C and or history of fever for the past 48 hours	Cohort follow-up	Every 2 weeks during high transmission season (October to July) and every month during dry season (August to September)
**Measurement of entomological outcomes**			
Indoor *Anopheles* density	CDC light traps	Entomology surveillance	8 houses per cluster every 3 months in all clusters for 3 years
Outdoor biting	Adapted Furvela tent trap^55^	Sentinel site	Two times a year in 2–3 sentinel site per arm
Mosquito sporozoite rate	CSP-ELISA to estimate EIR^46^	Entomology surveillance	Subsample (30%) of the mosquito collected in CDC light trap
*Anopheles* species identification	*1. A. funestus* s.l. complex: conventional PCR or multiplex real time PCR^49^ *2. A. gambiae* s.l. complex: TaqMan real time PCR^48^	Entomology surveillance and Sentinel site and resistance test	Subsample of mosquitoes collected
Insecticide resistance frequency and intensity	WHO cylinder assay^52^ CDC bottle bioassays^51^	Collection of adult *Anopheles* resting indoors	Once a year in a subsample of clusters
Frequency of Vgsc mutation	TaqMan PCR^50^	Entomology surveillance	Subsample of mosquitoes collected in light trap
Insecticide resistance mechanisms	Multiplex TaqMan reverse-transcription quantitative PCR (RT-qPCR) will be used to monitor expression of CYPs and other metabolic enzymes known to be over-expressed in resistant *A. gambiae* s.s. and *A. funestus* s.s. populations from previous studies in Tanzania^54^	Collection of adult *Anopheles* resting indoors, previously phenotyped in resistance bioassays	At baseline and at each postintervention year in a subsample of clusters

CDC, Centers for Disease Control and Prevention; CSP, circumsporozoite protein; EIR, entomological inoculation rate.

- Malaria infection (by mRDT) in children aged 6 months–14 years old at 24 months postintervention.
- EIR as a measure for malaria transmission in the primary vector species.
- Cost-effectiveness of each of the four net types relative to one another.

#### Secondary outcomes

- Incidence of malaria cases in children aged 6 months–10 years (measured over 24 months follow-up).
- Moderate and severe anaemia in children under 5 years old (<8 g/dL) at 12, 18, 24, 30 and 36 months.
- Malaria infection (by mRDT) in children aged 6 months–14 years at 12, 18, 30 and 36 months.
- Changes in frequency and intensity of phenotypic and genotypic resistance to pyrethroids (alpha-cypermethrin and permethrin), PBO, CFP and PPF.
- Other entomological outcomes: changes in mosquito resting behaviour indoors and outdoors, species composition and density ratio, host feeding, mosquito ovary development and fecundity.
- Incremental financial cost to the provider of each of the four net types relative to one another.

### Data collection

#### Intervention coverage data

LLIN coverage will be evaluated 6 months after distribution and during each cross-sectional survey. Three indicators will be used^42^: (1) ‘proportion of households with at least one LLIN for every two people (ownership)’, (2) ‘proportion of household with enough LLINs to sleep under (access)’ and ‘proportion of households declaring using an LLIN (study or not) last night (usage)’.

#### Clinical data

To determine infection prevalence, repeated cross-sectional surveys will be conducted at 12, 18, 24, 30 and 36 months after net distribution (table 1). At each time point, 45 households will be randomly selected from the core sampling area of each cluster, using the census list generated during baseline enumeration. In each house a maximum of two children, between 6 months and 14 years old, will be selected. Accounting for households that are closed, refusing informed consent, or do not have children of the required age at the time of the survey, this sampling strategy is expected to yield an average of 30 enrolled houses with 50 children per cluster based on previous studies.^8 43^ A total of 4200 children are thus expected to be surveyed at each time point.

Inclusion criteria are households with at least one child between 6 months and 14 years old who permanently resides in the selected household and an adult caregiver who can provide written consent. Exclusion criteria include dwellings not found or vacant during the survey, no adult caregiver capable of giving informed consent, or eligible children are severely ill.

Information on sex, age distribution, educational status and occupation, SES, house structure, vector control measures used, past malaria cases, net coverage and care seeking behaviour will be collected. Fever or history of fever in the past 48 hours will be recorded for every child selected. Temperatures will be taken, and each child tested for malaria using mRDTs (CareStart RDTs; HRP2, (pf), DiaSys, Wokingham, UK) and haemoglobin levels measured (HemoCue Hb 201+ (Aktiebolaget Leo Diagnostics, USA)). When the mRDT is positive, free treatment for malaria will be provided with artemether-lumefantrine (artemisinin-based combination therapy; ACT), as per the national guidelines. Children with severe malaria or any other diseases that cannot be treated by the team will be referred to the nearest health facility.

To assess malaria case incidence, 35 children per cluster, aged 6 months–10 years old, will be randomly selected and followed up every 2 weeks during the high transmission season (October to July) and every month during the low transmission season (August to September), over a 12 months period (table 1). To reduce attrition, another cohort of children will be selected at the beginning of the second year and will also be followed for 12 months. All cohort children will be cleared of malaria infection at the beginning of the first and second year by ACT treatment. Cohort children will then be checked 2 weeks later by mRDT and microscopy (if the mRDT is positive) to confirm whether they have been cleared. During each bi-weekly or monthly visit, children with fever ≥37.5°C and/or history of fever for the past 48 hours will be tested for the presence of malaria parasites (case incidence) by mRDT. For those with positive mRDT results, a blood slide will also be taken to confirm malaria positivity and they will also be treated, as previously described. Children will be encouraged to visit a health facility in case they have fever, or get sick at any time between visits. Each child will be provided with a personal medical book where malaria episodes will be recorded during study visits or whenever they attend a health facility and also will be provided with medical insurance. During cohort visits, a questionnaire will also be administered to inquire about net usage the night before the visit, any adverse events encountered and travel history within the past 2 weeks.

#### Entomological data

Cross-sectional entomological surveys will be carried out in 84 study clusters to monitor the indoor mosquito population density (table 1). Each cluster will be visited once every quarter; each month, seven clusters from each study arm will be selected. Indoor mosquito densities will be monitored using Centers for Disease Control and Prevention (CDC) Miniature light traps (John W Hock Company, USA) in eight randomly selected households in the core area of each cluster. CDC light trap will be installed at the feet of one bed and existing net substituted with a project standard LLIN, and replaced the following day. For each of the selected houses, a short questionnaire will be administered to collect information about the number of inhabitants, type of house construction materials, presence of animals, coverage and usage of nets, and other malaria prevention measures used by household members. Sampled mosquitoes will be identified morphologically following the identification key by Gillies MT and Coetzee M. A supplement to the Anophelinae of Africa south of the Sahara (Afrotropical region), 1987.^44^ Parity rates will be estimated in a subset of live *Anopheles* mosquitoes through dissection.^45^ About 10 *A. gambiae* s.l and 10 *A. funestus* s.l will be randomly picked per household per collection night and preserved for laboratory analysis. This sample of mosquito specimens will be screened for *Plasmodium falciparum* circumsporozoite protein (Pf-CSP) by ELISA.^46^ The CSP-ELISA positive samples will be reanalysed by heating the ELISA lysates to remove any false positives.^47^

PCR TaqMan assays will be used to discriminate members of *A. gambiae* species complex,^48^ and members of the *A. funestus* group^49^ on a subsample of *Anopheles* collected. A sample of *A. gambiae* s.s. and *A. arabiensis* will be genotyped for the L1014F-*kdr* and L1014S-*kdr* mutations, associated with pyrethroid and DDT resistance, using TaqMan PCR assays, following the protocol by Bass *et al.*^50^

Phenotypic resistance and resistance levels to alpha-cypermethrin, permethrin, PPF and chlorfenapyr, will be characterised at baseline and yearly postintervention, in all four study arms, using WHO cylinder and modified CDC bottle bioassays.^51 52^ The PBO synergist effect on wild female *A. gambiae* s.l. and *A. funestus* s.l. will be assessed using pre-exposure to PBO followed by permethrin resistance intensity assays. All knockdown/dead mosquitoes at 60 min and surviving 72 hours postexposure to insecticides will be stored individually in RNA later and preserved at −20°C for gene expression analysis. RNA will be extracted from pools of *A. gambiae* s.s. or *A. funestus* s.s and cDNA synthesised, according to standard procedures. Relative expression of CYPs and other metabolic enzymes, previously identified as being over-expressed in resistant *A. gambiae* s.s. and *A. funestus* s.s. populations in Tanzania,^53^ will be measured using multiplex TaqMan RT-qPCR assays.^54^

Malaria vector abundance, species composition, feeding and resting behaviours (including biting time, host preference), and contribution to outdoor malaria transmission will be assessed at baseline and after implementation of the interventions to assess changes over time in 10 clusters per treatment arm. CDC light trap and adapted Furvela tent traps^55^ will be used for indoor and outdoor collection of free flying mosquitoes. Resting *Anopheles* will be collected using CDC prokopack aspirators. Subsamples of *Anopheles* will be subjected to the same laboratory tests than routine collection. Additional information about mosquito collection methods is available in online supplemental file 1.

#### Economic data

Data on resource use and unit costs will be collected from primary and secondary sources, including discussions with net manufacturers, donors and experts; review of project, donor and Ministry of Health records; trial survey data on care seeking behaviour; published literature; and WHO-CHOICE unit cost estimates.^56^ Data on health outcomes will include primary trial data on malaria incidence and anaemia, as well as secondary data on the age structure of malaria incidence and life expectancy.^57 58^

### Sample size and power consideration

#### Malaria prevalence cross-sectional survey

The sample size was calculated using the method of Hayes and Bennett, taking into account the cluster-randomised design.^59^ For the primary outcome prevalence of infection at 24 months, we assumed a malaria prevalence in the reference arm of 40%, an average of 50 individuals per cluster and a coefficient of variation of 21%, based on recent surveys in a similar area.^60^ To achieve 80% power to detect a prevalence ratio of 0.72, that is, a 28% lower prevalence in at least one of the intervention arms versus the reference at Bonferroni-corrected significance level of 1.67%, we require 21 clusters per arm. This calculation is conservative as it does not account for the repeated malaria prevalence measures; thus, we anticipate being able to detect even smaller differences.

#### Incidence of malaria infection in children cohort

Sample size calculations for the secondary outcome (cumulative malaria incidence) were based on the method of Hayes and Bennett.^59^ Based on a previous study (Jacklin Mosha, personal communication), it was assumed that the mean number of malaria episodes per child per year in the reference arm was 0.85 (monthly event rate of 0.071) with a between-cluster coefficient of variation of 21%. With a cohort of 35 children per cluster (21 clusters per arm), and accounting for attrition of 30% over 24 months, we would achieve 80% power to detect a 23.6% relative reduction in malaria cases per child per year (risk ratio 0.764) between at least one intervention arm relative to the reference arm, using a two-sided Bonferroni-corrected significance level of 1.67%. If the incidence in the reference arm is lower, at 0.5 episodes/year, we will still have 80% power to detect a 26.1% relative reduction (relative risk 0.739).

#### Entomological survey for EIR estimation

Sample size calculations for the entomological survey were based on the method of Hayes and Bennett.^59^ It was assumed that the mean EIR (number of infectious bites per household per month) in the reference arm was 1.76 with a between-cluster coefficient of variation of 40%, based on a previous study.^8^ With a sample of eight households per cluster and sampling each cluster every quarter we will have collections for 32 house-nights per cluster per year. With 21 clusters per arm the study would achieve 80% power to detect a 36% relative reduction in monthly EIR (relative risk 0.64) between at least one of the intervention arms relative to the reference using a Bonferroni-corrected significance level of 1.67%. Changes in insecticide resistance frequency and resistance management potential will be assessed separately from light trap and household resting collections.

### Data management

Clinical and entomological measurements in the cohort study and the cross-sectional surveys will be captured on electronic forms using tablets/smartphones installed with ODK and uploaded daily onto the server at London School Hygiene and Tropical Medicine (LSHTM). Other data and clinical measurements during the cross-sectional survey, recorded on paper, will be double-entered into an access database independently by two data clerks. Laboratory data outputs will be available directly from the analyser (eg, ELISA data) and imported into a database. All databases will maintain an audit trail with time-date stamps of data entry and all changes that are made to the data. Anonymised study numbers will be used as unique participant identifiers.

### Data analysis

#### Primary outcomes

##### Prevalence of malaria infection and EIR

The primary analysis will be conducted using the intention to treat (ITT) approach. Secondary per-protocol analyses will also be conducted. The primary outcome, measured at 12, 18, 24, 30 and 36 months after net distribution, will be analysed using mixed effects logistic regression. The unit of analysis will be the individual. The model will include fixed effects for time, study arm, and time by study arm, and will adjust for the baseline prevalence in each community as a covariate, as well as the covariates used in the covariate-constrained allocation procedure. The model will account for within-period and between-period intra-cluster correlations.^61^

Prevalence of malaria infection in each dual-AI LLIN treatment arm will be compared with the prevalence of infection in the reference arm using least square mean differences to assess whether the new LLINs are superior. The primary comparison will occur at the end of the 24 months postintervention period. To adjust for the increased risk of a type I error due to multiple pairwise comparisons, the level of significance will be adjusted using the Bonferroni method. Least square mean differences between each intervention arm versus control will be calculated at each time, together with 98.23% CIs (reflecting adjustment of usual 95% CI to account for multiple comparisons). Secondary analyses will include all possible pairwise comparisons of dual-AI LLIN arms.^62^ Subgroup analyses will be performed to investigate the impacts of interventions according to different individual (ie, age, sex), household (ie, wealth, distance to health facilities) and cluster-level (ie, vector resistance intensity, vector species composition) characteristics.

EIR will be estimated as the mean number of sporozoite infected mosquitoes per house per night (by species and overall) and weighted to account for proportion of mosquitoes processed for sporozoites. Differences in *Anopheles* density and EIR between the different arms will be estimated using random effects negative binomial regression taking into account the intracluster correlation. Random effects logistic regression will be used to compare sporozoite rate between study arms.

##### Economic evaluation

Following relevant guidelines,^63 64^ we will combine primary trial data with secondary data in a decision analytic model using a decision tree. We will adopt a societal perspective, presenting costs both combined and disaggregated by payer, and model effects and costs over a lifetime horizon. In the main analysis, we will assume that nets are distributed in mass campaigns every 3 years, reflecting both what was done in the trial and standard practice in malaria control.

Effects will be presented as disability-adjusted life years (DALYs) discounted at 3% and with no age weighting, calculated as the sum of years of life with disability from malaria-related illness (uncomplicated and severe malaria cases and anaemia) and years of life lost from malaria deaths.^63^ The number of cases associated with each intervention will be modelled as the product of the incidence in the reference arm, the incidence rate ratio of the relevant arm to the reference arm, and a standardised population size. Incidence in children aged 6 months–10 years will reflect ITT trial data, while incidence in other age groups, which comprise a small share of overall cases and deaths, will be estimated as a function of the incidence in children based on publicly available modelling.^58 65^ DALYs will be calculated as the product of the estimated number of malaria cases (as earlier), the case fatality rate, and the remaining life expectancy at age of death. Rates of progression to severe disease and to death will be estimated to reflect real-world outcomes based on secondary data. In the main analysis (based on trial data at 24 months), possible bounds for incidence rate ratios over months 25–36 will be estimated based on annual rate ratios over 0–12 and 13–24 months and assuming no relative effect compared with s-LLINs. In additional analyses, effects will also be presented as malaria cases and percentage point reduction in malaria prevalence.

We will plot the costs and effects of each intervention on the cost-effectiveness plane, identify the cost-effectiveness frontier and expansion path, and calculate incremental cost-effectiveness ratios between adjacent points on this path. Deterministic and probabilistic sensitivity analysis will explore the impact of uncertainty and heterogeneity on cost-effectiveness results. Cost-effectiveness acceptability curves^66^ will indicate the probability of each strategy being the most cost-effective choice at plausible cost-effectiveness thresholds.^67 68^ Affordability will be explored by comparing additional financial costs (net of cost savings) per person to relevant expenditure levels. Analyses will be conducted in Microsoft Excel with Visual Basic for Applications.

#### Secondary outcomes

##### Incidence

Cumulative incidence of malaria infection over the 24 months follow-up (accounting for repeat episodes in the same child but measured on separate cohorts at 12 and 24 months postintervention) will be analysed using mixed effects Poisson or negative binomial regression. The model will include fixed effects for time, study arm, and time by study arm, and will adjust for the covariates used in the covariate-constrained allocation procedure. The model will account for within-period and between-period intra-cluster correlations. Least square mean differences will be obtained from the model to compare each intervention versus the control. Follow-up within 14 days of a previous episode in the same child will be censored and will not be included in the analysis. An offset for duration of follow-up will be included in the model to account for attrition.

##### Prevalence of anaemia

Prevalence of moderate and severe anaemia in children under 5 at 12,18, 24, 30 and 36 months will be analysed using mixed effects logistic regression similar to the approach outlined for malaria prevalence.

##### Insecticide resistance monitoring

Bioassay data will be interpreted according to the updated WHO guidelines: mortality of ≥98% indicates susceptibility at the diagnostic dose, mortality of 90%–97% is suggestive of resistance, and mortality of less than 90% indicates resistance.^69^ For resistance intensity assays at 5X insecticide concentrations, mortality ≥98% indicates low intensity resistance and mortality <98% indicates moderate to high intensity resistance. For resistance intensity assays at 10X insecticide concentrations, mortality ≥98% indicates moderate intensity resistance and mortality <98% indicates high intensity resistance.

For metabolic gene assays, relative expression level and fold change of each target gene from resistant and susceptible field samples, relative to the susceptible laboratory strain (*A. gambiae* s.s Kisumu or *A. funestus* s.s FANG), will be calculated using the 2^−ΔΔCT^ method, incorporating PCR efficiency and normalised relative to the endogenous housekeeping control gene.^70^

### Patient and public involvement

There was no patient or public involvement in the design of this study. Communities will be involved in the implementation of the interventions and study activities through their leaders and community health representatives.

### Ethics approval

This protocol has been reviewed and approved by all institutional review boards, including: the Medical Research Coordinating Committee of the National Institute for Medical Research, LSHTM, Kilimanjaro Christian Medical University College and University of Ottawa.

This study will be conducted according to the Declaration of Helsinki and the International Guidelines for Ethical Review of Epidemiological Studies. All field and clinical staff as well as the principal investigator will receive training on good clinical and laboratory practice before data collection begins, and refresher training every year. For all data collection activities (epidemiological and entomological), written informed consent (online supplemental file 2) will be obtained from an adult guardian in the household. The consent form will be written in Swahili and indicate the purpose of the study, the procedures, risks and benefits, that participation is completely voluntary, and that they may withdraw at any time with impunity. The study questionnaire will also be administered in Swahili.

10.1136/bmjopen-2020-046664.supp2 Supplementary data

### Dissemination

Study findings will be shared in stakeholder meetings attended by local community leaders, the Ministry of Health, Community Development, Gender, Elderly and Children, the National Malaria Control Programme, the President’s office regional administration and local government representatives. Results will also be shared through peer-reviewed publications, at scientific conferences, and through clinicaltrials.gov.

## Supplementary Material

Reviewer comments

Author's manuscript
