# Supplementary material for: Protocol for a four parallel-arm, single-blind, cluster-randomised trial to assess the effectiveness of three types of dual active ingredient treated nets compared to pyrethroid-only long-lasting insecticidal nets to prevent malaria transmitted by pyrethroid insecticide-resistant vector mosquitoes in Tanzania
Source: BMJ Open. 2021 Mar 8;11(3):e046664. doi: 10.1136/bmjopen-2020-046664 (PMC7942254; doi:10.1136/bmjopen-2020-046664)
Supplement: Supplementary data [file bmjopen-2020-046664supp001.pdf]

Protocol for a four parallel-arm, single-blind, cluster-randomized trial to assess the effectiveness of three types of dual active ingredient treated nets compared to pyrethroid-only long-lasting insecticidal nets to prevent malaria transmitted by pyrethroid insecticide-resistant vector mosquitoes in Tanzania

Supplementary files

Methods

Ecological niche modelling

Suitability for each *Anopheles* species was determined using an ecological niche modelling approach, based on a maximum entropy (MaxEnt v.3.4.1) algorithm, similar to the approach described by Kulkarni et al. [1]. MaxEnt uses presence-only occurrence data in conjunction with environmental data to predict the potential distribution of a species across a defined landscape. Models were constructed for each major local species (*An. gambiae* s.s., *An. arabiensis* and *An. funestus* s.l.) using occurrence records from pilot mosquito monitoring alongside bioclimatic variables at a 1-km resolution from the WorldClim database (www.worldclim.org), including: temperature seasonality, annual precipitation, precipitation of wettest month, precipitation of driest month, and precipitation seasonality; and elevation from the Shuttle Radar Topography Mission (SRTM). To discriminate between sibling species of the An. gambiae s.l. complex, point locations where only An. gambiae s.s. (i.e. no An. arabiensis) was detected (n=12) were used as occurrence records. Point locations where >50% of the Anopheles mosquitoes were identified as An. funestus s.l. (n=51) were used as occurrence records for this species. Data were randomly partitioned for model evaluation, with 75% of the records used as training data to construct the models and the remaining 25% set aside for testing. The accuracy of each model was determined by performing both a threshold-dependent binomial test of omission and a threshold-independent receiver operating characteristic analysis; models with an area under the curve (AUC) >0.8 were retained, and the model with the highest AUC value for each species was used for subsequent analysis. The mean probability of suitability for each cluster was calculated using zonal statistics in ArcGIS.

Entomological data collection

Routine entomology collection

Cross-sectional entomological surveys will be carried out in 84 study clusters to monitor the indoor mosquito population density. Each cluster will be visited once every quarter; each month, 7 clusters from each study arm will be selected. Indoor mosquito densities will be monitored using Centers for Disease Control and Prevention (CDC) Miniature light traps (John W Hock Company, USA) in 8 randomly selected households in each of the cluster core areas. During the collection night, the owner's bed net in the bed/sleeping place where the CDC light trap will be installed will be substituted with a project standard LLIN, and replaced the following day. For each of the selected houses, a short questionnaire will be administered to collect information about the number of inhabitants, type of house construction materials, presence of animals, where the animals are kept, coverage and usage of study nets and other net types found, and other malaria prevention measures used by household members. Sampled mosquitoes will be identified morphologically following the identification key by Gillies and Coetzee [2]. Physiological status of *Anopheles* mosquitoes (blood-fed, unfed, gravid, semi-gravid) will be recorded. Parity rates will be estimated in a subset of live *Anopheles* mosquitoes through dissection [3]. Maximum 10 *An. gambiae* s.l and 10 *An. funestus* s.l will be randomly picked per household per collection night and preserved for molecular analysis. The mosquito specimens will be screened for *Plasmodium falciparum* circumsporozoite protein (Pf-CSP) by ELISA [4]. The CSP-ELISA positive samples will be re-analysed by heating the ELISA lysates to remove any false positives [5].

All specimens positive for Pf-CSP, as well as a subsample of CSP negative of *An. gambiae* s.l. and *An. funestus* s.l from each cluster per survey round, will be analysed for sibling species identification. PCR TaqMan assays will be used to discriminate members of *An. gambiae* species complex [6], and members of the *An. funestus* group [7]. A subsample of *An. gambiae* s.s. and *An. arabiensis* that are screened for malaria sporozoites infection will be genotyped for the L1014F-*kdr* and L1014S-*kdr* mutations, associated with pyrethroid and DDT resistance, using TaqMan PCR assays, following the protocol by Bass *et al* [8].

Insecticide resistance monitoring

Phenotypic resistance and resistance levels to alpha-cypermethrin, permethrin, and chlorfenapyr, will be characterized at baseline and yearly post-intervention, in all four study arms, using WHO cylinder and modified CDC bottle bioassays [9, 10]. The PBO synergist effect on wild female *An. gambiae* s.l. and *An. funestus* s.l. will be assessed using pre-exposure of PBO in CDC bottles followed by permethrin resistance intensity assays. The effect of PPF on fertility/egg development in adult female *Anopheles*, after 72 hours post-exposure to PPF using CDC bottle bioassays, will be assessed by ovarian dissection under a light microscope [11]. The developmental status of ovarian follicles/eggs will be classified as fertile or infertile and interpreted following the Christopher's stages of egg development [12, 13]. All assays will be performed using wild, indoor resting mosquitoes collected from house walls using

Prokopack and manual aspirators. Mosquitoes will be held for three days, fed on 10% glucose solution, to allow for blood-meal digestion, prior to bioassay testing. Mosquitoes will be morphologically identified as *An. gambiae* s.l. or *An. funestus* s.l. [2], and species complexes will be tested separately [6, 7]. All knockdown/dead mosquitoes at 60 minutes and surviving 72 hours post-exposure to insecticides will be stored individually in RNAlater® and preserved at -20°C for gene expression analysis. Per mosquito, 4-6 legs will be removed to identify individuals to species-level using TaqMan PCR [34-36]; only PCR-confirmed *An. gambiae* s.s. or *An. funestus* s.s., will be pooled separately for further analysis. RNA will be extracted from pools of *An. gambiae* s.s. or *An. funestus* s.s and cDNA synthesized, according to standard procedures. Relative expression of CYPs and other metabolic enzymes, previously identified as being over-expressed in resistant *An. gambiae* s.s. and *An. funestus* s.s. populations in Tanzania [14], will be measured using multiplex TaqMan RT-qPCR assays [15].

Mosquito behaviour

Malaria vector abundance, species composition, feeding and resting behaviours (including biting time, host preference), and contribution to outdoor malaria transmission will be assessed at baseline and after implementation of the interventions to assess changes over time. These mosquito behavioural adaptations will be assessed twice a year during the high mosquito density seasons (April-May and October-November)using CDC light traps indoor, occupied adapted Furvela tent traps outdoors [16] and indoor and outdoor resting collection using Prokopack aspirators. Collections will be done in 2 houses in 40 sentinel clusters (10 clusters per treatment arm) selected for their had high density of mosquitoes. In each household, light traps will be set up inside and one tent installed outside, close to the house. In the house were the mosquito traps will be installed, indoor resting mosquito collections will be conducted on the walls, roofs, and inside the nets. Similarly, outdoor collections of resting mosquitoes will be performed in potential resting sites around the house, such as open resting structures, cow sheds and pit latrines. A sub-sample of An. gambiae s.l. and An. funestus s.l. per each collection method will be tested for the presence of Pf-CSP [4] and identified to sibling species level [6, 7]. All freshly collected blood-fed Anopheles mosquitoes will be screened for the blood meal sources[17] .

The sterilizing effect of py-PPF ITN (Royal Guard®) relative to the standard LLINs on egg development in female wild malaria vectors will be assessed. This study will be conducted once a year in 10 sentinel clusters receiving Royal Guard, and 10 sentinel clusters receiving the standard LLIN. 10 houses per cluster will be visited, and sampled for freshly blood fed mosquitoes resting on the wall or inside the nets. Anopheles will be supplied with 10% glucose solution, monitored for survival and dissected 72 hours after collection to look at the developmental stages of eggs as a proxy of fertility [11]. Gravid alive females will be anesthetized at −20°C for 8–10 min and individually dissected in a drop of distilled water by gently pulling out the last two segments, 7th and 8th sternites of the mosquito abdomen.

References

1. Kulkarni MA, Desrochers RE, Kerr JT: **High resolution niche models of malaria vectors in northern Tanzania: a new capacity to predict malaria risk?** *PLoS One* 2010, **5:**e9396.

2. Gillies MTC, M.: *A supplement to the Anophelinae of Africa south of the Sahara (Afrotropical region).*1987.

3. Detinova T, Gillies M: **Observations on the determination of the age composition and epidemiological importance of populations of Anopheles gambiae Giles and Anopheles funestus Giles in Tanganyika.** *Bulletin of the World Health Organization* 1964, **30:**23.

4. Wirtz RA, Zavala F, Charoenvit Y, Campbell GH, Burkot TR, Schneider I, Esser KM, Beaudoin RL, Andre RG: **Comparative testing of monoclonal antibodies against Plasmodium falciparum sporozoites for ELISA development.** *Bull World Health Organ* 1987, **65:**39-45.

5. Durnez L, Van Bortel W, Denis L, Roelants P, Veracx A, Trung HD, Sochantha T, Coosemans M: **False positive circumsporozoite protein ELISA: a challenge for the estimation of the entomological inoculation rate of malaria and for vector incrimination.** *Malaria Journal* 2011, **10:**195.

6. Bass C, Williamson MS, Field LM: **Development of a multiplex real-time PCR assay for identification of members of the Anopheles gambiae species complex.** *Acta tropica* 2008, **107:**50-53.

7. Vezenegho SB, Bass C, Puinean M, Williamson MS, Field LM, Coetzee M, Koekemoer LL: **Development of multiplex real-time PCR assays for identification of members of the Anopheles funestus species group.** *Malaria Journal* 2009, **8:**1-9.

8. Bass C, Nikou D, Donnelly MJ, Williamson MS, Ranson H, Ball A, Vontas J, Field LM: **Detection of knockdown resistance (kdr) mutations in Anopheles gambiae: a comparison of two new high-throughput assays with existing methods.** *Malaria Journal* 2007, **6:**1-14.

9. Brogdon WG, Chan A: **Guideline for evaluating insecticide resistance in vectors using the CDC bottle bioassay.** Altlanta, USA: Centers for Disease Control and Prevention; 2012.

10. WHO: **Test procedures for insecticide resistance monitoring in malaria vector mosquitoes (Second edition).** Geneva, Switzerland: World Health organization; 2016.

11. Koama B, Namountougou M, Sanou R, Ndo S, Ouattara A, Dabire RK, Malone D, Diabate A: **The sterilizing effect of pyriproxyfen on the malaria vector Anopheles gambiae: physiological impact on ovaries development.** *Malar J* 2015, **14:**101.

12. Christophers SR: **The development of the egg follicles in anophelines.** *Paludism* 1911, **2:**73-88.

13. Koama B, Namountougou M, Sanou R, Ndo S, Ouattara A, Dabiré RK, Malone D, Diabaté A: **The sterilizing effect of pyriproxyfen on the malaria vector Anopheles gambiae: physiological impact on ovaries development.** *Malaria journal* 2015, **14:**101.

14. Matowo J, Kitau J, Kaaya R, Kavishe R, Wright A, Kisinza W, Kleinschmidt I, Mosha F, Rowland M, Protopopoff N: **Trends in the selection of insecticide resistance in Anopheles gambiae s.l. mosquitoes in northwest Tanzania during a community randomized trial of longlasting insecticidal nets and indoor residual spraying.** *Med Vet Entomol* 2015, **29:**51-59.

15. Mavridis K, Wipf N, Medves S, Erquiaga I, Muller P, Vontas J: **Rapid multiplex gene expression assays for monitoring metabolic resistance in the major malaria vector Anopheles gambiae.** *Parasit Vectors* 2019, **12:**9.

16. Charlwood JD, Rowland M, Protopopoff N, Le Clair C: **The Furvela tent-trap Mk 1.1 for the collection of outdoor biting mosquitoes.** *PeerJ* 2017, **5:**e3848.

17. Beier JC, Perkins PV, Wirtz RA, Koros J, Diggs D, Gargan TP, Koech DK: **Bloodmeal identification by direct enzyme-linked immunosorbent assay (ELISA), tested on Anopheles (Diptera: Culicidae) in Kenya.** *Journal of medical entomology* 1988, **25:**9-16.
