# Supplementary material for: Protocol for a four parallel-arm, single-blind, cluster-randomised trial to assess the effectiveness of three types of dual active ingredient treated nets compared to pyrethroid-only long-lasting insecticidal nets to prevent malaria transmitted by pyrethroid insecticide-resistant vector mosquitoes in Tanzania
Source: BMJ Open. 2021 Mar 8;11(3):e046664. doi: 10.1136/bmjopen-2020-046664 (PMC7942254; doi:10.1136/bmjopen-2020-046664)
Supplement: Author's manuscript [file bmjopen-2020-046664.draft_revisions.pdf]

BMJ Open

BMJ Open is committed to open peer review. As part of this commitment we make the peer review history of every article we publish publicly available.

When an article is published we post the peer reviewers' comments and the authors' responses online. We also post the versions of the paper that were used during peer review. These are the versions that the peer review comments apply to.

The versions of the paper that follow are the versions that were submitted during the peer review process. They are not the versions of record or the final published versions. They should not be cited or distributed as the published version of this manuscript.

BMJ Open is an open access journal and the full, final, typeset and author-corrected version of record of the manuscript is available on our site with no access controls, subscription charges or pay-per-view fees (http://bmjopen.bmj.com).

If you have any questions on BMJ Open's open peer review process please email info.bmjopen@bmj.com

BMJ Open

Protocol for a four parallel-arm, single-blind, cluster-randomized trial to assess the effectiveness of three types of dual active ingredient treated nets compared to pyrethroid-only long-lasting insecticidal nets to prevent malaria transmitted by pyrethroid insecticide-resistant vector mosquitoes in Tanzania

Journal:	*BMJ Open*
Manuscript ID	bmjopen-2020-046664
Article Type:	Protocol
Date Submitted by the Author:	05-Nov-2020

Complete List of Authors:	Mosha, Jacklin; National Institute for Medical Research Mwanza Research Centre,<br>Kulkarni, Manisha; University of Ottawa Faculty of Medicine, Epidemiology & Public Health<br>Messenger, Louisa; London School of Hygiene & Tropical Medicine, Disease Control<br>Rowland, Mark; London School of Hygiene & Tropical Medicine, Disease Control<br>Matowo, Nancy; London School of Hygiene & Tropical Medicine, Disease Control<br>Pitt, Catherine; London School of Hygiene & Tropical Medicine, Global Health and Development<br>Lukole, Eliud; National Institute for Medical Research Mwanza Research Centre; London School of Hygiene & Tropical Medicine, Disease Control<br>Taljaard, Monica; Ottawa Hospital Research Institute, Clinical Epidemiology Program<br>Manjurano, Alphaxard; National Institute for Medical Research Mwanza Research Centre<br>Mosha, Franklin; Kilimanjaro Christian Medical University College<br>Kleinschmidt, Immo; London School of Hygiene & Tropical Medicine<br>Protopopoff, Natacha; London School of Hygiene & Tropical Medicine, Disease Control

Keywords:	PARASITOLOGY, INFECTIOUS DISEASES, Infection control < INFECTIOUS DISEASES, Epidemiology < INFECTIOUS DISEASES, HEALTH ECONOMICS, Public health < INFECTIOUS DISEASES

SCHOLARONE™
Manuscripts

I, the Submitting Author has the right to grant and does grant on behalf of all authors of the Work (as defined in the below author licence), an exclusive licence and/or a non-exclusive licence for contributions from authors who are: i) UK Crown employees; ii) where BMJ has agreed a CC-BY licence shall apply, and/or iii) in accordance with the terms applicable for US Federal Government officers or employees acting as part of their official duties; on a worldwide, perpetual, irrevocable, royalty-free basis to BMJ Publishing Group Ltd ("BMJ") its licensees and where the relevant Journal is co-owned by BMJ to the co-owners of the Journal, to publish the Work in this journal and any other BMJ products and to exploit all rights, as set out in our licence.

The Submitting Author accepts and understands that any supply made under these terms is made by BMJ to the Submitting Author unless you are acting as an employee on behalf of your employer or a postgraduate student of an affiliated institution which is paying any applicable article publishing charge ("APC") for Open Access articles. Where the Submitting Author wishes to make the Work available on an Open Access basis (and intends to pay the relevant APC), the terms of reuse of such Open Access shall be governed by a Creative Commons licence – details of these licences and which Creative Commons licence will apply to this Work are set out in our licence referred to above.

Other than as permitted in any relevant BMJ Author's Self Archiving Policies, I confirm this Work has not been accepted for publication elsewhere, is not being considered for publication elsewhere and does not duplicate material already published. I confirm all authors consent to publication of this Work and authorise the granting of this licence.

For peer review only

Protocol for a four parallel-arm, single-blind, cluster-randomized trial to assess the effectiveness of three types of dual active ingredient treated nets compared to pyrethroid-only long-lasting insecticidal nets to prevent malaria transmitted by pyrethroid insecticide-resistant vector mosquitoes in Tanzania

Jacklin F. Mosha[1]*, Manisha A. Kulkarni[2], Louisa A. Messenger[3], Mark Rowland[3], Nancy Matowo[3], Catherine Pitt[4], Eliud Lukole[1], Monica Taljaard[2,5], Alphaxard Manjurano[1], Franklin W Mosha[6], Immo Kleinschmidt[7,8,9], Natacha Protopopoff[3]

1. National Institute for Medical Research, Mwanza Medical Research Centre, Mwanza, Tanzania
2. School of Epidemiology and Public Health, University of Ottawa, Ottawa, Canada
3. Department of Disease Control, London School of Hygiene and Tropical Medicine, London, United Kingdom
4. Department of Global Health and Development, London School of Hygiene and Tropical Medicine, London, United Kingdom
5. Ottawa Hospital Research Institute, Ottawa, Canada
6. Kilimanjaro Christian Medical University College, Moshi, Tanzania
7. MRC Tropical Epidemiology Group, Department of Infectious Disease Epidemiology, London School of Hygiene and Tropical Medicine, London, United Kingdom

For peer review only

8. Wits Research Institute for Malaria, School of Pathology, Faculty of Health Sciences, University of the Witwatersrand, Johannesburg, South Africa.

9. Southern African Development Community Malaria Elimination Eight Secretariat, Windhoek, Namibia.

* Corresponding Author

Email: jfmosha@yahoo.com

Tel: +255 788 033 605

Word count: 5527

Funding: The Department of Health and Social Care, the Department for International Development, the Medical Research Council and the Wellcome Trust

Abstract

Introduction: The massive scale-up of long-lasting insecticidal nets (LLINs) has led to major reductions in malaria burden in many sub-Saharan African countries. This progress is threatened by widespread insecticide resistance among malaria vectors. This cluster randomized controlled trial (c-RCT) compares three of the most promising dual active ingredients LLINs (Dual AI LLINs), which incorporate mixtures of insecticides or insecticide synergists to standard LLINs in an area of pyrethroid insecticide resistance.

For peer review only

Methods: A four-arm, single-blinded, c-RCT will evaluate the effectiveness of three types of Dual-AI LLINs 1) Royal Guard®, combining two insecticides, pyriproxyfen (PPF) and the pyrethroid alpha-cypermethrin; 2) Interceptor® G2, combining chlorfenapyr and alpha-cypermethrin3) Olyset® Plus, a LLIN combining a synergist, piperonyl butoxide (PBO) and the pyrethroid permethrin, compared to 4) Interceptor® LN, a standard LLIN containing the pyrethroid alpha-cypermethrin as the sole AI. The primary outcomes are malaria infection prevalence in children aged 6 months to 14 years and entomological inoculation rate (EIR), as a standard measure of malaria transmission at 24 months post intervention and cost-effectiveness.

Ethics and dissemination: Ethical approval was received from the institutional review boards of the Tanzanian National Institute for Medical Research, Kilimanjaro Christian Medical University College, London School of Hygiene and Tropical Medicine, and University of Ottawa. Study findings will be actively disseminated via reports and presentations to stakeholders, local community leaders, and relevant national and international policy makers as well as through conferences, and peer-reviewed publications.

Trial registration number: NCT03554616

For peer review only - http://bmjopen.bmj.com/site/about/guidelines.xhtml

Strengths and limitations of this study

- This study is the first RCT to evaluate and compare the effectiveness of the next generation of dual-treated LLINs, Royal Guard® (a pyrethoid-pyriproxifen LLIN) and Interceptor® G2 (a pyrethroid-chlorfenapyr LLIN) against standard LLIN to prevent malaria infection prevalence and incidence in an area of pyrethroid insecticide resistance.
- Results of this study will be presented to the World Health Organization for policy recommendations and depending on the study outcomes has the potential to shape malaria vector control strategies across sub-Saharan Africa for the next decade.
- This is the second RCT to evaluate the new class of pyrethroid-PBO LLINs (Olyset® Plus) in the Great Lakes Zone of Tanzania, expanding the evidence basis for deployment of this intervention class in areas of pyrethroid resistance.
- The trialling of the three main categories of Dual-AI LLN against the standard pyrethroid LLIN in the same human cultural community against the same vector complex should offer the best opportunity to unravel relative effectiveness on malaria and effect on selection of insecticide resistance.
- Some limitations include the size of the cluster buffer areas, which might not prevent all contamination between intervention arms, and the use of malaria rapid diagnostic tests to assess malaria infections rather than double-read blood slides, which may be more sensitive.

For peer review only

Introduction

Long-lasting insecticidal nets (LLINs) are the primary method of malaria control in Sub-Saharan Africa. The World Health Organization (WHO) estimates that over 50% of the population of Sub-Saharan Africa now sleep under LLINs.[1] Together with improved diagnosis and treatment, LLINs have helped reduce malaria incidence by 42% and mortality by 66% in Africa in the last 15 years.[1] Pyrethroids are the only class of insecticide used routinely on LLINs, so the rapid spread of pyrethroid resistance across vector populations threatens to reverse the successes achieved so far, and may be a factor contributing to the current stagnation in malaria disease burden.[2]

Several studies have demonstrated that LLINs are becoming less effective at killing mosquitoes in areas of moderate to high pyrethroid resistance compared to those with susceptible vector populations.[3-8] The search for new insecticides suitable for LLIN treatment is vital to sustain effective malaria vector control.

During the last decade, the WHO has encouraged manufacturers to develop new types of bed nets to control resistant mosquitoes. The chemical industry responded initially by developing a Dual AI LLIN, combining a pyrethroid insecticide with the synergist piperonyl butoxide (PBO), which inhibits cytochrome P450 oxidases responsible for pyrethroid resistance. Pyrethroid-PBO LLINs (py-PBO LLINs) have been available since 2009,[9] but had only been recommended by WHO for limited deployment until[10] a recent cluster-randomized controlled trial (RCT) demonstrated a 44% reduction in malaria infection prevalence in children in the py-PBO LLIN arm (Olyset™ Plus) compared to the standard pyrethroid LLIN (s-LLIN) after two years.[8] In 2017, based on this study, the WHO recognised the public health value of this

For peer review only

new class of LLIN and recommended their scale up in area with pyrethroid resistance.[11] A second large-scale RCT in Uganda confirmed the superior protection of py-PBO LLIN against malaria compared to s-LLINs.[12]

Other manufacturers have responded to the WHO call by producing a different kind of Dual-AI LLIN that incorporates a mixture of insecticides from different insecticide classes. Mixtures of two insecticides on the same LLIN with differing modes of action have the potential to delay the evolution of resistance and extend the lifespan of both active ingredients on the LLIN. The two latest products are a pyrethroid-pyriproxyfen LLIN (py-PPF LLIN: Olyset® Duo and Royal Guard®) and a pyrethroid-chlorfenapyr LLIN (py-CFP LLIN: Interceptor® G2). Both types of product have shown superior efficacy compared to s-LLINs in small-scale experimental hut trials.[13-20] The Py-PPF LLINs have demonstrated enhanced efficacy vector oviposition suppression and up to 95% reduction in vector reproductive rate in Benin.[14 21] In addition, Py-PPF LLIN Olyset® Duo, when compared with s-LLINs in an RCT in Burkina Faso, had a significantly greater impact on clinical malaria.[22] The py-CFP LLIN, when compared to s-LLINs, has shown enhanced efficacy through higher killing against resistant mosquito species in experimental hut trials, producing mortalities with *An. gambiae* s.l. of 71% *vs*. 20% in Benin[20] and 78% *vs*. 17% in Burkina Faso,[18] and 71% *vs*. 45% with *An. arabiensis*[23] and 70% *vs*. 37% with *An. funestus* s.l. in Tanzania.[24] Neither of these new AIs, pyriproxifen or chlorfenapyr, are related to one another nor show cross resistance. However, to receive a WHO public health recommendation, these "next-generation" LLINs still need to be evaluated in two RCT[25] to demonstrate their effectiveness against malaria in human populations in areas characterized by different insecticide resistance intensities and major vector species.

For peer review only

In Tanzania, insecticide resistance has spread rapidly[26-28]. In regions where resistance is particularly strong, such as North-West Tanzania, the prevalence of malaria infection remains high (40% in children under 5 years old), despite universal coverage of s-LLINs.[29] This finding echoes reports from Uganda, where no reduction in malaria incidence was observed after the distribution of s-LLINs.[30] As operational failure of standard nets is occurring more frequently in areas with pyrethroid resistance, including the Great Lakes Zone,[30] the newly-developed "next-generation" LLINs now require urgent comparative evaluation.

Here we describe the study design and methodology of an RCT, assessing the effectiveness and cost-effectiveness of three novel vector control interventions (Dual-AI LLINs incorporating mixtures of insecticide classes or insecticide synergists), compared to the standard best practice of pyrethroid-only LLINs, to prevent malaria in an area of pyrethroid resistance in Tanzania. Each putative new class needs to show high effectiveness versus the s-LLIN. None should select for stronger resistance to pyrethroid. Ideally, none should select for resistance to the other new classes of Dual-AI LLIN being tested. A four arm trial is a highly efficient design, should demonstrate the relative effectiveness of each against malaria, and the merits of each product against the same fauna of vector species and human cultural group. The trial should provide insight into future rotational strategies of deployment, their potential to manage insecticide resistance while controlling malaria.

The durability and bio-efficacy of the Dual-AI LLIN are also being evaluated as per WHO guidelines[31] and will be presented in a separate protocol, published elsewhere. The study protocol is reported in line with the Standard Protocol Items: Recommendations for Interventional Trials (SPIRIT) 2013 Statement.[32]

For peer review only - http://bmjopen.bmj.com/site/about/guidelines.xhtml

Study Objectives

The primary clinical and entomological objectives are to assess the effectiveness of py-PPF-LLINs, py-CFP-LLINs and py-PBO-LLINs compared to s-LLINs:

- On malaria infection prevalence in children from 6 months to 14 years over two years post intervention.
- On the entomological inoculation rate (EIR) of malaria vectors collected indoors over two years post intervention.

The primary economic objective is to assess the cost-effectiveness of py-PPF-LNs, py-CFP-LNs, py-PBO-LNs and s-LLINs relative to one another.

All secondary objectives are detailed in Figure 1.

Methods and Analysis

Study setting

Five potential study sites in Tanzania's Victoria lake zone were evaluated based on four criteria: report of a minimum of 30% malaria infection prevalence in total population, *An. gambiae* sensu stricto (s.s.) or *An. funestus* s.s. as the main vectors, insecticide pyrethroid resistance in standard WHO bioassays (<50% mortality), and no indoor residual spraying (IRS) planned for the next three years. Data from published and unpublished sources (including the National Malaria Control Program, NMCP; and the President's Malaria Initiative, PMI) were examined and complemented with data collection in November 2017, as appropriate. Only the

For peer review only - http://bmjopen.bmj.com/site/about/guidelines.xhtml

district of Misungwi (2°51'00.0"S, 33°04'60.0"E), on the southern border of Lake Victoria in Tanzania met all the criteria.

Misungwi covers an area of 2,122km^2 and includes 27 wards, 78 villages, and a population of 351,607 people.[33] Average altitude in the study area is 1150 meters. The annual rainfall ranges from 0.5 mm to 58.8 mm, split in two rainy seasons (October-December and March-May) and interrupted by a distinct long dry season (June to August/September) and a second short dry season in late December-February.[34]

Misungwi has moderate to high malaria transmission. In a study conducted in 2010, prevalence was 52% across all age groups.[35] During the preliminary assessment in May 2018, presence of all main Tanzanian malaria vectors, *An. gambiae* s.s., *An. arabiensis* and *An. funestus* s.s., were found in the area (unpublished data). WHO insecticide resistance tests were also performed and 24-hour mortality in wild caught *An. gambiae* s.s. exposed to permethrin and deltamethrin was 7% and 19%, respectively. There was also evidence of pyrethroid resistance in *An. funestus* s.s. (mortality 50%) and *An. arabiensis* (mortality 65.3%). Pyrethroid insecticide resistance has also been observed in the adjacent district of Magu and other lake zone regions, such as Geita and Kagera.[28]

The main vector control interventions in Misungwi are universal coverage of LLINs and IRS. The last distribution of LLINs and IRS campaign using Actellic 300CS were carried out in Misungwi district in 2015. In 2017, larviciding was done in some parts of the district.[36] The district is also following national malaria control measures such as intermittent preventive treatment of malaria in pregnant women.

For peer review only - http://bmjopen.bmj.com/site/about/guidelines.xhtml

Study design

An overview of the trial design is given in Figure 2. The design is a four parallel-arm, single-blind, superiority cluster randomised trial with village hamlet as the unit of randomisation and repeated cross-sectional survey. The four study arms are:

1. Mixture py-PPF-LLIN: Royal Guard® [intervention 1]
2. Mixture py-CFP-LLIN: Interceptor® G2 [intervention 2]
3. Py-PBO-LLIN: Olyset™ Plus [intervention 3]
4. S-LLIN: Interceptor® [control arm]

The inhabitants of each cluster are blinded to the type of nets they received, as are the field staff who will collect entomological and clinical data. Nets of each type are similar in appearance apart from a colour-coded loop and a unique identifying number. Only the principal investigator and data manager know which code represents each intervention.

Mapping and cluster formation

Every building of 72 villages comprising 453 hamlets from 17 wards was mapped using a Global Positioning System (GPS) handheld unit (Garmin Legend e-trex) and ExpertGPS v3.8 (TopoGrafix) software. A short Open Data Kit (ODK) programmed questionnaire, including name of the head of the household, number of people living in the house, and number of children in each age group (6 months to 59 months, 5 years to 10 years, 11 to 14 years) was recorded for each mapped household.

Wards to map were selected based on both malaria incidence data collected from all health facilities in Misungwi, and *Anopheles* species composition (only areas with the main malaria

For peer review only

vectors *An. funestus* s.s. and *An. gambiae* s.s were considered), assessed during pilot mosquito monitoring from all villages.

As in previous studies,[8] clusters were designed with core and buffer areas to reduce the likelihood of spill-over of intervention effects from one cluster to another. Nets will be distributed to all households in a given cluster (i.e. core and buffer areas), but monitoring of outcomes will be restricted to households situated in the cluster core. A total of 86 clusters were formed from the 72 villages (Figure 3) using the spatial analyst toolbox in ArcGIS (ESRI, Redlands, USA) based on the following criteria: no sub-division of village hamlets, minimum of 150 households with children aged 6 months to 14 years in the core area, and a minimum 600-meter buffer distance. The number of households in each cluster varied from 172 to 2390 (urban area of Misungwi) with an average of 492 households. A 600-meter buffer (i.e. 600 meters between the margins of core areas for any two adjacent clusters) was allocated thereby retaining 73.6% (31,125/42,314) of the households in the core area for sampling and data collection, with 134 to 828 core area households per cluster (Figure 3, panel C). Only two clusters did not meet the criteria of 150 households in the core area, and were therefore excluded.

Randomisation

After the completion of the baseline survey, covariate constrained randomisation was used to allocate the 84 clusters across the four study arms. Covariate constrained allocation ensures that the arms are balanced overall by excluding allocations where pre-determined factors are not balanced within set margins.[37] The following factors were constrained: baseline (pre-intervention) malaria infection prevalence in children aged 0.5 to 14 years, previous LLIN

For peer review only - http://bmjopen.bmj.com/site/about/guidelines.xhtml

usage, socio-economic status (SES), population size, suitable conditions for *An. gambiae* s.s., and for *An. funestus* s.l.. Suitability for each *Anopheles* species was determined using an ecological niche modelling approach (supplemental material) as it was not possible to assess the species composition in all clusters before the randomisation. From among all possible allocations meeting the balancing constraints, one allocation was selected at random. The randomisation was performed by independent statistician.

Interventions

In all four trial arms, net distribution was carried out in an identical manner by the Tanzania Communication and Development Center (TCDC), a Tanzanian not-for-profit organisation, and supervised by the NMCP to follow national net distribution campaign guidelines.[38] All households, enumerated in the core and buffer area, were allocated one net for every two people as recommended by the Tanzania NMCP.[38] Information, education and communication (IEC/SBCC) activities were conducted by a Tanzanian NGO, Tulonge Afia, before, during and after the net distribution to increase usage in the study area and supported by the US President Malaria Initiative. Householders were not asked to return their old net but to use the new net provided. To maximize effective LLIN coverage, two door-to-door hang-up campaigns were done after two weeks and again three months after distribution.

All nets distributed were blue and rectangular (180 cm length x 160 cm wide x 180 cm high). They differed by study arm as follows:

Control: Interceptor® LN (BASF Corporation, Germany) is a pyrethroid-only LLIN with alpha-cypermethrin at a target dose of 200 mg/m2, coated onto polyester filaments. Pyrethroids are neurotoxic insecticides, which target the nervous system of insects. Interceptor® LN was

For peer review only

chosen as a direct comparison to Interceptor® G2 LN and Royal Guard® LN, as they are all impregnated with the same pyrethroid (alpha-cypermethrin); some insecticide resistance mechanisms involving CYPs are specific to type I (permethrin) or type II (alpha-cypermethrin) pyrethroids.

Intervention 1: Royal Guard® LN (Disease Control Technologies, LLC) is a mixture LLIN made of polyethylene incorporating 225 mg/m^2 pyriproxyfen and 216 mg/m^2 alpha-cypermethrin. PPF is known to disrupt female mosquito reproduction and fertility of eggs, stopping the production of the next generation of mosquitoes.[39] PPF may be transferred by auto-dissemination; the transfer of small but toxic amounts of this highly potent insecticide on the tarsi (feet) of female mosquitoes to breeding sites where it can also act as a larvicide.[40]

Intervention 2: Interceptor® G2 LN is a mixture LLIN made of polyester coated with a wash-resistant formulation of 200 mg/m^2 chlorfenapyr and 100 mg/m^2 alpha-cypermethrin. Chlorfenapyr disrupts cellular respiration and oxidative phosphorylation in mitochondria, and due to this unique mode of action is toxic against mosquitoes that are resistant to standard neurotoxic insecticides like pyrethroids.[41]

Intervention 3: Olyset® Plus LN (Sumitomo Chemical) is a mixture LLIN combining PBO (400mg/m^2) and the repellent pyrethroid permethrin (800 mg/m^2) incorporated into polyethylene fibres. PBO is a chemical synergist which acts by inhibiting mixed function oxidases, preventing detoxification of the pyrethroid insecticide. Two RCTs demonstrated that Olyset® Plus LLINs were more effective than Olyset® Net LN (the standard of care in Tanzania), in areas with pyrethroid resistance.[8 12]

Study outcomes

For peer review only - http://bmjopen.bmj.com/site/about/guidelines.xhtml

Primary outcomes

The primary outcomes (Table 1) will be

- Malaria infection (by mRDT) in children aged 6 months to 14 years old at 24 months post intervention.
- Entomological inoculation rate (EIR) as a measure for malaria transmission in the primary vector species.
- Cost-effectiveness of each of the four net types relative to one another.

Secondary outcomes

- Incidence of malaria cases in children aged 6 months to 10 years (measured over 24 months follow up).
- Moderate and severe anaemia in children under 5 years old (<8 g/dL) at 12, 18, 24, 30 and 36 months.
- Malaria infection (by mRDT) in children aged 6 months to 14 years at 12, 18, 30 and 36 months.
- Changes in frequency and intensity of phenotypic and genotypic resistance to pyrethroids (alpha-cypermethrin and permethrin), PBO, CFP and PPF.
- Other entomological outcomes: changes in mosquito resting behaviour indoors and outdoors, species composition and density ratio, host feeding, mosquito ovary development and fecundity.
- Incremental financial cost to the provider of each of the four net types relative to one another.

For peer review only - http://bmjopen.bmj.com/site/about/guidelines.xhtml

Data collection

Intervention coverage data

LLIN coverage will be evaluated 6 months after distribution and during each cross-sectional survey. Three indicators will be used:[42] 1)"proportion of households with at least one LLIN for every two people(ownership)", 2)"proportion of household with enough LLINs to sleep under (access)" and "proportion of households declaring using a LLIN (study or not) last night (usage)".

Clinical data

To determine infection prevalence, repeated cross-sectional surveys will be conducted at 12, 18, 24, 30 and 36 months after net distribution (Table 1). At each time point, 45 households will be randomly selected from the core sampling area of each cluster, using the census list generated during baseline enumeration. In each house a maximum of two children, between 6 months and 14 years old, will be selected. Accounting for households that are closed, refusing informed consent, or do not have children of the required age at the time of the survey, this sampling strategy is expected to yield an average of 30 enrolled houses with 50 children per cluster based on previous studies. [8 43] A total of 4200 children are thus expected to be surveyed at each time point.

Inclusion criteria are households with at least one child between 6 months to 14 years old who permanently resides in the selected household and an adult caregiver who can provide written consent. Exclusion criteria include dwellings not found or vacant during the survey, no adult caregiver capable of giving informed consent, or eligible children are severely ill.

For peer review only - http://bmjopen.bmj.com/site/about/guidelines.xhtml

Information on sex, age distribution, educational status and occupation, SES, house structure, vector control measures used, past malaria cases, net coverage and care seeking behaviour will be collected. Fever or history of fever in the past 48 hours will be recorded for every child selected. Temperatures will be taken, and each child tested for malaria using mRDTs, (CareStart RDTs; HRP2, (pf), DiaSys, Wokingham, UK) and haemoglobin levels measured (HemoCue Hb 201+ (Aktiebolaget Leo Diagnostics, USA)). When the mRDT is positive, free treatment for malaria will be provided with artemether-lumefantrine (artemisinin-based combination therapy; ACT), as per the national guidelines. Children with severe malaria or any other diseases that cannot be treated by the team will be referred to the nearest health facility.

To assess malaria case incidence, 35 children per cluster, aged 6 months to 10 years old, will be randomly selected and followed up every two weeks during the high transmission season (October-July) and every month during the low transmission season (August-September), over a 12 months period (Table 1). To reduce attrition, another cohort of children will be selected at the beginning of the second year and will also be followed for 12 months. All cohort children will be cleared of malaria infection at the beginning of the first and second year by ACT treatment. Cohort children will then be checked two weeks later by mRDT and microscopy (if the mRDT is positive) to confirm whether they have been cleared. During each bi-weekly or monthly visit, children with fever ≥37.5°C and/or history of fever for the past 48 hours will be tested for the presence of malaria parasites (case incidence) by mRDT. For those with positive mRDT results, a blood slide will also be taken to confirm malaria positivity and they will also be treated, as previously described. Children will be encouraged to visit a health facility in case they have fever, or get sick at any time between visits. Each child will be provided with a personal medical book where malaria episodes will be recorded during study visits or whenever

For peer review only

they attend a health facility and also will be provided with medical insurance. During cohort visits, a questionnaire will also be administered to inquire about net usage the night before the visit, any adverse events encountered and travel history within the past two weeks.

Entomological data

Cross-sectional entomological surveys will be carried out in 84 study clusters to monitor the indoor mosquito population density (Table 1). Each cluster will be visited once every quarter; each month, 7 clusters from each study arm will be selected. Indoor mosquito densities will be monitored using Centers for Disease Control and Prevention (CDC) Miniature light traps (John W Hock Company, USA) in 8 randomly selected households in the core area of each cluster. CDC light trap will be installed at the feet of one bed and existing net substituted with a project standard LLIN, and replaced the following day. For each of the selected houses, a short questionnaire will be administered to collect information about the number of inhabitants, type of house construction materials, presence of animals, coverage and usage of nets, and other malaria prevention measures used by household members. Sampled mosquitoes will be identified morphologically following the identification key by Gillies and Coetzee.[44] Parity rates will be estimated in a subset of live *Anopheles* mosquitoes through dissection.[45] About 10 *An. gambiae* s.l and 10 *An. funestus* s.l will be randomly picked per household per collection night and preserved for laboratory analysis. This sample of mosquito specimens will be screened for *Plasmodium falciparum* circumsporozoite protein (Pf-CSP) by ELISA.[46] The CSP-ELISA positive samples will be re-analysed by heating the ELISA lysates to remove any false positives.[47]

For peer review only - http://bmjopen.bmj.com/site/about/guidelines.xhtml

PCR TaqMan assays will be used to discriminate members of *An. gambiae* species complex,[48] and members of the *An. funestus* group[49] on a sub sample of *Anopheles* collected. A sample of *An. gambiae* s.s. and *An. arabiensis* will be genotyped for the L1014F-*kdr* and L1014S-*kdr* mutations, associated with pyrethroid and DDT resistance, using TaqMan PCR assays, following the protocol by Bass *et al*.[50]

Phenotypic resistance and resistance levels to alpha-cypermethrin, permethrin, pyriproxyfen and chlorfenapyr, will be characterized at baseline and yearly post-intervention, in all four study arms, using WHO cylinder and modified CDC bottle bioassays.[51 52] The PBO synergist effect on wild female *An. gambiae* s.l. and *An. funestus* s.l. will be assessed using pre-exposure to PBO followed by permethrin resistance intensity assays. All knockdown/dead mosquitoes at 60 minutes and surviving 72 hours post-exposure to insecticides will be stored individually in RNAlater® and preserved at -20°C for gene expression analysis. RNA will be extracted from pools of *An. gambiae* s.s. or *An. funestus* s.s and cDNA synthesized, according to standard procedures. Relative expression of CYPs and other metabolic enzymes, previously identified as being over-expressed in resistant *An. gambiae* s.s. and *An. funestus* s.s. populations in Tanzania,[53] will be measured using multiplex TaqMan RT-qPCR assays.[54]

Malaria vector abundance, species composition, feeding and resting behaviours (including biting time, host preference), and contribution to outdoor malaria transmission will be assessed at baseline and after implementation of the interventions to assess changes over time in 10 clusters per treatment arm. CDC light trap and adapted Furvela tent traps[55] will be used for indoor and outdoor collection of free flying mosquitoes. Resting *Anopheles* will be collected using CDC prokopack aspirators. Sub samples of *Anopheles* will be subjected to the same

For peer review only - http://bmjopen.bmj.com/site/about/guidelines.xhtml

laboratory tests than routine collection. Additional information about mosquito collection methods is available in the supplemental material.

Economic data

Data on resource use and unit costs will be collected from primary and secondary sources, including discussions with net manufacturers, donors, and experts; review of project, donor, and Ministry of Health records; trial survey data on care seeking behaviour; published literature; and WHO-CHOICE unit cost estimates.[56] Data on health outcomes will include primary trial data on malaria incidence and anaemia, as well as secondary data on the age structure of malaria incidence and life expectancy.[57 58]

For peer review only

For peer review only - http://bmjopen.bmj.com/site/about/guidelines.xhtml

Table 1: Trial outcome measurements

Outcome	Measurement	Collection	Frequency
**Clinical outcomes**			
Malaria infection prevalence	Rapid diagnostic test	Cross sectional survey	Baseline, 12, 18, 24, 30, and 36 months post intervention
Anaemia	Haematocrit	Cross sectional survey	Baseline, 12, 18, 24, 30, and 36 months post intervention
Temperature	1.Digital ear thermometer all children<br>2.Temperature and history of fever	1.Cross sectional survey<br>2.Cohort follow up	1.Baseline, 12, 18, 24, 30 and 36 months post intervention<br>2.Every month
Malaria case	Rapid diagnostic test taken when fever ≥37.5°C and or history of fever for the past 48 hours	Cohort follow up	Every two weeks during high transmission season (October-July) and every month during dry season (August-September)
**Measurement of entomological outcomes**			
Indoor *Anopheles* density	CDC light traps	Entomology surveillance	8 houses per cluster every 3 months in all clusters for 3 years
Outdoor biting	Adapted Furvela tent trap[55]	Sentinel site	Twice a year in 2-3 sentinel site per arm.
Mosquito sporozoite rate	CSP-ELISA to estimate EIR[46]	Entomology surveillance	Sub sample (30%) of the mosquito collected in CDC light trap
Anopheles species identification	*1.An. funestus* s.l. complex: conventional PCR or multiplex real time PCR[49]<br>*2.An. gambiae* s.l. complex: Taq Man real time PCR[48]	Entomology surveillance & Sentinel site & resistance test	Sub sample of mosquitoes collected
Insecticide resistance frequency and intensity	WHO cylinder assay[52]<br>CDC bottle bioassays[51]	Collection of adult *Anopheles* resting indoors	Once a year in a sub sample of clusters.
Frequency of Vgsc mutation	TaqMan PCR[50]	Entomology surveillance	Sub sample of mosquitoes collected in light trap
Insecticide resistance mechanisms	Multiplex TaqMan Reverse-transcription quantitative PCR (RT-qPCR) will be used to monitor expression of CYPs and other metabolic enzymes known to be over-expressed in resistant *An. gambiae* s.s. and *An. funestus* s.s. populations from previous studies in Tanzania[54]	Collection of adult *Anopheles* resting indoors, previously phenotyped in resistance bioassays	At baseline and at each post intervention year in a sub sample of clusters

For peer review only

For peer review only - http://bmjopen.bmj.com/site/about/guidelines.xhtml

Sample size and power consideration

Malaria prevalence cross-sectional survey

The sample size was calculated using the method of Hayes and Bennett, taking into account the cluster-randomised design.[59] For the primary outcome prevalence of infection at 24 months, we assumed a malaria prevalence in the reference arm of 40%, an average of 50 individuals per cluster, and a coefficient of variation of 21%, based on recent surveys in a similar area.[60] To achieve 80% power to detect a prevalence ratio of 0.72, i.e, a 28% lower prevalence in at least one of the intervention arms versus the reference at Bonferroni-corrected significance level of 1.67%, we require 21 clusters per arm. This calculation is conservative as it does not account for the repeated malaria prevalence measures; thus, we anticipate being able to detect even smaller differences.

Incidence of malaria infection in children cohort

Sample size calculations for the secondary outcome (cumulative malaria incidence) were based on the method of Hayes and Bennett.[59] Based on a previous study (Jacklin Mosha, personal communication), it was assumed that the mean number of malaria episodes per child per year in the reference arm was 0.85 (monthly event rate of 0.071) with a between-cluster coefficient of variation of 21%. With a cohort of 35 children per cluster (21 clusters per arm), and accounting for attrition of 30% over 24 months, we would achieve 80% power to detect a 23.6% relative reduction in malaria cases per child per year (risk ratio 0.764) between at least one intervention arm relative to the reference arm, using a two-sided Bonferroni-corrected

For peer review only - http://bmjopen.bmj.com/site/about/guidelines.xhtml

significance level of 1.67%. If the incidence in the reference arm is lower, at 0.5 episodes/year, we will still have 80% power to detect a 26.1% relative reduction (relative risk 0.739).

Entomological survey for EIR estimation

Sample size calculations for the entomological survey were based on the method of Hayes and Bennett.[59] It was assumed that the mean EIR (number of infectious bites per household per month) in the reference arm was 1.76 with a between-cluster coefficient of variation of 40%, based on a previous study.[8] With a sample of 8 households per cluster and sampling each cluster every quarter we will have collections for 32 house-nights per cluster per year. With 21 clusters per arm the study would achieve 80% power to detect a 36% relative reduction in monthly EIR (relative risk 0.64) between at least one of the intervention arms relative to the reference using a Bonferroni-corrected significance level of 1.67%. Changes in insecticide resistance frequency and resistance management potential will be assessed separately from light trap and household resting collections.

Data Management

Clinical and entomological measurements in the cohort study and the cross-sectional surveys will be captured on electronic forms using tablets/smartphones installed with open data kit (ODK) and uploaded daily onto the server at London School Hygiene and Tropical Medicine (LSHTM). Other data and clinical measurements during the cross-sectional survey, recorded on paper CRFs, will be double-entered into an access database independently by two data clerks. Laboratory data outputs will be available directly from the analyser (e.g. ELISA data) and imported into a database. All databases will maintain an audit trail with time-date stamps

For peer review only

of data entry and all changes that are made to the data. Anonymized study numbers will be used as unique participant identifiers.

Data analysis

Primary outcomes

Prevalence of malaria infection and entomological inoculation rate

The primary analysis will be conducted using the intention to treat (ITT) approach. Secondary per-protocol analyses will also be conducted. The primary outcome, measured at 12,18,24,30 and 36 months after net distribution, will be analysed using mixed effects logistic regression. The unit of analysis will be the individual. The model will include fixed effects for time, study arm, and time by study arm, and will adjust for the baseline prevalence in each community as a covariate, as well as the covariates used in the covariate-constrained allocation procedure. The model will account for within-period and between-period intra-cluster correlations.[61]

Prevalence of malaria infection in each Dual-AI LLIN treatment arm will be compared to the prevalence of infection in the reference arm using least square mean differences to assess whether the new LLINs are superior. The primary comparison will occur at the end of the 24 months post intervention period. To adjust for the increased risk of a Type I error due to multiple pairwise comparisons, the level of significance will be adjusted using the Bonferroni method. Least square mean differences between each intervention arm versus control will be calculated at each time, together with 98.23% confidence intervals (reflecting adjustment of usual 95% CI to account for multiple comparisons). Secondary analyses will include all possible pairwise comparisons of Dual-AI LLIN arms.[62] Sub-group analyses will be performed

For peer review only - http://bmjopen.bmj.com/site/about/guidelines.xhtml

to investigate the impacts of interventions according to different individual (i.e. age, sex), household (i.e. wealth, distance to health facilities) and cluster-level (i.e. vector resistance intensity, vector species composition) characteristics.

EIR will be estimated as the mean number of sporozoite infected mosquitoes per house per night (by species and overall) and weighted to account for proportion of mosquitoes processed for sporozoites. Differences in *Anopheles* density and EIR between the different arms will be estimated using random effects negative binomial regression taking into account the intracluster correlation. Random effects logistic regression will be used to compare sporozoite rate between study arms.

Economic evaluation

Following relevant guidelines,[63 64] we will combine primary trial data with secondary data in a decision analytic model using a decision tree. We will adopt a societal perspective, presenting costs both combined and disaggregated by payer, and model effects and costs over a lifetime horizon. In the main analysis, we will assume that nets are distributed in mass campaigns every three years, reflecting both what was done in the trial and standard practice in malaria control.

Effects will be presented as DALYs (discounted at 3% and with no age weighting), calculated as the sum of years of life with disability from malaria-related illness (uncomplicated and severe malaria cases and anaemia) and years of life lost from malaria deaths.[63] The number of cases associated with each intervention will be modelled as the product of the incidence in the reference arm, the incidence rate ratio of the relevant arm to the reference arm, and a standardized population size. Incidence in children aged 6 months to 10 years will reflect ITT trial data, while incidence in other age groups, which comprise a small share of overall cases

For peer review only - http://bmjopen.bmj.com/site/about/guidelines.xhtml

and deaths, will be estimated as a function of the incidence in children based on publicly available modelling.[58 65] DALYs will be calculated as the product of the estimated number of malaria cases (as above), the case fatality rate, and the remaining life expectancy at age of death. Rates of progression to severe disease and to death will be estimated to reflect real-world outcomes based on secondary data. In the main analysis (based on trial data at 24 months), possible bounds for incidence rate ratios over months 25-36 will be estimated based on annual rate ratios over 0-12 and 13-24 months and assuming no relative effect compared to s-LLINs. In additional analyses, effects will also be presented as malaria cases and percentage point reduction in malaria prevalence.

We will plot the costs and effects of each intervention on the cost-effectiveness plane, identify the cost-effectiveness frontier and expansion path, and calculate incremental cost-effectiveness ratios (ICERs) between adjacent points on this path. Deterministic and probabilistic sensitivity analysis will explore the impact of uncertainty and heterogeneity on cost-effectiveness results. Cost-effectiveness acceptability curves[66] will indicate the probability of each strategy being the most cost-effective choice at plausible cost-effectiveness thresholds.[67 68] Affordability will be explored by comparing additional financial costs (net of cost savings) per person to relevant expenditure levels. Analyses will be conducted in Microsoft Excel with Visual Basic for Applications.

Secondary outcomes

Incidence

Cumulative incidence of malaria infection over the 24 months follow up (accounting for repeat episodes in the same child but measured on separate cohorts at 12 and 24 months post-

For peer review only - http://bmjopen.bmj.com/site/about/guidelines.xhtml

intervention) will be analysed using mixed effects Poisson or negative binomial regression. The model will include fixed effects for time, study arm, and time by study arm, and will adjust for the covariates used in the covariate-constrained allocation procedure. The model will account for within-period and between-period intra-cluster correlations. Least square mean differences will be obtained from the model to compare each intervention versus the control. Follow-up within 14 days of a previous episode in the same child will be censored and will not be included in the analysis. An offset for duration of follow-up will be included in the model to account for attrition.

Prevalence of anaemia

Prevalence of moderate and severe anaemia in children under 5 at 12,18, 24, 30 and 36 months will be analysed using mixed effects logistic regression similar to the approach outlined for malaria prevalence.

Insecticide resistance monitoring

Bioassay data will be interpreted according to the updated WHO guidelines: mortality of ≥98% indicates susceptibility at the diagnostic dose, mortality of 90-97% is suggestive of resistance, and mortality of less than 90% indicates resistance.[69] For resistance intensity assays at 5X insecticide concentrations, mortality ≥98% indicates low intensity resistance and mortality <98% indicates moderate to high intensity resistance. For resistance intensity assays at 10X insecticide concentrations, mortality ≥98% indicates moderate intensity resistance and mortality <98% indicates high intensity resistance.

For peer review only

For metabolic gene assays, relative expression level and Fold Change (FC) of each target gene from resistant and susceptible field samples, relative to the susceptible laboratory strain (*An. gambiae* s.s Kisumu or *An. funestus* s.s FANG), will be calculated using the $2^{-\Delta\Delta CT}$ method, incorporating PCR efficiency and normalised relative to the endogenous housekeeping control gene.[70]

Ethics approval

This protocol has been reviewed and approved by all institutional review boards, including: the Medical Research Coordinating Committee (MRCC) of the National Institute for Medical Research (NIMR), London School of Hygiene and Tropical Medicine (LSHTM), Kilimanjaro Christian Medical University College (KCMUCO); and University of Ottawa.

This study will be conducted according to the Declaration of Helsinki and the International Guidelines for Ethical Review of Epidemiological Studies. All field and clinical staff as well as the principal investigator will receive training on good clinical and laboratory practice before data collection begins, and refresher training every year. For all data collection activities (epidemiological and entomological), written informed consent will be obtained from an adult guardian in the household. The consent form will be written in Swahili and indicate the purpose of the study, the procedures, risks and benefits, that participation is completely voluntary, and that they may withdraw at any time with impunity. The study questionnaire will also be administered in Swahili.

Dissemination

For peer review only

For peer review only - http://bmjopen.bmj.com/site/about/guidelines.xhtml

Study findings will be shared in stakeholder meetings attended by local community leaders, the Ministry of Health, Community Development, Gender, Elderly and Children, the National Malaria Control Programme, the President's office regional administration and local government representatives. Results will also be shared through peer-reviewed publications, at scientific conferences, and through clinicaltrials.gov.

Figure Titles

Figure 1: Secondary objectives

Figure 2: Trial study design

Figure 3: Study area. Map showing Misungwi study area in Mwanza region, North Tanzania (panel A), the 85 study clusters identified with core and buffer area and intervention allocation (panel B). Panel C shows a closer map on the minimum 600 hundred meters area between houses in adjacent clusters.

For peer review only

For peer review only - http://bmjopen.bmj.com/site/about/guidelines.xhtml

References

1. WHO. World Malaria Report Geneva, Switzerland, 2018.

2. WHO. World malaria report. Geneva, Switzerland, 2019.

3. Asidi A, N'Guessan R, Akogbeto M, et al. Loss of household protection from use of insecticide-treated nets against pyrethroid-resistant mosquitoes, benin. *Emerg Infect Dis* 2012;18(7):1101-6. doi: 10.3201/eid1807.120218

4. Ochomo EO, Bayoh NM, Walker ED, et al. The efficacy of long-lasting nets with declining physical integrity may be compromised in areas with high levels of pyrethroid resistance. *Malar J* 2013;12:368. doi: 10.1186/1475-2875-12-368

5. Kleinschmidt I, Bradley J, Knox TB, et al. Implications of insecticide resistance for malaria vector control with long-lasting insecticidal nets: a WHO-coordinated, prospective, international, observational cohort study. *The Lancet infectious diseases* 2018;18(6):640-49. doi: 10.1016/S1473-3099(18)30172-5

6. Lindblade KA, Mwandama D, Mzilahowa T, et al. A cohort study of the effectiveness of insecticide-treated bed nets to prevent malaria in an area of moderate pyrethroid resistance, Malawi. *Malar J* 2015;14:31. doi: 10.1186/s12936-015-0554-1

7. Ochomo E, Chahilu M, Cook J, et al. Insecticide-Treated Nets and Protection against Insecticide-Resistant Malaria Vectors in Western Kenya. *Emerg Infect Dis* 2017;23(5):758-64. doi: 10.3201/eid2305.161315

8. Protopopoff N, Mosha JF, Lukole E, et al. Effectiveness of a long-lasting piperonyl butoxide-treated insecticidal net and indoor residual spray interventions, separately and together, against malaria transmitted by pyrethroid-resistant mosquitoes: a cluster, randomised controlled, two-by-two factorial design trial. *Lancet* 2018;391(10130):1577-88. doi: 10.1016/S0140-6736(18)30427-6

9. WHO. Report of the 12th WHOPES Working Group meeting. Geneva, Switzerland: World Health Organization, 2008:41-75.

10. WHO. Conditions for use of long-lasting insecticidal nets treated with a pyrethroid and piperonyl butoxide: World Health Organization, 2015.

11. WHO. Conditions for deployment of mosquito nets treated with a pyrethroid and piperonyl butoxide. Geneva: World Health Organization, 2017.

12. Staedke SG, Gonahasa S, Dorsey G, et al. Effect of long-lasting insecticidal nets with and without piperonyl butoxide on malaria indicators in Uganda (LLINEUP): a pragmatic, cluster-randomised trial embedded in a national LLIN distribution campaign. *Lancet* 2020;395(10232):1292-303. doi: 10.1016/S0140-6736(20)30214-2

13. N'Guessan R, Odjo A, Ngufor C, et al. A Chlorfenapyr Mixture Net Interceptor(R) G2 Shows High Efficacy and Wash Durability against Resistant Mosquitoes in West Africa. *PLoS One* 2016;11(11):e0165925. doi: 10.1371/journal.pone.0165925

For peer review only - http://bmjopen.bmj.com/site/about/guidelines.xhtml

14. Ngufor C, N'Guessan R, Fagbohoun J, et al. Efficacy of the Olyset Duo net against insecticide-resistant mosquito vectors of malaria. *Sci Transl Med* 2016;8(356):356ra121. doi: 10.1126/scitranslmed.aad3270

15. Djenontin A, Ahoua Alou LP, Koffi A, et al. Insecticidal and sterilizing effect of Olyset Duo(R), a permethrin and pyriproxyfen mixture net against pyrethroid-susceptible and -resistant strains of Anopheles gambiae s.s.: a release-recapture assay in experimental huts. *Parasite* 2015;22:27. doi: 10.1051/parasite/2015027

16. Koffi AA, Ahoua Alou LP, Djenontin A, et al. Efficacy of Olyset(R) Duo, a permethrin and pyriproxyfen mixture net against wild pyrethroid-resistant Anopheles gambiae s.s. from Cote d'Ivoire: an experimental hut trial. *Parasite* 2015;22:28. doi: 10.1051/parasite/2015028

17. Kawada H, Dida GO, Ohashi K, et al. A small-scale field trial of pyriproxyfen-impregnated bed nets against pyrethroid-resistant Anopheles gambiae s.s. in western Kenya. *PloS one* 2014;9(10):e111195. doi: 10.1371/journal.pone.0111195

18. Bayili K, N'do S, Namountougou M, et al. Evaluation of efficacy of Interceptor((R)) G2, a long-lasting insecticide net coated with a mixture of chlorfenapyr and alpha-cypermethrin, against pyrethroid resistant Anopheles gambiae s.l. in Burkina Faso. *Malaria journal* 2017;16(1):190. doi: 10.1186/s12936-017-1846-4

19. Camara S, Ahoua Alou LP, Koffi AA, et al. Efficacy of Interceptor((R)) G2, a new long-lasting insecticidal net against wild pyrethroid-resistant Anopheles gambiae s.s. from Cote d'Ivoire: a semi-field trial. *Parasite* 2018;25:42. doi: 10.1051/parasite/2018042

20. Ngufor C, Fagbohoun J, Critchley J, et al. Which intervention is better for malaria vector control: insecticide mixture long-lasting insecticidal nets or standard pyrethroid nets combined with indoor residual spraying? *Malaria journal* 2017;16(1):340. doi: 10.1186/s12936-017-1987-5

21. Ngufor C, Agbevo A, Fagbohoun J, et al. Efficacy of Royal Guard, a new alpha-cypermethrin and pyriproxyfen treated mosquito net, against pyrethroid-resistant malaria vectors. *Sci Rep* 2020;10(1):12227. doi: 10.1038/s41598-020-69109-5 [published Online First: 2020/07/24]

22. Tiono AB, Ouedraogo A, Ouattara D, et al. Efficacy of Olyset Duo, a bednet containing pyriproxyfen and permethrin, versus a permethrin-only net against clinical malaria in an area with highly pyrethroid-resistant vectors in rural Burkina Faso: a cluster-randomised controlled trial. *Lancet* 2018;392(10147):569-80. doi: 10.1016/S0140-6736(18)31711-2

23. WHO. Report of the 20th WHOPES Working Group meeting. Geneva WHO, 2017:4-46.

24. Tungu P, Kirby M, Malima R, et al. Interceptor(R) long-lasting insecticidal net: phase III evaluation over three years of household use and calibration with Phase II experimental hut outcomes. *Parasit Vectors* 2016;9:204. doi: 10.1186/s13071-016-1490-9

25. WHO. Design of epidemiological trials for vector control products: Report of a WHO expert advisory group. Geneva, Switzerland: WHO, 2017.

26. Kabula B, Tungu P, Matowo J, et al. Susceptibility status of malaria vectors to insecticides commonly used for malaria control in Tanzania. *Trop Med Int Health* 2012;17(6):742-50. doi: 10.1111/j.1365-3156.2012.02986.x

For peer review only - http://bmjopen.bmj.com/site/about/guidelines.xhtml

27. Kisinza WN, Nkya TE, Kabula B, et al. Multiple insecticide resistance in Anopheles gambiae from Tanzania: a major concern for malaria vector control. *Malar J* 2017;16(1):439. doi: 10.1186/s12936-017-2087-2

28. Matiya DJ, Philbert AB, Kidima W, et al. Dynamics and monitoring of insecticide resistance in malaria vectors across mainland Tanzania from 1997 to 2017: a systematic review. *Malar J* 2019;18(1):102. doi: 10.1186/s12936-019-2738-6 [published Online First: 2019/03/28]

29. NBS. National Bureau of Statistics, Demographic and Health Survey and Malaria Indicator Survey 2015-2016. Dar Es Salaam, Tanzania: National Bureau of Statistics, 2016.

30. Katureebe A, Zinszer K, Arinaitwe E, et al. Measures of Malaria Burden after Long-Lasting Insecticidal Net Distribution and Indoor Residual Spraying at Three Sites in Uganda: A Prospective Observational Study. *PLoS Med* 2016;13(11):e1002167. doi: 10.1371/journal.pmed.1002167

31. WHO. Guidelines for laboratory and field-testing of long-lasting insecticidal nets: World Health Organization, 2013.

32. Chan AW, Tetzlaff JM, Altman DG, et al. SPIRIT 2013 statement: defining standard protocol items for clinical trials. *Ann Intern Med* 2013;158(3):200-7. doi: 10.7326/0003-4819-158-3-201302050-00583 [published Online First: 2013/01/09]

33. National Bureau of Statistics N. The 2012 Population and Housing Census (PHC) for the United Republic of Tanzania Dar es salaam, Tanzania, 2012.

34. columbia.edu. Early Warning System Rainfall Estimate (RFE) 2020 [Available from: https://iridl.ldeo.columbia.edu/SOURCES/.NOAA/.NCEP/.CPC/.FEWS/.Africa/.DAILY/.RFEv2/.est_prcp.

35. Nkya TE, Mosha FW, Magesa SM, et al. Increased tolerance of Anopheles gambiae ss to chemical insecticides after exposure to agrochemical mixture. *Tanzania Journal of Health Research* 2014;16(4)

36. PMI. Tanzania malaria operational plan FY 2018. https://wwwpmigov/docs/default-source/default-document-library/malaria-operational-plans/fy-2018/fy-2018-tanzania-malaria-operational-plan, 2018.

37. Hayes RJ, Moulton LH. Chapter 6: Randomisation procedures. Cluster Randomised Trials. London: Chapman & Hall/CRC 2009:149-62.

38. Tanzania MoHaSW. National Malaria strategic plan 2014-2020, 2014.

39. Harris C, Lwetoijera DW, Dongus S, et al. Sterilising effects of pyriproxyfen on Anopheles arabiensis and its potential use in malaria control. *Parasit Vectors* 2013;6:144. doi: 10.1186/1756-3305-6-144

40. Lwetoijera D, Harris C, Kiware S, et al. Effective autodissemination of pyriproxyfen to breeding sites by the exophilic malaria vector Anopheles arabiensis in semi-field settings in Tanzania. *Malar J* 2014;13:161. doi: 10.1186/1475-2875-13-161

For peer review only - http://bmjopen.bmj.com/site/about/guidelines.xhtml

41. Raghavendra K, Barik TK, Sharma P, et al. Chlorfenapyr: a new insecticide with novel mode of action can control pyrethroid resistant malaria vectors. *Malaria journal* 2011;10:16. doi: 10.1186/1475-2875-10-16

42. Measure D, PMI, RBM, UNICEF, WHO. Household survey indicators for malaria control. Geneva: World Health Organization, 2018.

43. West PA, Protopopoff N, Wright A, et al. Enhanced protection against malaria by indoor residual spraying in addition to insecticide treated nets: is it dependent on transmission intensity or net usage? *PLoS One* 2015;10(3):e0115661. doi: 10.1371/journal.pone.0115661

44. Gillies MTC, M. A supplement to the Anophelinae of Africa south of the Sahara (Afrotropical region)1987.

45. Detinova T, Gillies M. Observations on the determination of the age composition and epidemiological importance of populations of Anopheles gambiae Giles and Anopheles funestus Giles in Tanganyika. *Bulletin of the World Health Organization* 1964;30(1):23.

46. Wirtz RA, Zavala F, Charoenvit Y, et al. Comparative testing of monoclonal antibodies against Plasmodium falciparum sporozoites for ELISA development. *Bull World Health Organ* 1987;65(1):39-45.

47. Durnez L, Van Bortel W, Denis L, et al. False positive circumsporozoite protein ELISA: a challenge for the estimation of the entomological inoculation rate of malaria and for vector incrimination. *Malaria Journal* 2011;10(1):195.

48. Bass C, Williamson MS, Field LM. Development of a multiplex real-time PCR assay for identification of members of the Anopheles gambiae species complex. *Acta Trop* 2008;107(1):50-3. doi: 10.1016/j.actatropica.2008.04.009 [published Online First: 2008/05/21]

49. Vezenegho SB, Bass C, Puinean M, et al. Development of multiplex real-time PCR assays for identification of members of the Anopheles funestus species group. *Malar J* 2009;8:282. doi: 10.1186/1475-2875-8-282

50. Bass C, Nikou D, Donnelly MJ, et al. Detection of knockdown resistance (kdr) mutations in Anopheles gambiae: a comparison of two new high-throughput assays with existing methods. *Malar J* 2007;6:111. doi: 10.1186/1475-2875-6-111

51. Brogdon WG, Chan A. Guideline for evaluating insecticide resistance in vectors using the CDC bottle bioassay. Altlanta, USA: Centers for Disease Control and Prevention, 2012.

52. WHO. Test procedures for insecticide resistance monitoring in malaria vector mosquitoes (Second edition). Geneva, Switzerland: World Health organization, 2016.

53. Matowo J, Kitau J, Kaaya R, et al. Trends in the selection of insecticide resistance in Anopheles gambiae s.l. mosquitoes in northwest Tanzania during a community randomized trial of longlasting insecticidal nets and indoor residual spraying. *Med Vet Entomol* 2015;29(1):51-9. doi: 10.1111/mve.12090

For peer review only

54. Mavridis K, Wipf N, Medves S, et al. Rapid multiplex gene expression assays for monitoring metabolic resistance in the major malaria vector Anopheles gambiae. *Parasites & vectors* 2019;12(1):9. doi: 10.1186/s13071-018-3253-2

55. Charlwood JD, Rowland M, Protopopoff N, et al. The Furvela tent-trap Mk 1.1 for the collection of outdoor biting mosquitoes. *PeerJ* 2017;5:e3848. doi: 10.7717/peerj.3848

56. Georgios Gkountouras JAL, Anderson Stanciole, Karin Stenberg, and Tessa Tan-Torres Edejer. Estimation of unit costs for general health services: Updated WHO-CHOICE estimates: WHO department of Health Systems Financing, 2011.

57. Organization WH. Life Tables. 2020

58. DALYs GBD, Collaborators H. Global, regional, and national disability-adjusted life-years (DALYs) for 359 diseases and injuries and healthy life expectancy (HALE) for 195 countries and territories, 1990-2017: a systematic analysis for the Global Burden of Disease Study 2017. *Lancet* 2018;392(10159):1859-922. doi: 10.1016/S0140-6736(18)32335-3

59. Hayes RJ, Bennett S. Simple sample size calculation for cluster-randomized trials. *Int J Epidemiol* 1999;28(2):319-26.

60. West PA, Protopopoff N, Wright A, et al. Indoor residual spraying in combination with insecticide-treated nets compared to insecticide-treated nets alone for protection against malaria: a cluster randomised trial in Tanzania. *PLoS Med* 2014;11(4):e1001630. doi: 10.1371/journal.pmed.1001630

61. Kasza J, Hemming K, Hooper R, et al. Impact of non-uniform correlation structure on sample size and power in multiple-period cluster randomised trials. *Stat Methods Med Res* 2019;28(3):703-16. doi: 10.1177/0962280217734981 [published Online First: 2017/10/14]

62. Proschan MA, Waclawiw MA. Practical guidelines for multiplicity adjustment in clinical trials. *Control Clin Trials* 2000;21(6):527-39.

63. Wilkinson T, Sculpher MJ, Claxton K, et al. The International Decision Support Initiative Reference Case for Economic Evaluation: An Aid to Thought. *Value in health : the journal of the International Society for Pharmacoeconomics and Outcomes Research* 2016;19(8):921-28. doi: 10.1016/j.jval.2016.04.015

64. Husereau D, Drummond M, Petrou S, et al. Consolidated Health Economic Evaluation Reporting Standards (CHEERS) statement. *Bmj* 2013;346:f1049. doi: 10.1136/bmj.f1049

65. Evaluation IfHMa. Global Burden of Disease Results Tool. Seattle, USA University of Washington, 2017.

66. Fenwick E, Claxton K, Sculpher M. Representing uncertainty: the role of cost-effectiveness acceptability curves. *Health Econ* 2001;10(8):779-87. [published Online First: 2001/12/18]

67. Ochalek J, Lomas J, Claxton K. Estimating health opportunity costs in low-income and middle-income countries: a novel approach and evidence from cross-country data. *BMJ Glob Health* 2018;3(6):e000964. doi: 10.1136/bmjgh-2018-000964 [published Online First: 2018/11/30]

For peer review only - http://bmjopen.bmj.com/site/about/guidelines.xhtml

68. Marseille E, Larson B, Kazi DS, et al. Thresholds for the cost-effectiveness of interventions: alternative approaches. *Bull World Health Organ* 2015;93(2):118-24. doi: 10.2471/BLT.14.138206

69. WHO. Test procedures for insecticide resistance monitoring in malaria vector mosquitoes. Geneva, Switzerland: World Health organisation, 2016.

70. Rao X, Huang X, Zhou Z, et al. An improvement of the 2^(-delta delta CT) method for quantitative real-time polymerase chain reaction data analysis. *Biostat Bioinforma Biomath* 2013;3(3):71-85. [published Online First: 2013/08/01]

For peer review only

Authors' contribution

JFM helped in the design of the study protocol, wrote the first draft of the manuscript and is coordinating site investigators. MAK, supported the research grant proposal, helped in the design of the study protocol, and substantially revised the manuscript. LAM, contributed to the study protocol and the writing of the manuscript. FWM and AM supported the research grant proposal and design of the study, facilitated the obtention of ethical approval and revised the manuscript. CP designed the cost-effectiveness component of the protocol and revised the manuscript. MT gave statistical inputs to the protocol and manuscript. NM and EL contributed to the study design and revised the manuscript. NP and MR conceptualised the study. MR and IK contributed to the protocol, revised the manuscript and provided technical support. NP designed and drafted the study protocol, contributed to the writing of the manuscript and is the principal investigator of the study. All authors read and approved the final version of the manuscript.

Funding statement

This study is funded under the global health trial a jointly funded initiative from the Department for International Development (Dfid), the Medical Research Council, Wellcome and Department of Health and Social Care (Grant Ref: MR/R006040/1).

The study third year follow up is supported by a grant from Bill and Melinda Gate foundation, through the Innovative Vector Control Consortium.

The London School of Hygiene and Tropical Medicine is the trial sponsor.

Competing interests

The authors declare that they have no competing interests.

For peer review only - http://bmjopen.bmj.com/site/about/guidelines.xhtml

Clinical

To assess the effectiveness of py-PPF-LLINs, py-CFP-LLINs and py-PBO-LLINs compared to s-LLINs:

- On malaria incidence in children from 6 months to 10 years over two years
- On prevalence of moderate and severe anaemia in children under 5 years old
- On malaria infection prevalence in children from 6 months to 14 years after three years post intervention
- To evaluate the comparative effectiveness of the 3 Dual-AI LLINs on malaria prevalence and malaria incidence

Entomological

To assess the effectiveness of py-PPF-LLINs, py-CFP-LLINs and py-PBO-LLINs compared to s-LLINs:

- On EIR of malaria vectors collected indoors three years post intervention
- On insecticide resistance mechanisms (target site mutations and metabolic enzyme expression) in *Anopheles gambiae* s.l. and *Anopheles funestus s.l.*
- On other entomological outcomes; 1) indoor and outdoor density, 2) feeding and resting preference, 3) parous rates and 4) ovary development and fecundity (only py-PPF-ITNs) in *Anopheles gambiae* s.l. and *Anopheles funestus s.l.*
- To evaluate the comparative effectiveness of the 3 Dual-AI LLINs on EIR

Economical

To assess the incremental budget impact of each of the Dual-AI LLINs relative to s-LLINs and one another

Secondary objectives

338x190mm (96 x 96 DPI)

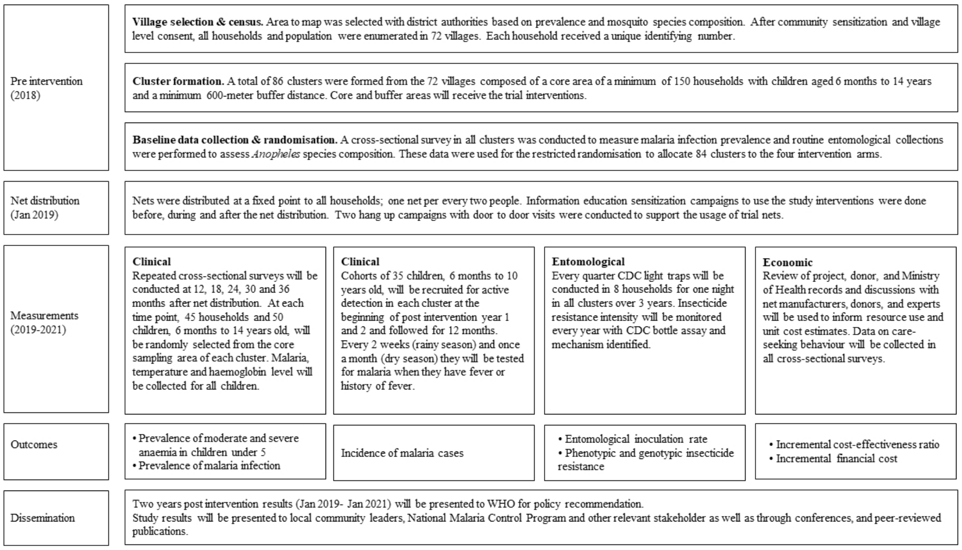

Trial study design

338x190mm (96 x 96 DPI)

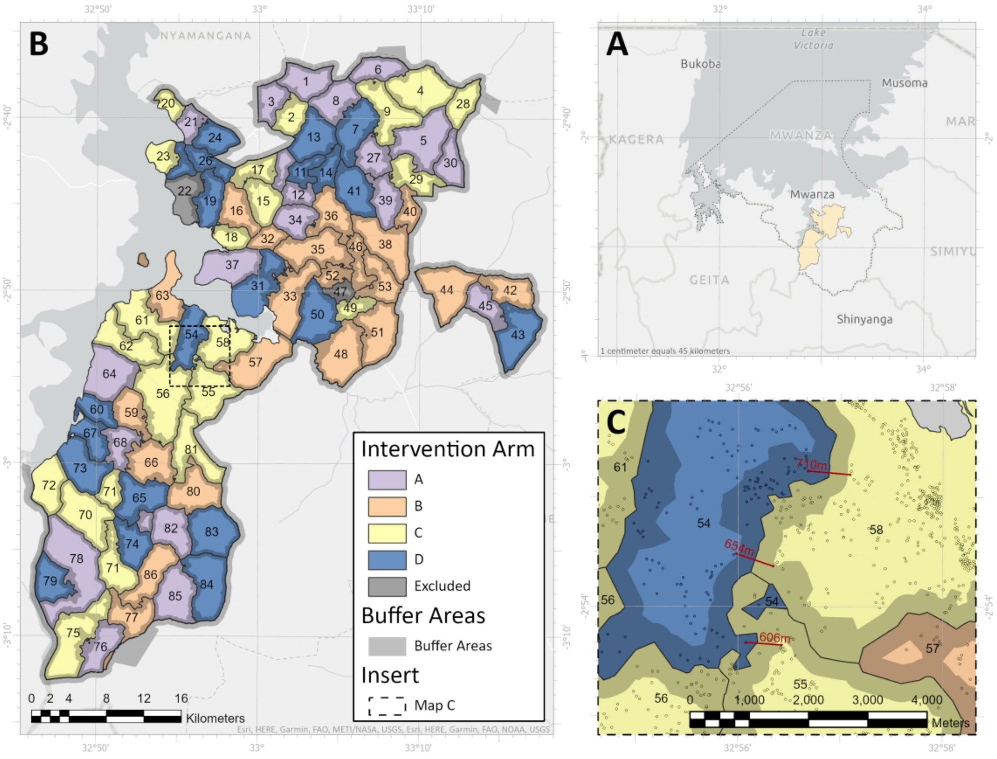

Study area. Map showing Misungwi study area in Mwanza region, North Tanzania (panel A), the 85 study clusters identified with core and buffer area and intervention allocation (panel B). Panel C shows a closer map on the minimum 600 hundred meters area between houses in adjacent clusters.

224x170mm (300 x 300 DPI)

Protocol for a four parallel-arm, single-blind, cluster-randomized trial to assess the effectiveness of three types of dual active ingredient treated nets compared to pyrethroid-only long-lasting insecticidal nets to prevent malaria transmitted by pyrethroid insecticide-resistant vector mosquitoes in Tanzania

Supplementary files

For peer review only

Methods

Ecological niche modelling

Suitability for each *Anopheles* species was determined using an ecological niche modelling approach, based on a maximum entropy (MaxEnt v.3.4.1) algorithm, similar to the approach described by Kulkarni et al. [1]. MaxEnt uses presence-only occurrence data in conjunction with environmental data to predict the potential distribution of a species across a defined landscape. Models were constructed for each major local species (*An. gambiae* s.s., *An. arabiensis* and *An. funestus* s.l.) using occurrence records from pilot mosquito monitoring alongside bioclimatic variables at a 1-km resolution from the WorldClim database (www.worldclim.org), including: temperature seasonality, annual precipitation, precipitation of wettest month, precipitation of driest month, and precipitation seasonality; and elevation from the Shuttle Radar Topography Mission (SRTM). To discriminate between sibling species of the An. gambiae s.l. complex, point locations where only An. gambiae s.s. (i.e. no An. arabiensis) was detected (n=12) were used as occurrence records. Point locations where >50% of the Anopheles mosquitoes were identified as An. funestus s.l. (n=51) were used as occurrence records for this species. Data were randomly partitioned for model evaluation, with 75% of the records used as training data to construct the models and the remaining 25% set aside for testing. The accuracy of each model was determined by performing both a threshold-dependent binomial test of omission and a threshold-independent receiver operating characteristic analysis; models with an area under the curve (AUC) >0.8 were retained, and the model with the highest AUC value for each species was used for subsequent analysis. The mean probability of suitability for each cluster was calculated using zonal statistics in ArcGIS.

Entomological data collection

Routine entomology collection

Cross-sectional entomological surveys will be carried out in 84 study clusters to monitor the indoor mosquito population density. Each cluster will be visited once every quarter; each month, 7 clusters from each study arm will be selected. Indoor mosquito densities will be monitored using Centers for Disease Control and Prevention (CDC) Miniature light traps (John W Hock Company, USA) in 8 randomly selected households in each of the cluster core areas. During the collection night, the owner's bed net in the bed/sleeping place where the CDC light trap will be installed will be substituted with a project standard LLIN, and replaced the following day. For each of the selected houses, a short questionnaire will be administered to collect information about the number of inhabitants, type of house construction materials, presence of animals, where the animals are kept, coverage and usage of study nets and other net types found, and other malaria prevention measures used by household members. Sampled mosquitoes will be identified morphologically following the identification key by Gillies and Coetzee [2]. Physiological status of *Anopheles* mosquitoes (blood-fed, unfed, gravid, semi-gravid) will be recorded. Parity rates will be estimated in a subset of live *Anopheles* mosquitoes through dissection [3]. Maximum 10 *An. gambiae* s.l and 10 *An. funestus* s.l will be randomly picked per household per collection night and preserved for molecular analysis. The mosquito specimens will be screened for *Plasmodium falciparum* circumsporozoite protein (Pf-CSP) by ELISA [4]. The CSP-ELISA positive samples will be re-analysed by heating the ELISA lysates to remove any false positives [5].

All specimens positive for Pf-CSP, as well as a subsample of CSP negative of *An. gambiae* s.l. and *An. funestus* s.l from each cluster per survey round, will be analysed for sibling species identification. PCR TaqMan assays will be used to discriminate members of *An. gambiae* species complex [6], and members of the *An. funestus* group [7]. A subsample of *An. gambiae* s.s. and *An. arabiensis* that are screened for malaria sporozoites infection will be genotyped for the L1014F-*kdr* and L1014S-*kdr* mutations, associated with pyrethroid and DDT resistance, using TaqMan PCR assays, following the protocol by Bass *et al* [8].

Insecticide resistance monitoring

Phenotypic resistance and resistance levels to alpha-cypermethrin, permethrin, and chlorfenapyr, will be characterized at baseline and yearly post-intervention, in all four study arms, using WHO cylinder and modified CDC bottle bioassays [9, 10]. The PBO synergist effect on wild female *An. gambiae* s.l. and *An. funestus* s.l. will be assessed using pre-exposure of PBO in CDC bottles followed by permethrin resistance intensity assays. The effect of PPF on fertility/egg development in adult female *Anopheles*, after 72 hours post-exposure to PPF using CDC bottle bioassays, will be assessed by ovarian dissection under a light microscope [11]. The developmental status of ovarian follicles/eggs will be classified as fertile or infertile and interpreted following the Christopher's stages of egg development [12, 13]. All assays will be performed using wild, indoor resting mosquitoes collected from house walls using

For peer review only

Prokopack and manual aspirators. Mosquitoes will be held for three days, fed on 10% glucose solution, to allow for blood-meal digestion, prior to bioassay testing. Mosquitoes will be morphologically identified as *An. gambiae* s.l. or *An. funestus* s.l. [2], and species complexes will be tested separately [6, 7]. All knockdown/dead mosquitoes at 60 minutes and surviving 72 hours post-exposure to insecticides will be stored individually in RNAlater® and preserved at -20°C for gene expression analysis. Per mosquito, 4-6 legs will be removed to identify individuals to species-level using TaqMan PCR [34-36]; only PCR-confirmed *An. gambiae* s.s. or *An. funestus* s.s., will be pooled separately for further analysis. RNA will be extracted from pools of *An. gambiae* s.s. or *An. funestus* s.s and cDNA synthesized, according to standard procedures. Relative expression of CYPs and other metabolic enzymes, previously identified as being over-expressed in resistant *An. gambiae* s.s. and *An. funestus* s.s. populations in Tanzania [14], will be measured using multiplex TaqMan RT-qPCR assays [15].

Mosquito behaviour

Malaria vector abundance, species composition, feeding and resting behaviours (including biting time, host preference), and contribution to outdoor malaria transmission will be assessed at baseline and after implementation of the interventions to assess changes over time. These mosquito behavioural adaptations will be assessed twice a year during the high mosquito density seasons (April-May and October-November)using CDC light traps indoor, occupied adapted Furvela tent traps outdoors [16] and indoor and outdoor resting collection using Prokopack aspirators. Collections will be done in 2 houses in 40 sentinel clusters (10 clusters per treatment arm) selected for their had high density of mosquitoes. In each household, light traps will be set up inside and one tent installed outside, close to the house. In the house were the mosquito traps will be installed, indoor resting mosquito collections will be conducted on the walls, roofs, and inside the nets. Similarly, outdoor collections of resting mosquitoes will be performed in potential resting sites around the house, such as open resting structures, cow sheds and pit latrines. A sub-sample of An. gambiae s.l. and An. funestus s.l. per each collection method will be tested for the presence of Pf-CSP [4] and identified to sibling species level [6, 7]. All freshly collected blood-fed Anopheles mosquitoes will be screened for the blood meal sources[17] .

The sterilizing effect of py-PPF ITN (Royal Guard®) relative to the standard LLINs on egg development in female wild malaria vectors will be assessed. This study will be conducted once a year in 10 sentinel clusters receiving Royal Guard, and 10 sentinel clusters receiving the standard LLIN. 10 houses per cluster will be visited, and sampled for freshly blood fed mosquitoes resting on the wall or inside the nets. Anopheles will be supplied with 10% glucose solution, monitored for survival and dissected 72 hours after collection to look at the developmental stages of eggs as a proxy of fertility [11]. Gravid alive females will be anesthetized at −20°C for 8–10 min and individually dissected in a drop of distilled water by gently pulling out the last two segments, 7th and 8th sternites of the mosquito abdomen.

For peer review only

References

1. Kulkarni MA, Desrochers RE, Kerr JT: **High resolution niche models of malaria vectors in northern Tanzania: a new capacity to predict malaria risk?** *PLoS One* 2010, **5:**e9396.

2. Gillies MTC, M.: *A supplement to the Anophelinae of Africa south of the Sahara (Afrotropical region).* 1987.

3. Detinova T, Gillies M: **Observations on the determination of the age composition and epidemiological importance of populations of Anopheles gambiae Giles and Anopheles funestus Giles in Tanganyika.** *Bulletin of the World Health Organization* 1964, **30:**23.

4. Wirtz RA, Zavala F, Charoenvit Y, Campbell GH, Burkot TR, Schneider I, Esser KM, Beaudoin RL, Andre RG: **Comparative testing of monoclonal antibodies against Plasmodium falciparum sporozoites for ELISA development.** *Bull World Health Organ* 1987, **65:**39-45.

5. Durnez L, Van Bortel W, Denis L, Roelants P, Veracx A, Trung HD, Sochantha T, Coosemans M: **False positive circumsporozoite protein ELISA: a challenge for the estimation of the entomological inoculation rate of malaria and for vector incrimination.** *Malaria Journal* 2011, **10:**195.

6. Bass C, Williamson MS, Field LM: **Development of a multiplex real-time PCR assay for identification of members of the Anopheles gambiae species complex.** *Acta tropica* 2008, **107:**50-53.

7. Vezenegho SB, Bass C, Puinean M, Williamson MS, Field LM, Coetzee M, Koekemoer LL: **Development of multiplex real-time PCR assays for identification of members of the Anopheles funestus species group.** *Malaria Journal* 2009, **8:**1-9.

8. Bass C, Nikou D, Donnelly MJ, Williamson MS, Ranson H, Ball A, Vontas J, Field LM: **Detection of knockdown resistance (kdr) mutations in Anopheles gambiae: a comparison of two new high-throughput assays with existing methods.** *Malaria Journal* 2007, **6:**1-14.

9. Brogdon WG, Chan A: **Guideline for evaluating insecticide resistance in vectors using the CDC bottle bioassay.** Altlanta, USA: Centers for Disease Control and Prevention; 2012.

10. WHO: **Test procedures for insecticide resistance monitoring in malaria vector mosquitoes (Second edition).** Geneva, Switzerland: World Health organization; 2016.

11. Koama B, Namountougou M, Sanou R, Ndo S, Ouattara A, Dabire RK, Malone D, Diabate A: **The sterilizing effect of pyriproxyfen on the malaria vector Anopheles gambiae: physiological impact on ovaries development.** *Malar J* 2015, **14:**101.

12. Christophers SR: **The development of the egg follicles in anophelines.** *Paludism* 1911, **2:**73-88.

13. Koama B, Namountougou M, Sanou R, Ndo S, Ouattara A, Dabiré RK, Malone D, Diabaté A: **The sterilizing effect of pyriproxyfen on the malaria vector Anopheles gambiae: physiological impact on ovaries development.** *Malaria journal* 2015, **14:**101.

For peer review only

14. Matowo J, Kitau J, Kaaya R, Kavishe R, Wright A, Kisinza W, Kleinschmidt I, Mosha F, Rowland M, Protopopoff N: **Trends in the selection of insecticide resistance in Anopheles gambiae s.l. mosquitoes in northwest Tanzania during a community randomized trial of longlasting insecticidal nets and indoor residual spraying.** *Med Vet Entomol* 2015, **29:**51-59.

15. Mavridis K, Wipf N, Medves S, Erquiaga I, Muller P, Vontas J: **Rapid multiplex gene expression assays for monitoring metabolic resistance in the major malaria vector Anopheles gambiae.** *Parasit Vectors* 2019, **12:**9.

16. Charlwood JD, Rowland M, Protopopoff N, Le Clair C: **The Furvela tent-trap Mk 1.1 for the collection of outdoor biting mosquitoes.** *PeerJ* 2017, **5:**e3848.

17. Beier JC, Perkins PV, Wirtz RA, Koros J, Diggs D, Gargan TP, Koech DK: **Bloodmeal identification by direct enzyme-linked immunosorbent assay (ELISA), tested on Anopheles (Diptera: Culicidae) in Kenya.** *Journal of medical entomology* 1988, **25:**9-16.

For peer review only

SPIRIT — STANDARD PROTOCOL ITEMS: RECOMMENDATIONS FOR INTERVENTIONAL TRIALS

SPIRIT 2013 Checklist: Recommended items to address in a clinical trial protocol and related documents*

Section/item	Item No	Description	Addressed on page number
**Administrative information**			
Title	1	Descriptive title identifying the study design, population, interventions, and, if applicable, trial acronym	___1
Trial registration	2a	Trial identifier and registry name. If not yet registered, name of intended registry	___1
	2b	All items from the World Health Organization Trial Registration Data Set	___NA
Protocol version	3	Date and version identifier	in supplementary file (protocol)
Funding	4	Sources and types of financial, material, and other support	___36
Roles and responsibilities	5a	Names, affiliations, and roles of protocol contributors	___1
	5b	Name and contact information for the trial sponsor	___36
	5c	Role of study sponsor and funders, if any, in study design; collection, management, analysis, and interpretation of data; writing of the report; and the decision to submit the report for publication, including whether they will have ultimate authority over any of these activities	in supplementary file (protocol)
	5d	Composition, roles, and responsibilities of the coordinating centre, steering committee, endpoint adjudication committee, data management team, and other individuals or groups overseeing the trial, if applicable (see Item 21a for data monitoring committee)	in supplementary file (protocol)

For peer review only

**Introduction**			
Background and rationale	6a	Description of research question and justification for undertaking the trial, including summary of relevant studies (published and unpublished) examining benefits and harms for each intervention	__5 - 8
	6b	Explanation for choice of comparators	__12 - 13
Objectives	7	Specific objectives or hypotheses	__8
Trial design	8	Description of trial design including type of trial (eg, parallel group, crossover, factorial, single group), allocation ratio, and framework (eg, superiority, equivalence, noninferiority, exploratory)	__10
**Methods: Participants, interventions, and outcomes**			
Study setting	9	Description of study settings (eg, community clinic, academic hospital) and list of countries where data will be collected. Reference to where list of study sites can be obtained	__8 - 9
Eligibility criteria	10	Inclusion and exclusion criteria for participants. If applicable, eligibility criteria for study centres and individuals who will perform the interventions (eg, surgeons, psychotherapists)	__15
Interventions	11a	Interventions for each group with sufficient detail to allow replication, including how and when they will be administered	__12 - 13
	11b	Criteria for discontinuing or modifying allocated interventions for a given trial participant (eg, drug dose change in response to harms, participant request, or improving/worsening disease)	__NA
	11c	Strategies to improve adherence to intervention protocols, and any procedures for monitoring adherence (eg, drug tablet return, laboratory tests)	__12 - 13
	11d	Relevant concomitant care and interventions that are permitted or prohibited during the trial	__17
Outcomes	12	Primary, secondary, and other outcomes, including the specific measurement variable (eg, systolic blood pressure), analysis metric (eg, change from baseline, final value, time to event), method of aggregation (eg, median, proportion), and time point for each outcome. Explanation of the clinical relevance of chosen efficacy and harm outcomes is strongly recommended	___14
Participant timeline	13	Time schedule of enrolment, interventions (including any run-ins and washouts), assessments, and visits for participants. A schematic diagram is highly recommended (see Figure)	in supplementary file (protocol)

For peer review only - http://bmjopen.bmj.com/site/about/guidelines.xhtml

Sample size	14	Estimated number of participants needed to achieve study objectives and how it was determined, including clinical and statistical assumptions supporting any sample size calculations	___21 - 22
Recruitment	15	Strategies for achieving adequate participant enrolment to reach target sample size	___21 - 22
**Methods: Assignment of interventions (for controlled trials)**			
Allocation:			
Sequence generation	16a	Method of generating the allocation sequence (eg, computer-generated random numbers), and list of any factors for stratification. To reduce predictability of a random sequence, details of any planned restriction (eg, blocking) should be provided in a separate document that is unavailable to those who enrol participants or assign interventions	___11 - 12
Allocation concealment mechanism	16b	Mechanism of implementing the allocation sequence (eg, central telephone; sequentially numbered, opaque, sealed envelopes), describing any steps to conceal the sequence until interventions are assigned	___12
Implementation	16c	Who will generate the allocation sequence, who will enrol participants, and who will assign participants to interventions	___12
Blinding (masking)	17a	Who will be blinded after assignment to interventions (eg, trial participants, care providers, outcome assessors, data analysts), and how	___10
	17b	If blinded, circumstances under which unblinding is permissible, and procedure for revealing a participant's allocated intervention during the trial	___10
**Methods: Data collection, management, and analysis**			
Data collection methods	18a	Plans for assessment and collection of outcome, baseline, and other trial data, including any related processes to promote data quality (eg, duplicate measurements, training of assessors) and a description of study instruments (eg, questionnaires, laboratory tests) along with their reliability and validity, if known. Reference to where data collection forms can be found, if not in the protocol	___15 - 20
	18b	Plans to promote participant retention and complete follow-up, including list of any outcome data to be collected for participants who discontinue or deviate from intervention protocols	___15 - 20

Data management	19	Plans for data entry, coding, security, and storage, including any related processes to promote data quality (eg, double data entry; range checks for data values). Reference to where details of data management procedures can be found, if not in the protocol	___22 - 23
Statistical methods	20a	Statistical methods for analysing primary and secondary outcomes. Reference to where other details of the statistical analysis plan can be found, if not in the protocol	___23 - 27
	20b	Methods for any additional analyses (eg, subgroup and adjusted analyses)	___23 - 24
	20c	Definition of analysis population relating to protocol non-adherence (eg, as randomised analysis), and any statistical methods to handle missing data (eg, multiple imputation)	in supplementary file (protocol)
**Methods: Monitoring**			
Data monitoring	21a	Composition of data monitoring committee (DMC); summary of its role and reporting structure; statement of whether it is independent from the sponsor and competing interests; and reference to where further details about its charter can be found, if not in the protocol. Alternatively, an explanation of why a DMC is not needed	in supplementary file (protocol)
	21b	Description of any interim analyses and stopping guidelines, including who will have access to these interim results and make the final decision to terminate the trial	in supplementary file (protocol)
Harms	22	Plans for collecting, assessing, reporting, and managing solicited and spontaneously reported adverse events and other unintended effects of trial interventions or trial conduct	in supplementary file (protocol)
Auditing	23	Frequency and procedures for auditing trial conduct, if any, and whether the process will be independent from investigators and the sponsor	in supplementary file (protocol)
**Ethics and dissemination**			
Research ethics approval	24	Plans for seeking research ethics committee/institutional review board (REC/IRB) approval	__35
Protocol amendments	25	Plans for communicating important protocol modifications (eg, changes to eligibility criteria, outcomes, analyses) to relevant parties (eg, investigators, REC/IRBs, trial participants, trial registries, journals, regulators)	in supplementary file (protocol)

Consent or assent	26a	Who will obtain informed consent or assent from potential trial participants or authorised surrogates, and how (see Item 32)	in supplementary file (protocol)
	26b	Additional consent provisions for collection and use of participant data and biological specimens in ancillary studies, if applicable	in supplementary file (protocol)
Confidentiality	27	How personal information about potential and enrolled participants will be collected, shared, and maintained in order to protect confidentiality before, during, and after the trial	___22 - 23
Declaration of interests	28	Financial and other competing interests for principal investigators for the overall trial and each study site	___35
Access to data	29	Statement of who will have access to the final trial dataset, and disclosure of contractual agreements that limit such access for investigators	in supplementary file (protocol)
Ancillary and post-trial care	30	Provisions, if any, for ancillary and post-trial care, and for compensation to those who suffer harm from trial participation	in supplementary file (protocol)
Dissemination policy	31a	Plans for investigators and sponsor to communicate trial results to participants, healthcare professionals, the public, and other relevant groups (eg, via publication, reporting in results databases, or other data sharing arrangements), including any publication restrictions	__3 & 28
	31b	Authorship eligibility guidelines and any intended use of professional writers	__35
	31c	Plans, if any, for granting public access to the full protocol, participant-level dataset, and statistical code	in supplementary file (protocol)
**Appendices**			
Informed consent materials	32	Model consent form and other related documentation given to participants and authorised surrogates	in supplementary file (protocol)
Biological specimens	33	Plans for collection, laboratory evaluation, and storage of biological specimens for genetic or molecular analysis in the current trial and for future use in ancillary studies, if applicable	in supplementary file (protocol)

*It is strongly recommended that this checklist be read in conjunction with the SPIRIT 2013 Explanation & Elaboration for important clarification on the items. Amendments to the protocol should be tracked and dated. The SPIRIT checklist is copyrighted by the SPIRIT Group under the Creative Commons "Attribution-NonCommercial-NoDerivs 3.0 Unported" license.

For peer review only - http://bmjopen.bmj.com/site/about/guidelines.xhtml

BMJ Open

Protocol for a four parallel-arm, single-blind, cluster-randomized trial to assess the effectiveness of three types of dual active ingredient treated nets compared to pyrethroid-only long-lasting insecticidal nets to prevent malaria transmitted by pyrethroid insecticide-resistant vector mosquitoes in Tanzania

Journal:	*BMJ Open*
Manuscript ID	bmjopen-2020-046664.R1
Article Type:	Protocol
Date Submitted by the Author:	21-Jan-2021

Complete List of Authors:	Mosha, Jacklin; National Institute for Medical Research Mwanza Research Centre,<br>Kulkarni, Manisha; University of Ottawa Faculty of Medicine, Epidemiology & Public Health<br>Messenger, Louisa; London School of Hygiene & Tropical Medicine, Disease Control<br>Rowland, Mark; London School of Hygiene & Tropical Medicine, Disease Control<br>Matowo, Nancy; London School of Hygiene & Tropical Medicine, Disease Control<br>Pitt, Catherine; London School of Hygiene & Tropical Medicine, Global Health and Development<br>Lukole, Eliud; National Institute for Medical Research Mwanza Research Centre; London School of Hygiene & Tropical Medicine, Disease Control<br>Taljaard, Monica; Ottawa Hospital Research Institute, Clinical Epidemiology Program<br>Thickstun, Charles; University of Ottawa Faculty of Medicine, Epidemiology & Public Health<br>Manjurano, Alphaxard; National Institute for Medical Research Mwanza Research Centre<br>Mosha, Franklin; Kilimanjaro Christian Medical University College<br>Kleinschmidt, Immo; London School of Hygiene & Tropical Medicine<br>Protopopoff, Natacha; London School of Hygiene & Tropical Medicine, Disease Control

**Primary Subject Heading**:	Epidemiology
Secondary Subject Heading:	Epidemiology, Global health, Health economics, Infectious diseases
Keywords:	PARASITOLOGY, INFECTIOUS DISEASES, Infection control < INFECTIOUS DISEASES, Epidemiology < INFECTIOUS DISEASES, HEALTH ECONOMICS, Public health < INFECTIOUS DISEASES

I, the Submitting Author has the right to grant and does grant on behalf of all authors of the Work (as defined in the below author licence), an exclusive licence and/or a non-exclusive licence for contributions from authors who are: i) UK Crown employees; ii) where BMJ has agreed a CC-BY licence shall apply, and/or iii) in accordance with the terms applicable for US Federal Government officers or employees acting as part of their official duties; on a worldwide, perpetual, irrevocable, royalty-free basis to BMJ Publishing Group Ltd ("BMJ") its licensees and where the relevant Journal is co-owned by BMJ to the co-owners of the Journal, to publish the Work in this journal and any other BMJ products and to exploit all rights, as set out in our licence.

The Submitting Author accepts and understands that any supply made under these terms is made by BMJ to the Submitting Author unless you are acting as an employee on behalf of your employer or a postgraduate student of an affiliated institution which is paying any applicable article publishing charge ("APC") for Open Access articles. Where the Submitting Author wishes to make the Work available on an Open Access basis (and intends to pay the relevant APC), the terms of reuse of such Open Access shall be governed by a Creative Commons licence – details of these licences and which Creative Commons licence will apply to this Work are set out in our licence referred to above.

Other than as permitted in any relevant BMJ Author's Self Archiving Policies, I confirm this Work has not been accepted for publication elsewhere, is not being considered for publication elsewhere and does not duplicate material already published. I confirm all authors consent to publication of this Work and authorise the granting of this licence.

For peer review only

Protocol for a four parallel-arm, single-blind, cluster-randomized trial to assess the effectiveness of three types of dual active ingredient treated nets compared to pyrethroid-only long-lasting insecticidal nets to prevent malaria transmitted by pyrethroid insecticide-resistant vector mosquitoes in Tanzania

Jacklin F. Mosha[1]*, Manisha A. Kulkarni[2], Louisa A. Messenger[3], Mark Rowland[3], Nancy Matowo[3], Catherine Pitt[4], Eliud Lukole[1], Monica Taljaard[2,5], Charles Thickstun[2], Alphaxard Manjurano[1], Franklin W Mosha[6], Immo Kleinschmidt[7,8,9], Natacha Protopopoff[3]

1. National Institute for Medical Research, Mwanza Medical Research Centre, Mwanza, Tanzania
2. School of Epidemiology and Public Health, University of Ottawa, Ottawa, Canada
3. Department of Disease Control, London School of Hygiene and Tropical Medicine, London, United Kingdom
4. Department of Global Health and Development, London School of Hygiene and Tropical Medicine, London, United Kingdom
5. Ottawa Hospital Research Institute, Ottawa, Canada
6. Kilimanjaro Christian Medical University College, Moshi, Tanzania
7. MRC Tropical Epidemiology Group, Department of Infectious Disease Epidemiology, London School of Hygiene and Tropical Medicine, London, United Kingdom

For peer review only

8. Wits Research Institute for Malaria, School of Pathology, Faculty of Health Sciences, University of the Witwatersrand, Johannesburg, South Africa.
9. Southern African Development Community Malaria Elimination Eight Secretariat, Windhoek, Namibia.

* Corresponding Author

Email: jfmosha@yahoo.com

Tel: +255 788 033 605

Word count: 5794

Funding: The Department of Health and Social Care, the Department for International Development, the Medical Research Council and the Wellcome Trust

Abstract

Introduction: The massive scale-up of long-lasting insecticidal nets (LLINs) has led to major reductions in malaria burden in many sub-Saharan African countries. This progress is threatened by widespread insecticide resistance among malaria vectors. This cluster randomized controlled trial (c-RCT) compares three of the most promising dual active ingredients LLINs (Dual AI LLINs), which incorporate mixtures of insecticides or insecticide synergists to standard LLINs in an area of pyrethroid insecticide resistance.

For peer review only

Methods: A four-arm, single-blinded, c-RCT will evaluate the effectiveness of three types of Dual-AI LLINs 1) Royal Guard®, combining two insecticides, pyriproxyfen (PPF) and the pyrethroid alpha-cypermethrin; 2) Interceptor® G2, combining chlorfenapyr and alpha-cypermethrin3) Olyset® Plus, a LLIN combining a synergist, piperonyl butoxide (PBO) and the pyrethroid permethrin, compared to 4) Interceptor® LN, a standard LLIN containing the pyrethroid alpha-cypermethrin as the sole AI. The primary outcomes are malaria infection prevalence in children aged 6 months to 14 years and entomological inoculation rate (EIR), as a standard measure of malaria transmission at 24 months post intervention and cost-effectiveness.

Ethics and dissemination: Ethical approval was received from the institutional review boards of the Tanzanian National Institute for Medical Research, Kilimanjaro Christian Medical University College, London School of Hygiene and Tropical Medicine, and University of Ottawa. Study findings will be actively disseminated via reports and presentations to stakeholders, local community leaders, and relevant national and international policy makers as well as through conferences, and peer-reviewed publications.

Trial registration number: NCT03554616

For peer review only - http://bmjopen.bmj.com/site/about/guidelines.xhtml

Strengths and limitations of this study

- This study is the first RCT to evaluate and compare the effectiveness of the next generation of dual-treated LLINs, Royal Guard® (a pyrethoid-pyriproxifen LLIN) and Interceptor® G2 (a pyrethroid-chlorfenapyr LLIN) against standard LLIN to prevent malaria infection prevalence and incidence in an area of pyrethroid insecticide resistance.
- Results of this study will be presented to the World Health Organization for policy recommendations and depending on the study outcomes has the potential to shape malaria vector control strategies across sub-Saharan Africa for the next decade.
- This is the second RCT to evaluate the new class of pyrethroid-PBO LLINs (Olyset® Plus) in the Great Lakes Zone of Tanzania, expanding the evidence basis for deployment of this intervention class in areas of pyrethroid resistance.
- The trialling of the three main categories of Dual-AI LLN against the standard pyrethroid LLIN in the same human cultural community against the same vector complex should offer the best opportunity to unravel relative effectiveness on malaria and effect on selection of insecticide resistance.
- Some limitations include the size of the cluster buffer areas, which might not prevent all contamination between intervention arms, and the use of malaria rapid diagnostic tests to assess malaria infections rather than double-read blood slides, which may be more sensitive.

For peer review only

Introduction

Long-lasting insecticidal nets (LLINs) are the primary method of malaria control in Sub-Saharan Africa. The World Health Organization (WHO) estimates that over 50% of the population of Sub-Saharan Africa now sleep under LLINs.[1] Together with improved diagnosis and treatment, LLINs have helped reduce malaria incidence by 42% and mortality by 66% in Africa in the last 15 years.[1] Pyrethroids are the only class of insecticide used routinely on LLINs, so the rapid spread of pyrethroid resistance across vector populations threatens to reverse the successes achieved so far, and may be a factor contributing to the current stagnation in malaria disease burden.[2]

Several studies have demonstrated that LLINs are becoming less effective at killing mosquitoes in areas of moderate to high pyrethroid resistance compared to those with susceptible vector populations.[3-8] The search for new insecticides suitable for LLIN treatment is vital to sustain effective malaria vector control.

During the last decade, the WHO has encouraged manufacturers to develop new types of bed nets to control resistant mosquitoes. The chemical industry responded initially by developing a Dual AI LLIN, combining a pyrethroid insecticide with the synergist piperonyl butoxide (PBO), which inhibits cytochrome P450 oxidases responsible for pyrethroid resistance. Pyrethroid-PBO LLINs (py-PBO LLINs) have been available since 2009,[9] but had only been recommended by WHO for limited deployment until[10] a recent cluster-randomized controlled trial (RCT) demonstrated a 44% reduction in malaria infection prevalence in children in the py-PBO LLIN arm (Olyset™ Plus) compared to the standard pyrethroid LLIN (s-LLIN) after two years.[8] In 2017, based on this study, the WHO recognised the public health value of this

For peer review only - http://bmjopen.bmj.com/site/about/guidelines.xhtml

new class of LLIN and recommended their scale up in area with pyrethroid resistance.[11] A second large-scale RCT in Uganda confirmed the superior protection of py-PBO LLIN against malaria compared to s-LLINs.[12]

Other manufacturers have responded to the WHO call by producing a different kind of Dual-AI LLIN that incorporates a mixture of insecticides from different insecticide classes. Mixtures of two insecticides on the same LLIN with differing modes of action have the potential to delay the evolution of resistance and extend the lifespan of both active ingredients on the LLIN. The two latest products are a pyrethroid-pyriproxyfen LLIN (py-PPF LLIN: Olyset® Duo and Royal Guard®) and a pyrethroid-chlorfenapyr LLIN (py-CFP LLIN: Interceptor® G2). Both types of product have shown superior efficacy compared to s-LLINs in small-scale experimental hut trials.[13-20] The Py-PPF LLINs have demonstrated enhanced efficacy vector oviposition suppression and up to 95% reduction in vector reproductive rate in Benin.[14 21] In addition, Py-PPF LLIN Olyset® Duo, when compared with s-LLINs in an RCT in Burkina Faso, had a significantly greater impact on clinical malaria.[22] The py-CFP LLIN, when compared to s-LLINs, has shown enhanced efficacy through higher killing against resistant mosquito species in experimental hut trials, producing mortalities with *An. gambiae* s.l. of 71% *vs*. 20% in Benin[20] and 78% *vs*. 17% in Burkina Faso,[18] and 71% *vs*. 45% with *An. arabiensis*[23] and 70% *vs*. 37% with *An. funestus* s.l. in Tanzania.[24] Neither of these new AIs, pyriproxifen or chlorfenapyr, are related to one another nor show cross resistance. However, to receive a WHO public health recommendation, these "next-generation" LLINs still need to be evaluated in two RCT[25] to demonstrate their effectiveness against malaria in human populations in areas characterized by different insecticide resistance intensities and major vector species.

For peer review only

For peer review only - http://bmjopen.bmj.com/site/about/guidelines.xhtml

In Tanzania, insecticide resistance has spread rapidly[26-28]. In regions where resistance is particularly strong, such as North-West Tanzania, the prevalence of malaria infection remains high (40% in children under 5 years old), despite universal coverage of s-LLINs.[29] This finding echoes reports from Uganda, where no reduction in malaria incidence was observed after the distribution of s-LLINs.[30] As operational failure of standard nets is occurring more frequently in areas with pyrethroid resistance, including the Great Lakes Zone,[30] the newly-developed "next-generation" LLINs now require urgent comparative evaluation.

Here we describe the study design and methodology of an RCT, assessing the effectiveness and cost-effectiveness of three novel vector control interventions (Dual-AI LLINs incorporating mixtures of insecticide classes or insecticide synergists), compared to the standard best practice of pyrethroid-only LLINs, to prevent malaria in an area of pyrethroid resistance in Tanzania. Each putative new class needs to show high effectiveness versus the s-LLIN. None should select for stronger resistance to pyrethroid. Ideally, none should select for resistance to the other new classes of Dual-AI LLIN being tested. A four arm trial is a highly efficient design, should demonstrate the relative effectiveness of each against malaria, and the merits of each product against the same fauna of vector species and human cultural group. The trial should provide insight into future rotational strategies of deployment, their potential to manage insecticide resistance while controlling malaria.

The durability and bio-efficacy of the Dual-AI LLIN are also being evaluated as per WHO guidelines[31] and will be presented in a separate protocol, published elsewhere. The study protocol is reported in line with the Standard Protocol Items: Recommendations for Interventional Trials (SPIRIT) 2013 Statement.[32]

For peer review only

Study Objectives

The primary clinical and entomological objectives are to assess the effectiveness of py-PPF-LLINs, py-CFP-LLINs and py-PBO-LLINs compared to s-LLINs:

- On malaria infection prevalence in children from 6 months to 14 years over two years post intervention.
- On the entomological inoculation rate (EIR) of malaria vectors collected indoors over two years post intervention.

The primary economic objective is to assess the cost-effectiveness of py-PPF-LNs, py-CFP-LNs, py-PBO-LNs and s-LLINs relative to one another.

All secondary objectives are detailed in Figure 1.

Methods and Analysis

Study setting

Five potential study sites in Tanzania's Victoria lake zone were evaluated based on four criteria: report of a minimum of 30% malaria infection prevalence in total population, *An. gambiae* sensu stricto (s.s.) or *An. funestus* s.s. as the main vectors, insecticide pyrethroid resistance in standard WHO bioassays (<50% mortality), and no indoor residual spraying (IRS) planned for the next three years. Data from published and unpublished sources (including the National Malaria Control Program, NMCP; and the President's Malaria Initiative, PMI) were examined and complemented with data collection in November 2017, as

For peer review only

appropriate. Only the district of Misungwi (2°51'00.0"S, 33°04'60.0"E), on the southern border of Lake Victoria in Tanzania met all the criteria.

Misungwi covers an area of 2,122km^2 and includes 27 wards, 78 villages, and a population of 351,607 people.[33] Average altitude in the study area is 1150 meters. The annual rainfall ranges from 0.5 mm to 58.8 mm, split in two rainy seasons (October-December and March-May) and interrupted by a distinct long dry season (June to August/September) and a second short dry season in late December-February.[34]

Misungwi has moderate to high malaria transmission. In a study conducted in 2010, prevalence was 52% across all age groups.[35] During the preliminary assessment in May 2018, presence of all main Tanzanian malaria vectors, *An. gambiae* s.s., *An. arabiensis* and *An. funestus* s.s., were found in the area (unpublished data). WHO insecticide resistance tests were also performed and 24-hour mortality in wild caught *An. gambiae* s.s. exposed to permethrin and deltamethrin was 7% and 19%, respectively. There was also evidence of pyrethroid resistance in *An. funestus* s.s. (mortality 50%) and *An. arabiensis* (mortality 65.3%). Pyrethroid insecticide resistance has also been observed in the adjacent district of Magu and other lake zone regions, such as Geita and Kagera.[28]

The main vector control interventions in Misungwi are universal coverage of LLINs and IRS. The last distribution of LLINs and IRS campaign using Actellic 300CS were carried out in Misungwi district in 2015. In 2017, larviciding was done in some parts of the district.[36] The district is also following national malaria control measures such as intermittent preventive treatment of malaria in pregnant women.

For peer review only

Study design

An overview of the trial design is given in Figure 2. The design is a four parallel-arm, single-blind, superiority cluster randomised trial with village hamlet as the unit of randomisation and repeated cross-sectional survey. The four study arms are:

1. Mixture py-PPF-LLIN: Royal Guard® [intervention 1]
2. Mixture py-CFP-LLIN: Interceptor® G2 [intervention 2]
3. Py-PBO-LLIN: Olyset™ Plus [intervention 3]
4. S-LLIN: Interceptor® [control arm]

The inhabitants of each cluster are blinded to the type of nets they received, as are the field staff who will collect entomological and clinical data. Nets of each type are similar in appearance apart from a colour-coded loop and a unique identifying number. Only the principal investigator and data manager know which code represents each intervention.

Mapping and cluster formation

Every building of 72 villages comprising 453 hamlets from 17 wards was mapped using a Global Positioning System (GPS) handheld unit (Garmin Legend e-trex) and ExpertGPS v3.8 (TopoGrafix) software. A short Open Data Kit (ODK) programmed questionnaire, including name of the head of the household, number of people living in the house, and number of children in each age group (6 months to 59 months, 5 years to 10 years, 11 to 14 years) was recorded for each mapped household.

Wards to map were selected based on both malaria incidence data collected from all health facilities in Misungwi, and *Anopheles* species composition (only areas with the main malaria

For peer review only

vectors *An. funestus* s.s. and *An. gambiae* s.s were considered), assessed during pilot mosquito monitoring from all villages.

As in previous studies,[8] clusters were designed with core and buffer areas to reduce the likelihood of spill-over of intervention effects from one cluster to another. Nets will be distributed to all households in a given cluster (i.e. core and buffer areas), but monitoring of outcomes will be restricted to households situated in the cluster core. A total of 86 clusters were formed from the 72 villages (Figure 3) using the spatial analyst toolbox in ArcGIS (ESRI, Redlands, USA) based on the following criteria: no sub-division of village hamlets, minimum of 150 households with children aged 6 months to 14 years in the core area, and a minimum 600-meter buffer distance. The number of households in each cluster varied from 172 to 2390 (urban area of Misungwi) with an average of 492 households. A 600-meter buffer (i.e. 600 meters between the margins of core areas for any two adjacent clusters) was allocated thereby retaining 73.6% (31,125/42,314) of the households in the core area for sampling and data collection, with 134 to 828 core area households per cluster (Figure 3, panel C). Only two clusters did not meet the criteria of 150 households in the core area, and were therefore excluded.

Randomisation

After the completion of the baseline survey, covariate constrained randomisation was used to allocate the 84 clusters across the four study arms. Covariate constrained allocation ensures that the arms are balanced overall by excluding allocations where pre-determined factors are not balanced within set margins.[37] The following factors were constrained: baseline (pre-intervention) malaria infection prevalence in children aged 0.5 to 14 years, previous LLIN

For peer review only - http://bmjopen.bmj.com/site/about/guidelines.xhtml

usage, socio-economic status (SES), population size, suitable conditions for *An. gambiae* s.s., and for *An. funestus* s.l.. Suitability for each *Anopheles* species was determined using an ecological niche modelling approach (Supplementary file 1) as it was not possible to assess the species composition in all clusters before the randomisation. From among all possible allocations meeting the balancing constraints, one allocation was selected at random. The randomisation was performed by independent statistician.

Interventions

In all four trial arms, net distribution was carried out in an identical manner by the Tanzania Communication and Development Center (TCDC), a Tanzanian not-for-profit organisation, and supervised by the NMCP to follow national net distribution campaign guidelines.[38] All households, enumerated in the core and buffer area, were allocated one net for every two people as recommended by the Tanzania NMCP.[38] Information, education and communication (IEC/SBCC) activities were conducted by a Tanzanian NGO, Tulonge Afia, before, during and after the net distribution to increase usage in the study area and supported by the US President Malaria Initiative. Householders were not asked to return their old net but to use the new net provided. To maximize effective LLIN coverage, two door-to-door hang-up campaigns were done after two weeks and again three months after distribution.

All nets distributed were blue and rectangular (180 cm length x 160 cm wide x 180 cm high). They differed by study arm as follows:

Control: Interceptor® LN (BASF Corporation, Germany) is a pyrethroid-only LLIN with alpha-cypermethrin at a target dose of 200 mg/m2, coated onto polyester filaments. Pyrethroids are neurotoxic insecticides, which target the nervous system of insects.

For peer review only

Interceptor® LN was chosen as a direct comparison to Interceptor® G2 LN and Royal Guard® LN, as they are all impregnated with the same pyrethroid (alpha-cypermethrin); some insecticide resistance mechanisms involving CYPs are specific to type I (permethrin) or type II (alpha-cypermethrin) pyrethroids.

Intervention 1: Royal Guard® LN (Disease Control Technologies, LLC) is a mixture LLIN made of polyethylene incorporating 225 mg/m^2 pyriproxyfen and 216 mg/m^2 alpha-cypermethrin. PPF is known to disrupt female mosquito reproduction and fertility of eggs, stopping the production of the next generation of mosquitoes.[39] PPF may be transferred by auto-dissemination; the transfer of small but toxic amounts of this highly potent insecticide on the tarsi (feet) of female mosquitoes to breeding sites where it can also act as a larvicide.[40]

Intervention 2: Interceptor® G2 LN is a mixture LLIN made of polyester coated with a wash-resistant formulation of 200 mg/m^2 chlorfenapyr and 100 mg/m^2 alpha-cypermethrin. Chlorfenapyr disrupts cellular respiration and oxidative phosphorylation in mitochondria, and due to this unique mode of action is toxic against mosquitoes that are resistant to standard neurotoxic insecticides like pyrethroids.[41]

Intervention 3: Olyset® Plus LN (Sumitomo Chemical) is a mixture LLIN combining PBO (400mg/m^2) and the repellent pyrethroid permethrin (800 mg/m^2) incorporated into polyethylene fibres. PBO is a chemical synergist which acts by inhibiting mixed function oxidases, preventing detoxification of the pyrethroid insecticide. Two RCTs demonstrated that Olyset® Plus LLINs were more effective than Olyset® Net LN (the standard of care in Tanzania), in areas with pyrethroid resistance.[8 12]

Study outcomes

For peer review only - http://bmjopen.bmj.com/site/about/guidelines.xhtml

Primary outcomes

The primary outcomes (Table 1) will be

- Malaria infection (by mRDT) in children aged 6 months to 14 years old at 24 months post intervention.
- Entomological inoculation rate (EIR) as a measure for malaria transmission in the primary vector species.
- Cost-effectiveness of each of the four net types relative to one another.

Secondary outcomes

- Incidence of malaria cases in children aged 6 months to 10 years (measured over 24 months follow up).
- Moderate and severe anaemia in children under 5 years old (<8 g/dL) at 12, 18, 24, 30 and 36 months.
- Malaria infection (by mRDT) in children aged 6 months to 14 years at 12, 18, 30 and 36 months.
- Changes in frequency and intensity of phenotypic and genotypic resistance to pyrethroids (alpha-cypermethrin and permethrin), PBO, CFP and PPF.
- Other entomological outcomes: changes in mosquito resting behaviour indoors and outdoors, species composition and density ratio, host feeding, mosquito ovary development and fecundity.
- Incremental financial cost to the provider of each of the four net types relative to one another.

For peer review only

Data collection

Intervention coverage data

LLIN coverage will be evaluated 6 months after distribution and during each cross-sectional survey. Three indicators will be used:[42] 1)"proportion of households with at least one LLIN for every two people(ownership)", 2)"proportion of household with enough LLINs to sleep under (access)" and "proportion of households declaring using a LLIN (study or not) last night (usage)".

Clinical data

To determine infection prevalence, repeated cross-sectional surveys will be conducted at 12, 18, 24, 30 and 36 months after net distribution (Table 1). At each time point, 45 households will be randomly selected from the core sampling area of each cluster, using the census list generated during baseline enumeration. In each house a maximum of two children, between 6 months and 14 years old, will be selected. Accounting for households that are closed, refusing informed consent, or do not have children of the required age at the time of the survey, this sampling strategy is expected to yield an average of 30 enrolled houses with 50 children per cluster based on previous studies. [8 43] A total of 4200 children are thus expected to be surveyed at each time point.

Inclusion criteria are households with at least one child between 6 months to 14 years old who permanently resides in the selected household and an adult caregiver who can provide written consent. Exclusion criteria include dwellings not found or vacant during the survey, no adult caregiver capable of giving informed consent, or eligible children are severely ill.

For peer review only

Information on sex, age distribution, educational status and occupation, SES, house structure, vector control measures used, past malaria cases, net coverage and care seeking behaviour will be collected. Fever or history of fever in the past 48 hours will be recorded for every child selected. Temperatures will be taken, and each child tested for malaria using mRDTs, (CareStart RDTs; HRP2, (pf), DiaSys, Wokingham, UK) and haemoglobin levels measured (HemoCue Hb 201+ (Aktiebolaget Leo Diagnostics, USA)). When the mRDT is positive, free treatment for malaria will be provided with artemether-lumefantrine (artemisinin-based combination therapy; ACT), as per the national guidelines. Children with severe malaria or any other diseases that cannot be treated by the team will be referred to the nearest health facility.

To assess malaria case incidence, 35 children per cluster, aged 6 months to 10 years old, will be randomly selected and followed up every two weeks during the high transmission season (October-July) and every month during the low transmission season (August-September), over a 12 months period (Table 1). To reduce attrition, another cohort of children will be selected at the beginning of the second year and will also be followed for 12 months. All cohort children will be cleared of malaria infection at the beginning of the first and second year by ACT treatment. Cohort children will then be checked two weeks later by mRDT and microscopy (if the mRDT is positive) to confirm whether they have been cleared. During each bi-weekly or monthly visit, children with fever ≥37.5°C and/or history of fever for the past 48 hours will be tested for the presence of malaria parasites (case incidence) by mRDT. For those with positive mRDT results, a blood slide will also be taken to confirm malaria positivity and they will also be treated, as previously described. Children will be encouraged to visit a health facility in case they have fever, or get sick at any time between visits. Each

For peer review only - http://bmjopen.bmj.com/site/about/guidelines.xhtml

child will be provided with a personal medical book where malaria episodes will be recorded during study visits or whenever they attend a health facility and also will be provided with medical insurance. During cohort visits, a questionnaire will also be administered to inquire about net usage the night before the visit, any adverse events encountered and travel history within the past two weeks.

Entomological data

Cross-sectional entomological surveys will be carried out in 84 study clusters to monitor the indoor mosquito population density (Table 1). Each cluster will be visited once every quarter; each month, 7 clusters from each study arm will be selected. Indoor mosquito densities will be monitored using Centers for Disease Control and Prevention (CDC) Miniature light traps (John W Hock Company, USA) in 8 randomly selected households in the core area of each cluster. CDC light trap will be installed at the feet of one bed and existing net substituted with a project standard LLIN, and replaced the following day. For each of the selected houses, a short questionnaire will be administered to collect information about the number of inhabitants, type of house construction materials, presence of animals, coverage and usage of nets, and other malaria prevention measures used by household members. Sampled mosquitoes will be identified morphologically following the identification key by Gillies and Coetzee.[44] Parity rates will be estimated in a subset of live *Anopheles* mosquitoes through dissection.[45] About 10 *An. gambiae* s.l and 10 *An. funestus* s.l will be randomly picked per household per collection night and preserved for laboratory analysis. This sample of mosquito specimens will be screened for *Plasmodium falciparum* circumsporozoite protein (Pf-CSP) by ELISA.[46] The CSP-ELISA positive samples will be re-analysed by heating the ELISA lysates to remove any false positives.[47]

For peer review only

PCR TaqMan assays will be used to discriminate members of *An. gambiae* species complex,[48] and members of the *An. funestus* group[49] on a sub sample of *Anopheles* collected. A sample of *An. gambiae* s.s. and *An. arabiensis* will be genotyped for the L1014F-*kdr* and L1014S-*kdr* mutations, associated with pyrethroid and DDT resistance, using TaqMan PCR assays, following the protocol by Bass *et al*.[50]

Phenotypic resistance and resistance levels to alpha-cypermethrin, permethrin, pyriproxyfen and chlorfenapyr, will be characterized at baseline and yearly post-intervention, in all four study arms, using WHO cylinder and modified CDC bottle bioassays.[51 52] The PBO synergist effect on wild female *An. gambiae* s.l. and *An. funestus* s.l. will be assessed using pre-exposure to PBO followed by permethrin resistance intensity assays. All knockdown/dead mosquitoes at 60 minutes and surviving 72 hours post-exposure to insecticides will be stored individually in RNAlater® and preserved at -20°C for gene expression analysis. RNA will be extracted from pools of *An. gambiae* s.s. or *An. funestus* s.s and cDNA synthesized, according to standard procedures. Relative expression of CYPs and other metabolic enzymes, previously identified as being over-expressed in resistant *An. gambiae* s.s. and *An. funestus* s.s. populations in Tanzania,[53] will be measured using multiplex TaqMan RT-qPCR assays.[54]

Malaria vector abundance, species composition, feeding and resting behaviours (including biting time, host preference), and contribution to outdoor malaria transmission will be assessed at baseline and after implementation of the interventions to assess changes over time in 10 clusters per treatment arm. CDC light trap and adapted Furvela tent traps[55] will be used for indoor and outdoor collection of free flying mosquitoes. Resting *Anopheles* will be collected using CDC prokopack aspirators. Sub samples of *Anopheles* will be subjected to the

For peer review only - http://bmjopen.bmj.com/site/about/guidelines.xhtml

same laboratory tests than routine collection. Additional information about mosquito collection methods is available in the Supplementary file 1.

Economic data

Data on resource use and unit costs will be collected from primary and secondary sources, including discussions with net manufacturers, donors, and experts; review of project, donor, and Ministry of Health records; trial survey data on care seeking behaviour; published literature; and WHO-CHOICE unit cost estimates.[56] Data on health outcomes will include primary trial data on malaria incidence and anaemia, as well as secondary data on the age structure of malaria incidence and life expectancy.[57 58]

For peer review only

Table 1: Trial outcome measurements

Outcome	Measurement	Collection	Frequency
**Clinical outcomes**			
Malaria infection prevalence	Rapid diagnostic test	Cross sectional survey	Baseline, 12, 18, 24, 30, and 36 months post intervention
Anaemia	Haematocrit	Cross sectional survey	Baseline, 12, 18, 24, 30, and 36 months post intervention
Temperature	1.Digital ear thermometer all children<br>2.Temperature and history of fever	1.Cross sectional survey<br>2.Cohort follow up	1.Baseline, 12, 18, 24, 30 and 36 months post intervention<br>2.Every month
Malaria case	Rapid diagnostic test taken when fever ≥37.5°C and or history of fever for the past 48 hours	Cohort follow up	Every two weeks during high transmission season (October-July) and every month during dry season (August-September)
**Measurement of entomological outcomes**			
Indoor *Anopheles* density	CDC light traps	Entomology surveillance	8 houses per cluster every 3 months in all clusters for 3 years
Outdoor biting	Adapted Furvela tent trap[55]	Sentinel site	Twice a year in 2-3 sentinel site per arm.
Mosquito sporozoite rate	CSP-ELISA to estimate EIR[46]	Entomology surveillance	Sub sample (30%) of the mosquito collected in CDC light trap
Anopheles species identification	*1.An. funestus* s.l. complex: conventional PCR or multiplex real time PCR[49]<br>*2.An. gambiae* s.l. complex: Taq Man real time PCR[48]	Entomology surveillance & Sentinel site & resistance test	Sub sample of mosquitoes collected
Insecticide resistance frequency and intensity	WHO cylinder assay[52]<br>CDC bottle bioassays[51]	Collection of adult *Anopheles* resting indoors	Once a year in a sub sample of clusters.
Frequency of Vgsc mutation	TaqMan PCR[50]	Entomology surveillance	Sub sample of mosquitoes collected in light trap
Insecticide resistance mechanisms	Multiplex TaqMan Reverse-transcription quantitative PCR (RT-qPCR) will be used to monitor expression of CYPs and other metabolic enzymes known to be over-expressed in resistant *An. gambiae* s.s. and *An. funestus* s.s. populations from previous studies in Tanzania[54]	Collection of adult *Anopheles* resting indoors, previously phenotyped in resistance bioassays	At baseline and at each post intervention year in a sub sample of clusters

For peer review only - http://bmjopen.bmj.com/site/about/guidelines.xhtml

Sample size and power consideration

Malaria prevalence cross-sectional survey

The sample size was calculated using the method of Hayes and Bennett, taking into account the cluster-randomised design.[59] For the primary outcome prevalence of infection at 24 months, we assumed a malaria prevalence in the reference arm of 40%, an average of 50 individuals per cluster, and a coefficient of variation of 21%, based on recent surveys in a similar area.[60] To achieve 80% power to detect a prevalence ratio of 0.72, i.e, a 28% lower prevalence in at least one of the intervention arms versus the reference at Bonferroni-corrected significance level of 1.67%, we require 21 clusters per arm. This calculation is conservative as it does not account for the repeated malaria prevalence measures; thus, we anticipate being able to detect even smaller differences.

Incidence of malaria infection in children cohort

Sample size calculations for the secondary outcome (cumulative malaria incidence) were based on the method of Hayes and Bennett.[59] Based on a previous study (Jacklin Mosha, personal communication), it was assumed that the mean number of malaria episodes per child per year in the reference arm was 0.85 (monthly event rate of 0.071) with a between-cluster coefficient of variation of 21%. With a cohort of 35 children per cluster (21 clusters per arm), and accounting for attrition of 30% over 24 months, we would achieve 80% power to detect a 23.6% relative reduction in malaria cases per child per year (risk ratio 0.764) between at least one intervention arm relative to the reference arm, using a two-sided Bonferroni-corrected significance level of 1.67%. If the incidence in the reference arm is lower, at 0.5

For peer review only

episodes/year, we will still have 80% power to detect a 26.1% relative reduction (relative risk 0.739).

Entomological survey for EIR estimation

Sample size calculations for the entomological survey were based on the method of Hayes and Bennett.[59] It was assumed that the mean EIR (number of infectious bites per household per month) in the reference arm was 1.76 with a between-cluster coefficient of variation of 40%, based on a previous study.[8] With a sample of 8 households per cluster and sampling each cluster every quarter we will have collections for 32 house-nights per cluster per year. With 21 clusters per arm the study would achieve 80% power to detect a 36% relative reduction in monthly EIR (relative risk 0.64) between at least one of the intervention arms relative to the reference using a Bonferroni-corrected significance level of 1.67%. Changes in insecticide resistance frequency and resistance management potential will be assessed separately from light trap and household resting collections.

Data Management

Clinical and entomological measurements in the cohort study and the cross-sectional surveys will be captured on electronic forms using tablets/smartphones installed with open data kit (ODK) and uploaded daily onto the server at London School Hygiene and Tropical Medicine (LSHTM). Other data and clinical measurements during the cross-sectional survey, recorded on paper CRFs, will be double-entered into an access database independently by two data clerks. Laboratory data outputs will be available directly from the analyser (e.g. ELISA data) and imported into a database. All databases will maintain an audit trail with time-date stamps

For peer review only - http://bmjopen.bmj.com/site/about/guidelines.xhtml

of data entry and all changes that are made to the data. Anonymized study numbers will be used as unique participant identifiers.

Data analysis

Primary outcomes

Prevalence of malaria infection and entomological inoculation rate

The primary analysis will be conducted using the intention to treat (ITT) approach. Secondary per-protocol analyses will also be conducted. The primary outcome, measured at 12, 18, 24, 30 and 36 months after net distribution, will be analysed using mixed effects logistic regression. The unit of analysis will be the individual. The model will include fixed effects for time, study arm, and time by study arm, and will adjust for the baseline prevalence in each community as a covariate, as well as the covariates used in the covariate-constrained allocation procedure. The model will account for within-period and between-period intra-cluster correlations.[61]

Prevalence of malaria infection in each Dual-AI LLIN treatment arm will be compared to the prevalence of infection in the reference arm using least square mean differences to assess whether the new LLINs are superior. The primary comparison will occur at the end of the 24 months post intervention period. To adjust for the increased risk of a Type I error due to multiple pairwise comparisons, the level of significance will be adjusted using the Bonferroni method. Least square mean differences between each intervention arm versus control will be calculated at each time, together with 98.23% confidence intervals (reflecting adjustment of usual 95% CI to account for multiple comparisons). Secondary analyses will include all

For peer review only - http://bmjopen.bmj.com/site/about/guidelines.xhtml

possible pairwise comparisons of Dual-AI LLIN arms.[62] Sub-group analyses will be performed to investigate the impacts of interventions according to different individual (i.e. age, sex), household (i.e. wealth, distance to health facilities) and cluster-level (i.e. vector resistance intensity, vector species composition) characteristics.

EIR will be estimated as the mean number of sporozoite infected mosquitoes per house per night (by species and overall) and weighted to account for proportion of mosquitoes processed for sporozoites. Differences in *Anopheles* density and EIR between the different arms will be estimated using random effects negative binomial regression taking into account the intracluster correlation. Random effects logistic regression will be used to compare sporozoite rate between study arms.

Economic evaluation

Following relevant guidelines,[63 64] we will combine primary trial data with secondary data in a decision analytic model using a decision tree. We will adopt a societal perspective, presenting costs both combined and disaggregated by payer, and model effects and costs over a lifetime horizon. In the main analysis, we will assume that nets are distributed in mass campaigns every three years, reflecting both what was done in the trial and standard practice in malaria control.

Effects will be presented as DALYs (discounted at 3% and with no age weighting), calculated as the sum of years of life with disability from malaria-related illness (uncomplicated and severe malaria cases and anaemia) and years of life lost from malaria deaths.[63] The number of cases associated with each intervention will be modelled as the product of the incidence in the reference arm, the incidence rate ratio of the relevant arm to

For peer review only

the reference arm, and a standardized population size. Incidence in children aged 6 months to 10 years will reflect ITT trial data, while incidence in other age groups, which comprise a small share of overall cases and deaths, will be estimated as a function of the incidence in children based on publicly available modelling.[58 65] DALYs will be calculated as the product of the estimated number of malaria cases (as above), the case fatality rate, and the remaining life expectancy at age of death. Rates of progression to severe disease and to death will be estimated to reflect real-world outcomes based on secondary data. In the main analysis (based on trial data at 24 months), possible bounds for incidence rate ratios over months 25-36 will be estimated based on annual rate ratios over 0-12 and 13-24 months and assuming no relative effect compared to s-LLINs. In additional analyses, effects will also be presented as malaria cases and percentage point reduction in malaria prevalence.

We will plot the costs and effects of each intervention on the cost-effectiveness plane, identify the cost-effectiveness frontier and expansion path, and calculate incremental cost-effectiveness ratios (ICERs) between adjacent points on this path. Deterministic and probabilistic sensitivity analysis will explore the impact of uncertainty and heterogeneity on cost-effectiveness results. Cost-effectiveness acceptability curves[66] will indicate the probability of each strategy being the most cost-effective choice at plausible cost-effectiveness thresholds.[67 68] Affordability will be explored by comparing additional financial costs (net of cost savings) per person to relevant expenditure levels. Analyses will be conducted in Microsoft Excel with Visual Basic for Applications.

Secondary outcomes

Incidence

For peer review only - http://bmjopen.bmj.com/site/about/guidelines.xhtml

Cumulative incidence of malaria infection over the 24 months follow up (accounting for repeat episodes in the same child but measured on separate cohorts at 12 and 24 months post-intervention) will be analysed using mixed effects Poisson or negative binomial regression. The model will include fixed effects for time, study arm, and time by study arm, and will adjust for the covariates used in the covariate-constrained allocation procedure. The model will account for within-period and between-period intra-cluster correlations. Least square mean differences will be obtained from the model to compare each intervention versus the control. Follow-up within 14 days of a previous episode in the same child will be censored and will not be included in the analysis. An offset for duration of follow-up will be included in the model to account for attrition.

Prevalence of anaemia

Prevalence of moderate and severe anaemia in children under 5 at 12,18, 24, 30 and 36 months will be analysed using mixed effects logistic regression similar to the approach outlined for malaria prevalence.

Insecticide resistance monitoring

Bioassay data will be interpreted according to the updated WHO guidelines: mortality of ≥98% indicates susceptibility at the diagnostic dose, mortality of 90-97% is suggestive of resistance, and mortality of less than 90% indicates resistance.[69] For resistance intensity assays at 5X insecticide concentrations, mortality ≥98% indicates low intensity resistance and mortality <98% indicates moderate to high intensity resistance. For resistance intensity assays at 10X insecticide concentrations, mortality ≥98% indicates moderate intensity resistance and mortality <98% indicates high intensity resistance.

For peer review only - http://bmjopen.bmj.com/site/about/guidelines.xhtml

For metabolic gene assays, relative expression level and Fold Change (FC) of each target gene from resistant and susceptible field samples, relative to the susceptible laboratory strain (*An. gambiae* s.s Kisumu or *An. funestus* s.s FANG), will be calculated using the $2^{-\Delta\Delta CT}$ method, incorporating PCR efficiency and normalised relative to the endogenous housekeeping control gene.[70]

Patient and public involvement

There was no patient or public involvement in the design of this study. Communities will be involved in the implementation of the interventions and study activities through their leaders and community health representatives.

Ethics approval

This protocol has been reviewed and approved by all institutional review boards, including: the Medical Research Coordinating Committee (MRCC) of the National Institute for Medical Research (NIMR), London School of Hygiene and Tropical Medicine (LSHTM), Kilimanjaro Christian Medical University College (KCMUCO); and University of Ottawa.

This study will be conducted according to the Declaration of Helsinki and the International Guidelines for Ethical Review of Epidemiological Studies. All field and clinical staff as well as the principal investigator will receive training on good clinical and laboratory practice before data collection begins, and refresher training every year. For all data collection activities (epidemiological and entomological), written informed consent (Supplementary file 2) will be obtained from an adult guardian in the household. The consent form will be written in Swahili and indicate the purpose of the study, the procedures, risks and benefits, that

For peer review only

For peer review only - http://bmjopen.bmj.com/site/about/guidelines.xhtml

participation is completely voluntary, and that they may withdraw at any time with impunity. The study questionnaire will also be administered in Swahili.

Dissemination

Study findings will be shared in stakeholder meetings attended by local community leaders, the Ministry of Health, Community Development, Gender, Elderly and Children, the National Malaria Control Programme, the President's office regional administration and local government representatives. Results will also be shared through peer-reviewed publications, at scientific conferences, and through clinicaltrials.gov.

Figure Titles

Figure 1: Secondary objectives

Figure 2: Trial study design

Figure 3: Study area. Map showing Misungwi study area in Mwanza region, North Tanzania (panel A), the 85 study clusters identified with core and buffer area and intervention allocation (panel B). Panel C shows a closer map on the minimum 600 hundred meters area between houses in adjacent clusters.

For peer review only

For peer review only - http://bmjopen.bmj.com/site/about/guidelines.xhtml

References

1. WHO. World Malaria Report Geneva, Switzerland, 2018.

2. WHO. World malaria report. Geneva, Switzerland, 2019.

3. Asidi A, N'Guessan R, Akogbeto M, et al. Loss of household protection from use of insecticide-treated nets against pyrethroid-resistant mosquitoes, benin. *Emerg Infect Dis* 2012;18(7):1101-6. doi: 10.3201/eid1807.120218

4. Ochomo EO, Bayoh NM, Walker ED, et al. The efficacy of long-lasting nets with declining physical integrity may be compromised in areas with high levels of pyrethroid resistance. *Malar J* 2013;12:368. doi: 10.1186/1475-2875-12-368

5. Kleinschmidt I, Bradley J, Knox TB, et al. Implications of insecticide resistance for malaria vector control with long-lasting insecticidal nets: a WHO-coordinated, prospective, international, observational cohort study. *The Lancet infectious diseases* 2018;18(6):640-49. doi: 10.1016/S1473-3099(18)30172-5

6. Lindblade KA, Mwandama D, Mzilahowa T, et al. A cohort study of the effectiveness of insecticide-treated bed nets to prevent malaria in an area of moderate pyrethroid resistance, Malawi. *Malar J* 2015;14:31. doi: 10.1186/s12936-015-0554-1

7. Ochomo E, Chahilu M, Cook J, et al. Insecticide-Treated Nets and Protection against Insecticide-Resistant Malaria Vectors in Western Kenya. *Emerg Infect Dis* 2017;23(5):758-64. doi: 10.3201/eid2305.161315

8. Protopopoff N, Mosha JF, Lukole E, et al. Effectiveness of a long-lasting piperonyl butoxide-treated insecticidal net and indoor residual spray interventions, separately and together, against malaria transmitted by pyrethroid-resistant mosquitoes: a cluster, randomised controlled, two-by-two factorial design trial. *Lancet* 2018;391(10130):1577-88. doi: 10.1016/S0140-6736(18)30427-6

9. WHO. Report of the 12th WHOPES Working Group meeting. Geneva, Switzerland: World Health Organization, 2008:41-75.

10. WHO. Conditions for use of long-lasting insecticidal nets treated with a pyrethroid and piperonyl butoxide: World Health Organization, 2015.

11. WHO. Conditions for deployment of mosquito nets treated with a pyrethroid and piperonyl butoxide. Geneva: World Health Organization, 2017.

For peer review only

12. Staedke SG, Gonahasa S, Dorsey G, et al. Effect of long-lasting insecticidal nets with and without piperonyl butoxide on malaria indicators in Uganda (LLINEUP): a pragmatic, cluster-randomised trial embedded in a national LLIN distribution campaign. *Lancet* 2020;395(10232):1292-303. doi: 10.1016/S0140-6736(20)30214-2

13. N'Guessan R, Odjo A, Ngufor C, et al. A Chlorfenapyr Mixture Net Interceptor(R) G2 Shows High Efficacy and Wash Durability against Resistant Mosquitoes in West Africa. *PLoS One* 2016;11(11):e0165925. doi: 10.1371/journal.pone.0165925

14. Ngufor C, N'Guessan R, Fagbohoun J, et al. Efficacy of the Olyset Duo net against insecticide-resistant mosquito vectors of malaria. *Sci Transl Med* 2016;8(356):356ra121. doi: 10.1126/scitranslmed.aad3270

15. Djenontin A, Ahoua Alou LP, Koffi A, et al. Insecticidal and sterilizing effect of Olyset Duo(R), a permethrin and pyriproxyfen mixture net against pyrethroid-susceptible and -resistant strains of Anopheles gambiae s.s.: a release-recapture assay in experimental huts. *Parasite* 2015;22:27. doi: 10.1051/parasite/2015027

16. Koffi AA, Ahoua Alou LP, Djenontin A, et al. Efficacy of Olyset(R) Duo, a permethrin and pyriproxyfen mixture net against wild pyrethroid-resistant Anopheles gambiae s.s. from Cote d'Ivoire: an experimental hut trial. *Parasite* 2015;22:28. doi: 10.1051/parasite/2015028

17. Kawada H, Dida GO, Ohashi K, et al. A small-scale field trial of pyriproxyfen-impregnated bed nets against pyrethroid-resistant Anopheles gambiae s.s. in western Kenya. *PloS one* 2014;9(10):e111195. doi: 10.1371/journal.pone.0111195

18. Bayili K, N'do S, Namountougou M, et al. Evaluation of efficacy of Interceptor((R)) G2, a long-lasting insecticide net coated with a mixture of chlorfenapyr and alpha-cypermethrin, against pyrethroid resistant Anopheles gambiae s.l. in Burkina Faso. *Malaria journal* 2017;16(1):190. doi: 10.1186/s12936-017-1846-4

19. Camara S, Ahoua Alou LP, Koffi AA, et al. Efficacy of Interceptor((R)) G2, a new long-lasting insecticidal net against wild pyrethroid-resistant Anopheles gambiae s.s. from Cote d'Ivoire: a semi-field trial. *Parasite* 2018;25:42. doi: 10.1051/parasite/2018042

20. Ngufor C, Fagbohoun J, Critchley J, et al. Which intervention is better for malaria vector control: insecticide mixture long-lasting insecticidal nets or standard pyrethroid nets combined with indoor residual spraying? *Malaria journal* 2017;16(1):340. doi: 10.1186/s12936-017-1987-5

21. Ngufor C, Agbevo A, Fagbohoun J, et al. Efficacy of Royal Guard, a new alpha-cypermethrin and pyriproxyfen treated mosquito net, against pyrethroid-resistant malaria vectors. *Sci Rep* 2020;10(1):12227. doi: 10.1038/s41598-020-69109-5 [published Online First: 2020/07/24]

22. Tiono AB, Ouedraogo A, Ouattara D, et al. Efficacy of Olyset Duo, a bednet containing pyriproxyfen and permethrin, versus a permethrin-only net against clinical malaria in an area with highly pyrethroid-resistant vectors in rural Burkina Faso: a cluster-randomised controlled trial. *Lancet* 2018;392(10147):569-80. doi: 10.1016/S0140-6736(18)31711-2

23. WHO. Report of the 20th WHOPES Working Group meeting. Geneva WHO, 2017:4-46.

For peer review only - http://bmjopen.bmj.com/site/about/guidelines.xhtml

24. Tungu P, Kirby M, Malima R, et al. Interceptor(R) long-lasting insecticidal net: phase III evaluation over three years of household use and calibration with Phase II experimental hut outcomes. *Parasit Vectors* 2016;9:204. doi: 10.1186/s13071-016-1490-9

25. WHO. Design of epidemiological trials for vector control products: Report of a WHO expert advisory group. Geneva, Switzerland: WHO, 2017.

26. Kabula B, Tungu P, Matowo J, et al. Susceptibility status of malaria vectors to insecticides commonly used for malaria control in Tanzania. *Trop Med Int Health* 2012;17(6):742-50. doi: 10.1111/j.1365-3156.2012.02986.x

27. Kisinza WN, Nkya TE, Kabula B, et al. Multiple insecticide resistance in Anopheles gambiae from Tanzania: a major concern for malaria vector control. *Malar J* 2017;16(1):439. doi: 10.1186/s12936-017-2087-2

28. Matiya DJ, Philbert AB, Kidima W, et al. Dynamics and monitoring of insecticide resistance in malaria vectors across mainland Tanzania from 1997 to 2017: a systematic review. *Malar J* 2019;18(1):102. doi: 10.1186/s12936-019-2738-6 [published Online First: 2019/03/28]

29. NBS. National Bureau of Statistics, Demographic and Health Survey and Malaria Indicator Survey 2015-2016. Dar Es Salaam, Tanzania: National Bureau of Statistics, 2016.

30. Katureebe A, Zinszer K, Arinaitwe E, et al. Measures of Malaria Burden after Long-Lasting Insecticidal Net Distribution and Indoor Residual Spraying at Three Sites in Uganda: A Prospective Observational Study. *PLoS Med* 2016;13(11):e1002167. doi: 10.1371/journal.pmed.1002167

31. WHO. Guidelines for laboratory and field-testing of long-lasting insecticidal nets: World Health Organization, 2013.

32. Chan AW, Tetzlaff JM, Altman DG, et al. SPIRIT 2013 statement: defining standard protocol items for clinical trials. *Ann Intern Med* 2013;158(3):200-7. doi: 10.7326/0003-4819-158-3-201302050-00583 [published Online First: 2013/01/09]

33. National Bureau of Statistics N. The 2012 Population and Housing Census (PHC) for the United Republic of Tanzania Dar es salaam, Tanzania, 2012.

34. columbia.edu. Early Warning System Rainfall Estimate (RFE) 2020 [Available from: https://iridl.ldeo.columbia.edu/SOURCES/.NOAA/.NCEP/.CPC/.FEWS/.Africa/.DAILY/.RFEv2/.est_prcp.

35. Nkya TE, Mosha FW, Magesa SM, et al. Increased tolerance of Anopheles gambiae ss to chemical insecticides after exposure to agrochemical mixture. *Tanzania Journal of Health Research* 2014;16(4)

36. PMI. Tanzania malaria operational plan FY 2018. https://wwwpmigov/docs/default-source/default-document-library/malaria-operational-plans/fy-2018/fy-2018-tanzania-malaria-operational-plan, 2018.

37. Hayes RJ, Moulton LH. Chapter 6: Randomisation procedures. Cluster Randomised Trials. London: Chapman & Hall/CRC 2009:149-62.

For peer review only

38. Tanzania MoHaSW. National Malaria strategic plan 2014-2020, 2014.

39. Harris C, Lwetoijera DW, Dongus S, et al. Sterilising effects of pyriproxyfen on Anopheles arabiensis and its potential use in malaria control. *Parasit Vectors* 2013;6:144. doi: 10.1186/1756-3305-6-144

40. Lwetoijera D, Harris C, Kiware S, et al. Effective autodissemination of pyriproxyfen to breeding sites by the exophilic malaria vector Anopheles arabiensis in semi-field settings in Tanzania. *Malar J* 2014;13:161. doi: 10.1186/1475-2875-13-161

41. Raghavendra K, Barik TK, Sharma P, et al. Chlorfenapyr: a new insecticide with novel mode of action can control pyrethroid resistant malaria vectors. *Malaria journal* 2011;10:16. doi: 10.1186/1475-2875-10-16

42. Measure D, PMI, RBM, UNICEF, WHO. Household survey indicators for malaria control. Geneva: World Health Organization, 2018.

43. West PA, Protopopoff N, Wright A, et al. Enhanced protection against malaria by indoor residual spraying in addition to insecticide treated nets: is it dependent on transmission intensity or net usage? *PLoS One* 2015;10(3):e0115661. doi: 10.1371/journal.pone.0115661

44. Gillies MTC, M. A supplement to the Anophelinae of Africa south of the Sahara (Afrotropical region)1987.

45. Detinova T, Gillies M. Observations on the determination of the age composition and epidemiological importance of populations of Anopheles gambiae Giles and Anopheles funestus Giles in Tanganyika. *Bulletin of the World Health Organization* 1964;30(1):23.

46. Wirtz RA, Zavala F, Charoenvit Y, et al. Comparative testing of monoclonal antibodies against Plasmodium falciparum sporozoites for ELISA development. *Bull World Health Organ* 1987;65(1):39-45.

47. Durnez L, Van Bortel W, Denis L, et al. False positive circumsporozoite protein ELISA: a challenge for the estimation of the entomological inoculation rate of malaria and for vector incrimination. *Malaria Journal* 2011;10(1):195.

48. Bass C, Williamson MS, Field LM. Development of a multiplex real-time PCR assay for identification of members of the Anopheles gambiae species complex. *Acta Trop* 2008;107(1):50-3. doi: 10.1016/j.actatropica.2008.04.009 [published Online First: 2008/05/21]

49. Vezenegho SB, Bass C, Puinean M, et al. Development of multiplex real-time PCR assays for identification of members of the Anopheles funestus species group. *Malar J* 2009;8:282. doi: 10.1186/1475-2875-8-282

50. Bass C, Nikou D, Donnelly MJ, et al. Detection of knockdown resistance (kdr) mutations in Anopheles gambiae: a comparison of two new high-throughput assays with existing methods. *Malar J* 2007;6:111. doi: 10.1186/1475-2875-6-111

51. Brogdon WG, Chan A. Guideline for evaluating insecticide resistance in vectors using the CDC bottle bioassay. Altlanta, USA: Centers for Disease Control and Prevention, 2012.

For peer review only - http://bmjopen.bmj.com/site/about/guidelines.xhtml

52. WHO. Test procedures for insecticide resistance monitoring in malaria vector mosquitoes (Second edition). Geneva, Switzerland: World Health organization, 2016.

53. Matowo J, Kitau J, Kaaya R, et al. Trends in the selection of insecticide resistance in Anopheles gambiae s.l. mosquitoes in northwest Tanzania during a community randomized trial of longlasting insecticidal nets and indoor residual spraying. *Med Vet Entomol* 2015;29(1):51-9. doi: 10.1111/mve.12090

54. Mavridis K, Wipf N, Medves S, et al. Rapid multiplex gene expression assays for monitoring metabolic resistance in the major malaria vector Anopheles gambiae. *Parasites & vectors* 2019;12(1):9. doi: 10.1186/s13071-018-3253-2

55. Charlwood JD, Rowland M, Protopopoff N, et al. The Furvela tent-trap Mk 1.1 for the collection of outdoor biting mosquitoes. *PeerJ* 2017;5:e3848. doi: 10.7717/peerj.3848

56. Georgios Gkountouras JAL, Anderson Stanciole, Karin Stenberg, and Tessa Tan-Torres Edejer. Estimation of unit costs for general health services: Updated WHO-CHOICE estimates: WHO department of Health Systems Financing, 2011.

57. Organization WH. Life Tables. 2020

58. DALYs GBD, Collaborators H. Global, regional, and national disability-adjusted life-years (DALYs) for 359 diseases and injuries and healthy life expectancy (HALE) for 195 countries and territories, 1990-2017: a systematic analysis for the Global Burden of Disease Study 2017. *Lancet* 2018;392(10159):1859-922. doi: 10.1016/S0140-6736(18)32335-3

59. Hayes RJ, Bennett S. Simple sample size calculation for cluster-randomized trials. *Int J Epidemiol* 1999;28(2):319-26.

60. West PA, Protopopoff N, Wright A, et al. Indoor residual spraying in combination with insecticide-treated nets compared to insecticide-treated nets alone for protection against malaria: a cluster randomised trial in Tanzania. *PLoS Med* 2014;11(4):e1001630. doi: 10.1371/journal.pmed.1001630

61. Kasza J, Hemming K, Hooper R, et al. Impact of non-uniform correlation structure on sample size and power in multiple-period cluster randomised trials. *Stat Methods Med Res* 2019;28(3):703-16. doi: 10.1177/0962280217734981 [published Online First: 2017/10/14]

62. Proschan MA, Waclawiw MA. Practical guidelines for multiplicity adjustment in clinical trials. *Control Clin Trials* 2000;21(6):527-39.

63. Wilkinson T, Sculpher MJ, Claxton K, et al. The International Decision Support Initiative Reference Case for Economic Evaluation: An Aid to Thought. *Value in health : the journal of the International Society for Pharmacoeconomics and Outcomes Research* 2016;19(8):921-28. doi: 10.1016/j.jval.2016.04.015

64. Husereau D, Drummond M, Petrou S, et al. Consolidated Health Economic Evaluation Reporting Standards (CHEERS) statement. *Bmj* 2013;346:f1049. doi: 10.1136/bmj.f1049

65. Evaluation IfHMa. Global Burden of Disease Results Tool. Seattle, USA University of Washington, 2017.

For peer review only

66. Fenwick E, Claxton K, Sculpher M. Representing uncertainty: the role of cost-effectiveness acceptability curves. *Health Econ* 2001;10(8):779-87. [published Online First: 2001/12/18]

67. Ochalek J, Lomas J, Claxton K. Estimating health opportunity costs in low-income and middle-income countries: a novel approach and evidence from cross-country data. *BMJ Glob Health* 2018;3(6):e000964. doi: 10.1136/bmjgh-2018-000964 [published Online First: 2018/11/30]

68. Marseille E, Larson B, Kazi DS, et al. Thresholds for the cost-effectiveness of interventions: alternative approaches. *Bull World Health Organ* 2015;93(2):118-24. doi: 10.2471/BLT.14.138206

69. WHO. Test procedures for insecticide resistance monitoring in malaria vector mosquitoes. Geneva, Switzerland: World Health organisation, 2016.

70. Rao X, Huang X, Zhou Z, et al. An improvement of the 2^(-delta delta CT) method for quantitative real-time polymerase chain reaction data analysis. *Biostat Bioinforma Biomath* 2013;3(3):71-85. [published Online First: 2013/08/01]

For peer review only - http://bmjopen.bmj.com/site/about/guidelines.xhtml

Authors' contribution

JFM helped in the design of the study protocol, wrote the first draft of the manuscript and is coordinating site investigators. MAK, supported the research grant proposal, helped in the design of the study protocol, and substantially revised the manuscript. LAM contributed to the study protocol and the writing of the manuscript. FWM and AM supported the research grant proposal and design of the study, facilitated the obtention of ethical approval and revised the manuscript. CP designed the cost-effectiveness component of the protocol and revised the manuscript. MT gave statistical inputs to the protocol and manuscript. CT supported the mapping and cluster formation, revised, and prepared maps for the manuscript. NM and EL contributed to the study design and revised the manuscript. NP and MR conceptualised the study. MR and IK contributed to the protocol, revised the manuscript and provided technical support. NP designed and drafted the study protocol, contributed to the writing of the manuscript and is the principal investigator of the study. All authors read and approved the final version of the manuscript.

Funding statement

This study is funded under the global health trial a jointly funded initiative from the Department for International Development (Dfid), the Medical Research Council, Wellcome and Department of Health and Social Care (Grant Ref: MR/R006040/1).

The study third year follow up is supported by a grant from Bill and Melinda Gate foundation, through the Innovative Vector Control Consortium.

The London School of Hygiene and Tropical Medicine is the trial sponsor.

Competing interests

The authors declare that they have no competing interests.

For peer review only - http://bmjopen.bmj.com/site/about/guidelines.xhtml

Clinical

To assess the effectiveness of py-PPF-LLINs, py-CFP-LLINs and py-PBO-LLINs compared to s-LLINs:

- On malaria incidence in children from 6 months to 10 years over two years
- On prevalence of moderate and severe anaemia in children under 5 years old
- On malaria infection prevalence in children from 6 months to 14 years after three years post intervention

To evaluate the comparative effectiveness of the 3 Dual-AI LLINs on malaria prevalence and malaria incidence

Entomological

To assess the effectiveness of py-PPF-LLINs, py-CFP-LLINs and py-PBO-LLINs compared to s-LLINs:

- On EIR of malaria vectors collected indoors three years post intervention
- On insecticide resistance mechanisms (target site mutations and metabolic enzyme expression) in *Anopheles gambiae* s.l. and *Anopheles funestus s.l.*
- On other entomological outcomes; 1) indoor and outdoor density, 2) feeding and resting preference, 3) parous rates and 4) ovary development and fecundity (only py-PPF-LLINs) in *Anopheles gambiae* s.l. and *Anopheles funestus s.l.*

To evaluate the comparative effectiveness of the 3 Dual-AI LLINs on EIR

Economical

To assess the incremental budget impact of each of the Dual-AI LLINs relative to s-LLINs and one another

Secondary objectives

338x190mm (96 x 96 DPI)

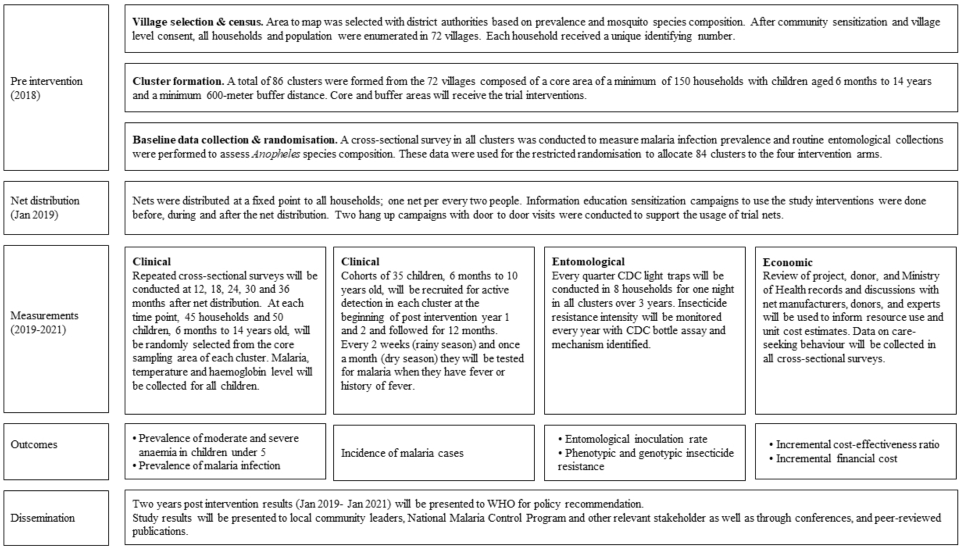

Trial study design

338x190mm (96 x 96 DPI)

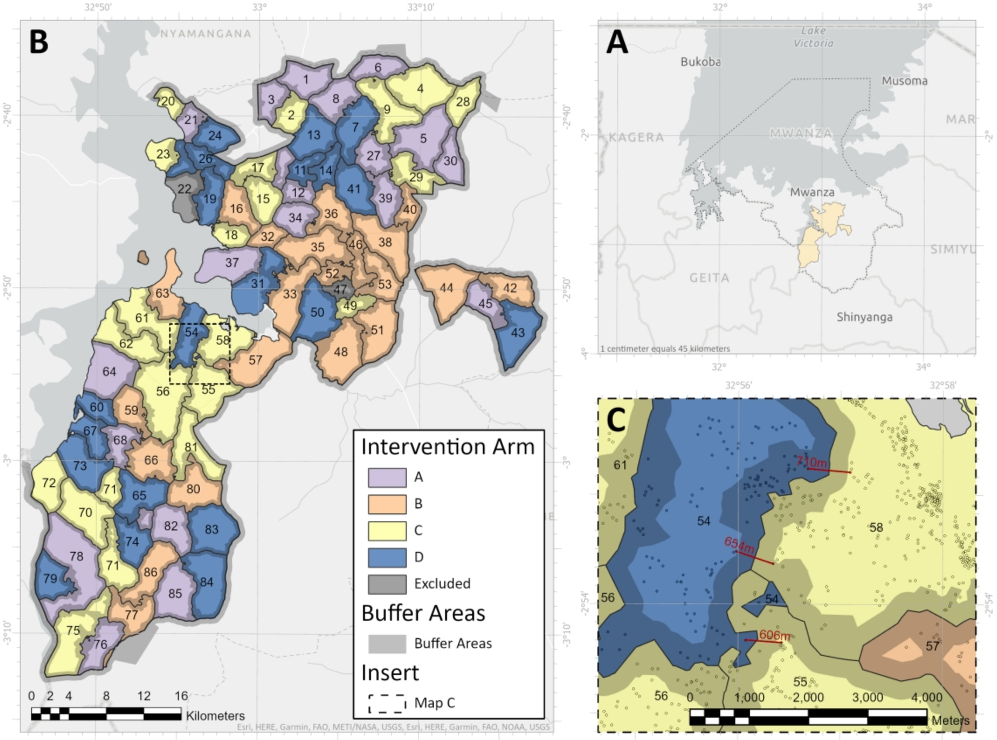

Study area. Map showing Misungwi study area in Mwanza region, North Tanzania (panel A), the 85 study clusters identified with core and buffer area and intervention allocation (panel B). Panel C shows a closer map on the minimum 600 hundred meters area between houses in adjacent clusters.

224x170mm (300 x 300 DPI)

Protocol for a four parallel-arm, single-blind, cluster-randomized trial to assess the effectiveness of three types of dual active ingredient treated nets compared to pyrethroid-only long-lasting insecticidal nets to prevent malaria transmitted by pyrethroid insecticide-resistant vector mosquitoes in Tanzania

Supplementary files

Methods

Ecological niche modelling

Suitability for each *Anopheles* species was determined using an ecological niche modelling approach, based on a maximum entropy (MaxEnt v.3.4.1) algorithm, similar to the approach described by Kulkarni et al. [1]. MaxEnt uses presence-only occurrence data in conjunction with environmental data to predict the potential distribution of a species across a defined landscape. Models were constructed for each major local species (*An. gambiae* s.s., *An. arabiensis* and *An. funestus* s.l.) using occurrence records from pilot mosquito monitoring alongside bioclimatic variables at a 1-km resolution from the WorldClim database (www.worldclim.org), including: temperature seasonality, annual precipitation, precipitation of wettest month, precipitation of driest month, and precipitation seasonality; and elevation from the Shuttle Radar Topography Mission (SRTM). To discriminate between sibling species of the An. gambiae s.l. complex, point locations where only An. gambiae s.s. (i.e. no An. arabiensis) was detected (n=12) were used as occurrence records. Point locations where >50% of the Anopheles mosquitoes were identified as An. funestus s.l. (n=51) were used as occurrence records for this species. Data were randomly partitioned for model evaluation, with 75% of the records used as training data to construct the models and the remaining 25% set aside for testing. The accuracy of each model was determined by performing both a threshold-dependent binomial test of omission and a threshold-independent receiver operating characteristic analysis; models with an area under the curve (AUC) >0.8 were retained, and the model with the highest AUC value for each species was used for subsequent analysis. The mean probability of suitability for each cluster was calculated using zonal statistics in ArcGIS.

Entomological data collection

For peer review only

Routine entomology collection

Cross-sectional entomological surveys will be carried out in 84 study clusters to monitor the indoor mosquito population density. Each cluster will be visited once every quarter; each month, 7 clusters from each study arm will be selected. Indoor mosquito densities will be monitored using Centers for Disease Control and Prevention (CDC) Miniature light traps (John W Hock Company, USA) in 8 randomly selected households in each of the cluster core areas. During the collection night, the owner's bed net in the bed/sleeping place where the CDC light trap will be installed will be substituted with a project standard LLIN, and replaced the following day. For each of the selected houses, a short questionnaire will be administered to collect information about the number of inhabitants, type of house construction materials, presence of animals, where the animals are kept, coverage and usage of study nets and other net types found, and other malaria prevention measures used by household members. Sampled mosquitoes will be identified morphologically following the identification key by Gillies and Coetzee [2]. Physiological status of *Anopheles* mosquitoes (blood-fed, unfed, gravid, semi-gravid) will be recorded. Parity rates will be estimated in a subset of live *Anopheles* mosquitoes through dissection [3]. Maximum 10 *An. gambiae* s.l and 10 *An. funestus* s.l will be randomly picked per household per collection night and preserved for molecular analysis. The mosquito specimens will be screened for *Plasmodium falciparum* circumsporozoite protein (Pf-CSP) by ELISA [4]. The CSP-ELISA positive samples will be re-analysed by heating the ELISA lysates to remove any false positives [5].

All specimens positive for Pf-CSP, as well as a subsample of CSP negative of *An. gambiae* s.l. and *An. funestus* s.l from each cluster per survey round, will be analysed for sibling species identification. PCR TaqMan assays will be used to discriminate members of *An. gambiae* species complex [6], and members of the *An. funestus* group [7]. A subsample of *An. gambiae* s.s. and *An. arabiensis* that are screened for malaria sporozoites infection will be genotyped for the L1014F-*kdr* and L1014S-*kdr* mutations, associated with pyrethroid and DDT resistance, using TaqMan PCR assays, following the protocol by Bass *et al* [8].

Insecticide resistance monitoring

Phenotypic resistance and resistance levels to alpha-cypermethrin, permethrin, and chlorfenapyr, will be characterized at baseline and yearly post-intervention, in all four study arms, using WHO cylinder and modified CDC bottle bioassays [9, 10]. The PBO synergist effect on wild female *An. gambiae* s.l. and *An. funestus* s.l. will be assessed using pre-exposure of PBO in CDC bottles followed by permethrin resistance intensity assays. The effect of PPF on fertility/egg development in adult female *Anopheles*, after 72 hours post-exposure to PPF using CDC bottle bioassays, will be assessed by ovarian dissection under a light microscope [11]. The developmental status of ovarian follicles/eggs will be classified as fertile or infertile and interpreted following the Christopher's stages of egg development [12, 13]. All assays will be performed using wild, indoor resting mosquitoes collected from house walls using

For peer review only

Prokopack and manual aspirators. Mosquitoes will be held for three days, fed on 10% glucose solution, to allow for blood-meal digestion, prior to bioassay testing. Mosquitoes will be morphologically identified as *An. gambiae* s.l. or *An. funestus* s.l. [2], and species complexes will be tested separately [6, 7]. All knockdown/dead mosquitoes at 60 minutes and surviving 72 hours post-exposure to insecticides will be stored individually in RNAlater® and preserved at -20°C for gene expression analysis. Per mosquito, 4-6 legs will be removed to identify individuals to species-level using TaqMan PCR [34-36]; only PCR-confirmed *An. gambiae* s.s. or *An. funestus* s.s., will be pooled separately for further analysis. RNA will be extracted from pools of *An. gambiae* s.s. or *An. funestus* s.s and cDNA synthesized, according to standard procedures. Relative expression of CYPs and other metabolic enzymes, previously identified as being over-expressed in resistant *An. gambiae* s.s. and *An. funestus* s.s. populations in Tanzania [14], will be measured using multiplex TaqMan RT-qPCR assays [15].

Mosquito behaviour

Malaria vector abundance, species composition, feeding and resting behaviours (including biting time, host preference), and contribution to outdoor malaria transmission will be assessed at baseline and after implementation of the interventions to assess changes over time. These mosquito behavioural adaptations will be assessed twice a year during the high mosquito density seasons (April-May and October-November)using CDC light traps indoor, occupied adapted Furvela tent traps outdoors [16] and indoor and outdoor resting collection using Prokopack aspirators. Collections will be done in 2 houses in 40 sentinel clusters (10 clusters per treatment arm) selected for their had high density of mosquitoes. In each household, light traps will be set up inside and one tent installed outside, close to the house. In the house were the mosquito traps will be installed, indoor resting mosquito collections will be conducted on the walls, roofs, and inside the nets. Similarly, outdoor collections of resting mosquitoes will be performed in potential resting sites around the house, such as open resting structures, cow sheds and pit latrines. A sub-sample of An. gambiae s.l. and An. funestus s.l. per each collection method will be tested for the presence of Pf-CSP [4] and identified to sibling species level [6, 7]. All freshly collected blood-fed Anopheles mosquitoes will be screened for the blood meal sources[17] .

The sterilizing effect of py-PPF ITN (Royal Guard®) relative to the standard LLINs on egg development in female wild malaria vectors will be assessed. This study will be conducted once a year in 10 sentinel clusters receiving Royal Guard, and 10 sentinel clusters receiving the standard LLIN. 10 houses per cluster will be visited, and sampled for freshly blood fed mosquitoes resting on the wall or inside the nets. Anopheles will be supplied with 10% glucose solution, monitored for survival and dissected 72 hours after collection to look at the developmental stages of eggs as a proxy of fertility [11]. Gravid alive females will be anesthetized at −20°C for 8–10 min and individually dissected in a drop of distilled water by gently pulling out the last two segments, 7th and 8th sternites of the mosquito abdomen.

For peer review only

References

1. Kulkarni MA, Desrochers RE, Kerr JT: **High resolution niche models of malaria vectors in northern Tanzania: a new capacity to predict malaria risk?** *PLoS One* 2010, **5:**e9396.

2. Gillies MTC, M.: *A supplement to the Anophelinae of Africa south of the Sahara (Afrotropical region).*1987.

3. Detinova T, Gillies M: **Observations on the determination of the age composition and epidemiological importance of populations of Anopheles gambiae Giles and Anopheles funestus Giles in Tanganyika.** *Bulletin of the World Health Organization* 1964, **30:**23.

4. Wirtz RA, Zavala F, Charoenvit Y, Campbell GH, Burkot TR, Schneider I, Esser KM, Beaudoin RL, Andre RG: **Comparative testing of monoclonal antibodies against Plasmodium falciparum sporozoites for ELISA development.** *Bull World Health Organ* 1987, **65:**39-45.

5. Durnez L, Van Bortel W, Denis L, Roelants P, Veracx A, Trung HD, Sochantha T, Coosemans M: **False positive circumsporozoite protein ELISA: a challenge for the estimation of the entomological inoculation rate of malaria and for vector incrimination.** *Malaria Journal* 2011, **10:**195.

6. Bass C, Williamson MS, Field LM: **Development of a multiplex real-time PCR assay for identification of members of the Anopheles gambiae species complex.** *Acta tropica* 2008, **107:**50-53.

7. Vezenegho SB, Bass C, Puinean M, Williamson MS, Field LM, Coetzee M, Koekemoer LL: **Development of multiplex real-time PCR assays for identification of members of the Anopheles funestus species group.** *Malaria Journal* 2009, **8:**1-9.

8. Bass C, Nikou D, Donnelly MJ, Williamson MS, Ranson H, Ball A, Vontas J, Field LM: **Detection of knockdown resistance (kdr) mutations in Anopheles gambiae: a comparison of two new high-throughput assays with existing methods.** *Malaria Journal* 2007, **6:**1-14.

9. Brogdon WG, Chan A: **Guideline for evaluating insecticide resistance in vectors using the CDC bottle bioassay.** Altlanta, USA: Centers for Disease Control and Prevention; 2012.

10. WHO: **Test procedures for insecticide resistance monitoring in malaria vector mosquitoes (Second edition).** Geneva, Switzerland: World Health organization; 2016.

11. Koama B, Namountougou M, Sanou R, Ndo S, Ouattara A, Dabire RK, Malone D, Diabate A: **The sterilizing effect of pyriproxyfen on the malaria vector Anopheles gambiae: physiological impact on ovaries development.** *Malar J* 2015, **14:**101.

12. Christophers SR: **The development of the egg follicles in anophelines.** *Paludism* 1911, **2:**73-88.

13. Koama B, Namountougou M, Sanou R, Ndo S, Ouattara A, Dabiré RK, Malone D, Diabaté A: **The sterilizing effect of pyriproxyfen on the malaria vector Anopheles gambiae: physiological impact on ovaries development.** *Malaria journal* 2015, **14:**101.

For peer review only

14. Matowo J, Kitau J, Kaaya R, Kavishe R, Wright A, Kisinza W, Kleinschmidt I, Mosha F, Rowland M, Protopopoff N: **Trends in the selection of insecticide resistance in Anopheles gambiae s.l. mosquitoes in northwest Tanzania during a community randomized trial of longlasting insecticidal nets and indoor residual spraying.** *Med Vet Entomol* 2015, **29:**51-59.

15. Mavridis K, Wipf N, Medves S, Erquiaga I, Muller P, Vontas J: **Rapid multiplex gene expression assays for monitoring metabolic resistance in the major malaria vector Anopheles gambiae.** *Parasit Vectors* 2019, **12:**9.

16. Charlwood JD, Rowland M, Protopopoff N, Le Clair C: **The Furvela tent-trap Mk 1.1 for the collection of outdoor biting mosquitoes.** *PeerJ* 2017, **5:**e3848.

17. Beier JC, Perkins PV, Wirtz RA, Koros J, Diggs D, Gargan TP, Koech DK: **Bloodmeal identification by direct enzyme-linked immunosorbent assay (ELISA), tested on Anopheles (Diptera: Culicidae) in Kenya.** *Journal of medical entomology* 1988, **25:**9-16.

For peer review only

ANNEXE: Consent forms (English)

1 Consent forms: Household and prevalence survey

For peer review only

HOUSEHOLD AND MALARIA PREVALENCE SURVEY INFORMED CONSENT AGREEMENT

Introduction

Good morning. My name is ____. I work with PAMVERC Malaria Prevention Trial Missungwi. We work together with the Missungwi District Health office, the National Institute for Medical Research, the Kilimanjaro Christian Medical College, London School of Hygiene and Tropical Medicine. I am here to ask you some questions and do some simple tests to learn how well malaria prevention and treatment are working in Missungwi. To do this, we are gathering information by visiting a number of households in your community.

Purpose of the survey

To see if the malaria program works, we would like to ask you some general questions about your household, bed-net possession, and use. We want to see how common malaria is among people in your community by testing for parasites in their blood. Your responses to our questions and the results of our studies will help us learn how best to further improve malaria control in your community and in the country.

Procedures

- If you agree to take part, we will ask you a number of questions about your family and household about bed nets used. Some people will be selected to be tested for malaria. I will ask all selected people to go to see a PAMVERC-employed nurse on the ____________ (give the date of the consultation) in _____________ (give the place). The nurse will take several small drops of blood from each selected person. The whole process should take about 30 minutes.
- The nurse will take a small amount of blood from the finger using a small needle. One drop of blood will be used to test for a rapid malaria diagnostic test, and other drop to prepare the blood slide. This blood slide will be analysed in a laboratory in KCMC and may need to be kept for further analysis after the survey. A drop of blood will also be used to test for anaemia. The identity of the person will not be connected to these samples. We will also test whether the person currently has fever.
- The results from the malaria rapid diagnostic test will be given the same day. If the person has malaria or fever, he will be provided with free drugs by the PAMVERC clinician. In case the person does not get better, you are requested to go to the nearest health facility immediately to receive alternative treatment according to the Ministry of Health policies. If we diagnose any person as having severe malaria or other diseases you will be immediately referred to nearby health facilities.
- Net inspection: If you agree to take part in this survey, we will ask you additional questions about the net you have (washing, type of sleeping bed and repair) and would like also to see and inspect two nets in your house to assess the quality. This will help us determine for how long these nets can sustain different field condition. Therefore, enable us devise alternative measures for improving and strengthening nets to meet community needs. I will not damage the net, and after the interview and will return it after the inspection.

Risks and Benefits

The tested person will feel pain for a few seconds when we take the blood from his/her finger. If the test shows that your child has malaria, or fever at the time of the survey, they will receive free treatment that the Ministry of Health recommends. These drugs are proven to be safe and effective, but any drugs can cause side effects in a small number of patients. The nurse will discuss if treatment is needed.

Voluntariness and confidentiality

It is entirely your choice to take part in or not take part in this survey as I have just described it. If you do agree to take part, your individual answers to all questions and the test results will be kept private and not revealed to anyone. If you agree to take part, you can also decide not to answer any of the questions that you do not want to, and you can refuse the blood tests.

Costs and compensation for participating in the study

You will not be asked to pay anything for you to participate in this study. The study will not reimburse you with any payment for taking part in the study.

For peer review only - http://bmjopen.bmj.com/site/about/guidelines.xhtml

Consent for long-term sample storage for future studies

We are also asking people who join this study if they will let the researchers' use their blood sample for future studies. These future studies may help find new ways to prevent malaria or other diseases. If you agree, we will store your blood in the laboratory with a unique number and not with your name. Your sample will be stored for up to 25 years. We may share your test results with researchers at other organizations but we will not give them your name, address, or any information that could identify you. After the study has ended, we will remove any means to link the sample to you, and we will not be able to find your sample. If you do not wish to have your blood stored for future tests, you may still participate in our study.

The London School of Hygiene and Tropical Medicine is the Sponsor and hold insurance policies which apply to this study

Thank you very much for your time. Would you like to take part in this survey?

HOUSEHOLD COPY – Household and child – Date: ___/___/___

Consent

- The study has been explained to me, I have been given the opportunity to ask questions concerning this study. Any such questions have been answered to my full satisfaction. I understand participation is voluntary and I may revoke this consent at any time without penalty or loss of benefits, if any.
- I agree for me and my child/children to take part.
- I agree that the data generated from this study can be used in the future for other malaria related research. Yes |___| No |___|
  I agree that the dried blood samples stored can be used in the future for other malaria related research. Yes |___| No |___|

Name of participant.. Signature/Thumb print ....

Relationship to the children...

Name of the witness..Signature..

Name of interviewer..Signature..

If you have any questions or clarification pertaining to this survey please feel free to ask the field workers and nurse or you may contact Mr Eliud Lukole, PAMVERC, 0766240101; Dr Jackline Mosha, NIMR Mwnza, 0754404140; Dr Alphaxard Manjurano, 0756026661.

If you have any questions about your rights as a study patient, or if you think your child has been injured because of this study, please contact the Chairman of the National Health Research Ethics Committee (NatHREC) on 0222 121 400/390

For peer review only - http://bmjopen.bmj.com/site/about/guidelines.xhtml

PROJECT COPY – Household and child

Date: ___/____/_____ Cluster Number: ___ ___ Household Number: ___ ___ ___

MALARIA PREVALENCE SURVEY INFORMED CONSENT AGREEMENT FOR THE PARENT / GUARDIAN OF CHILDREN IN THE TRIAL

Consent

- The study has been explained to me, I have been given the opportunity to ask questions concerning this study. Any such questions have been answered to my full satisfaction. I understand participation is voluntary and I may revoke this consent at any time without penalty or loss of benefits, if any.
- I agree for me and my child/children to take part.
- I agree that the data generated from this study can be used in the future for other malaria related research. Yes |___| No |___|
  I agree that the dried blood samples stored can be used in the future for other malaria related research. Yes |___| No |___|

Name of participant.. Signature/Thumb print ....

Relationship to the children...

Name of the witness...Signature..

Name of interviewer...Signature..

If you have any questions or clarification pertaining to this survey please feel free to ask the field workers and nurse or you may contact Mr Eliud Lukole, PAMVERC, 0766240101; Dr Jackline Mosha, NIMR Mwnza, 0754404140 and Dr Alphaxard Manjurano, 0756026661.

If you have any questions about your rights as a study patient, or if you think your child has been injured because of this study, please contact the Chairman of the National Health Research Ethics Committee (NatHREC) on 0222 121 400/390

For peer review only

2 Consent forms: Children cohort follow up

For peer review only

CHILDREN COHORT FOLLOW UP CONSENT FORM

Introduction

Good morning. My name is ____. I work with PAMVERC Malaria Prevention Trial Missungwi. We work together with the Missungwi District Health office, the National Institute for Medical Research, the Kilimanjaro Christian Medical College, London School of Hygiene and Tropical Medicine. I am here to ask you some questions and do some simple tests to learn how well malaria prevention is working in Missungwi.

Purpose of the survey

To see if the mosquito net distributed works. We want to know if children in your community have malaria by taking their temperature and testing for parasites in their blood if they feel unwell. Your responses to our questions and the results of our studies will help us learn how best to further improve malaria control in your community and in the country.

Procedures

If you agree to take part, we will select at random one children from 6 months to 10 years from your household to attend every month the PAMVERC mobile clinic located in your hamlet or a nearby hamlet. We will ask you some questions about the child, including if he had fever, if he is sick and received a treatment, bed-net use and any side effect from the use of bed net. We will take his temperature. If your child has symptoms of malaria infection the nurse will take several small drops of blood to diagnose malaria. The whole process should take about 30 minutes. The selected child will be followed for one year or until he/she reach 10 years old.

To diagnose malaria, the nurse will take a small amount of blood from the finger using a small needle. One drop of blood will be used to test for a rapid malaria diagnostic test. The identity of the child will not be connected to these samples.

The results from the malaria rapid diagnostic test will be given the same day. If the person has malaria or fever, he will be provided with free drugs by the PAMVERC clinician. In case the person does not get better, you are requested to go to the nearest health facility immediately to receive alternative treatment according to the Ministry of Health policies. If we diagnose any person with severe malaria or other diseases, you will be immediately referred to nearby health facilities.

Risks and Benefits

The tested person will feel pain for a few seconds when we take the blood from his/her finger. If the test shows that your child has malaria, or fever at the time of the survey, they will receive free treatment that the Ministry of Health recommends. These drugs are proven to be safe and effective, but any drugs can cause side effects in a small number of patients. The nurse will discuss if a treatment is needed.

Voluntariness and confidentiality

It is entirely your choice to take part or not in this survey as I have just described it. If you do agree to take part, your individual answers to all questions and the test results will be kept private and not revealed to anyone. If you agree to take part, you can decide not to answer some of the questions, and you can also refuse the blood tests.

Costs and compensation for being in the study

You will not be asked to pay anything for you to participate in this study. You will receive reimbursement for your transport to come to the mobile clinic and go back home. The total amount will be on average 2,000Tsh for each visit.

For peer review only - http://bmjopen.bmj.com/site/about/guidelines.xhtml

For peer review only

Consent for long-term sample storage for future studies

We are also asking people who join this study if they will let the researchers' use their blood sample for future studies. These future studies may help find new ways to prevent malaria or other diseases. If you agree, we will store your blood in the laboratory with a unique number and not with your name. Your sample will be stored for up to 25 years. We may share your test results with researchers at other organizations but we will not give them your name, address, or any information that could identify you. After the study has ended, we will remove any means to link the sample to you, and we will not be able to find your sample. If you do not wish to have your blood stored for future tests, you may still participate in our study.

The London School of Hygiene and Tropical Medicine is the Sponsor and hold insurance policies which apply to this study

Thank you very much for your time. Would you like to take part in this survey?

HOUSEHOLD COPY – Household and child – Date: ___/___/___

CONSENT

- The study has been explained to me, I have been given the opportunity to ask questions concerning this study. Any such questions have been answered to my full satisfaction. I understand participation is voluntary and I may revoke this consent at any time without penalty or loss of benefits, if any.
- I agree for me and my child/children to take part.
- I agree that the data generated from this study can be used in the future for other malaria related research. Yes |___| No |___|
- I agree that the dried blood samples stored can be used in the future for other malaria related research. Yes |___| No |___|

Name of guardian/parent.. Signature/Thumb print ....

Name of the child selected.......................................Relationship to the chil...

Name of the witness..Signature...

Name of interviewer...Signature...

If you have any questions or clarification pertaining to this survey please feel free to ask the field workers and nurse or you may contact Mr Eliud Lukole, PAMVERC, 0766240101; Dr Jackline Mosha, NIMR Mwnza, 0754404140; Dr Alphaxard Manjurano, 0756026661;

If you have any questions about your rights as a study patient, or if you think your child has been injured because of this study, please contact the Chairman of the National Health Research Ethics Committee (NatHREC) on 0222 121 400/390]

For peer review only - http://bmjopen.bmj.com/site/about/guidelines.xhtml

PROJECT COPY – Household and child

Date: ___/____/_____ Cluster Number: ___ ___ Household Number: ___ ___ ___

CHILDREN COHORT FOLLOW UP CONSENT FORM

CONSENT

- The study has been explained to me, I have been given the opportunity to ask questions concerning this study. Any such questions have been answered to my full satisfaction. I understand participation is voluntary and I may revoke this consent at any time without penalty or loss of benefits, if any.
- I agree for me and my child/children to take part.
- I agree that the data generated from this study can be used in the future for other malaria related research. Yes |___| No |___|

I agree that the dried blood samples stored can be used in the future for other malaria related research. Yes |___| No |___|

Name of guardian/parent.. Signature/Thumb print ....

Name of the child selected.......................................Relationship to the child...

Name of the witness...Signature...

Name of interviewer...Signature...

If you have any questions or clarification pertaining to this survey please feel free to ask the field workers and nurse or you may contact Study staff; Mr Eliud Lukole, PAMVERC, 0766240101; Dr Jackline Mosha, NIMR Mwnza, 0754404140; Dr Alphaxard Manjurano, 0756026661;

If you have any questions about your rights as a study patient, or if you think your child has been injured because of this study, please contact the Chairman of the National Health Research Ethics Committee (NatHREC) on 0222 121 400/390]

For peer review only - http://bmjopen.bmj.com/site/about/guidelines.xhtml

3 Consent forms: Mosquito trapping

For peer review only

For peer review only - http://bmjopen.bmj.com/site/about/guidelines.xhtml

Mosquito trapping informed consent agreement: V1.0 (19/01/2018)

Introduction

Good morning. My name is __________. I work with PAMVERC Malaria Prevention Trial in Missungwi. We work together with the Missungwi District Health office, the National Institute for Medical Research, the Kilimanjaro Christian Medical College, London School of Hygiene and Tropical Medicine.

Purpose of the survey

We would like to include you in a study to find out if different new nets products as one of the control intervention can reduce the transmission of malaria in your communities. Malaria is transmitted by mosquitoes that carry the malaria parasite. The control interventions reduce the number of infected mosquitoes. We want to find out whether the LLINs reduce the number of mosquitoes flying into your house. It will provide information on which new LLINs works best to reduce mosquito numbers and malaria.

Procedure for light trap catching

If you agree to participate, we will carry out the following activities:

1. Collection of mosquitoes using a special light trap in your bedroom for one night. The trap will collect mosquitoes coming indoors and we will collect the trap early the following morning. The trap light will be turned on in the early evening and will be on through the night. You and others in the room will sleep under a bed net which we will provide for you, and for the others *if necessary*, on the night we collect mosquitoes.
2. You will be asked to complete a short questionnaire. We will ask a few questions about your house and any mosquito control you may have used. For this process you will be identified by a study code, not by your name, so that the views you express and answers you provide will remain completely anonymous.

Procedure for tent trap collection

If you agree to participate, we will carry out the following activities:

1. Collection of mosquitoes using a special trap that will be set up outside your house. The trap will collect outdoors mosquitoes. We will ask you permission to install a tent nearby your houses and allow us to sleep under.
2. You will be asked to complete a short questionnaire. We will ask a few questions about your house and any mosquito control you may have used. For this process you will be identified by a study code, not by your name, so that the views you express and answers you provide will remain completely anonymous.

Procedure for collection of resting mosquitoes

1. We will collect mosquitoes resting on your wall and inside your net early on the morning around 6-7 am.
2. For this process your house will be identified by a study code, so that the result cannot be related to you

Voluntariness and confidentiality

It is entirely your choice to take part in or not take part in this survey as I have just described it. If you agree to take part, you can also decide not to answer any of the questions that you do not want to. Your individual information will be kept private.

Risks and Benefits:

For peer review only - http://bmjopen.bmj.com/site/about/guidelines.xhtml

We can see no risk in taking part in this study. If you are not sleeping under a long-lasting Net you will receive one to sleep under the night the trap is running. The traps may reduce the number of mosquitoes in your house. The results of the study will help us learn how best malaria can be controlled.

Costs and compensation for participating in the study

You will not be asked to pay anything for you to participate in this study. The study will not reimburse you with any payment for taking part in the study.

The London School of Hygiene and Tropical Medicine is the Sponsor and hold insurance policies which apply to this study

Thank you very much for your time. Would you like to take part in this survey?

HOUSEHOLD COPY Date: _____/______/________

Mosquito trapping Informed Consent agreement

Consent section

- The study has been explained to me, I have been given the opportunity to ask questions concerning this study. Any such questions have been answered to my full satisfaction. I understand participation is voluntary and I may revoke this consent at any time without penalty or loss of benefits, if any.
- I agree to take part to the survey
- I also agree that the data generated from this study and the dried blood samples stored can be used in the future for other malaria related research.

Name of guardian/parent.. Signature/Thumb print ....

Name of the witness..Signature...

Name of interviewer...Signature...

If you have any questions or clarification pertaining to this survey please feel free to ask the field workers or you may contact Mr Eliud Lukole, PAMVERC, 0766240101; Dr Jackline Mosha, NIMR Mwnza, 0754404140; Dr Alphaxard Manjurano, 0756026661.

If you have any questions about your rights as a study patient, or if you think your child has been injured because of this study, please contact the Chairman of the National Health Research Ethics Committee (NatHREC) on 0222 121 400/390

For peer review only - http://bmjopen.bmj.com/site/about/guidelines.xhtml

PROJECT COPY Date _____/______/______

Cluster Number: ___ ___Household Number: ___ ___ ___ Round: __ __

Mosquito trapping Informed Consent agreement

Consent section

The study has been explained to me, I have been given the opportunity to ask questions concerning this study. Any such questions have been answered to my full satisfaction. I understand participation is voluntary and I may revoke this consent at any time without penalty or loss of benefits, if any.

I agree to take part.

Name of guardian/parent.. Signature/Thumb print ....

Name of the witness...Signature...

Name of interviewer..Signature..

If you have any questions or clarification pertaining to this survey please feel free to ask the field workers or you may contact Mr Eliud Lukole, PAMVERC, 0766240101; Dr Jackline Mosha, NIMR Mwnza, 0754404140; Dr Alphaxard Manjurano, 0756026661

If you have any questions about your rights as a study patient, or if you think your child has been injured because of this study, please contact the Chairman of the National Health Research Ethics Committee (NatHREC) on 0222 121 400/390

For peer review only

For peer review only

For peer review only - http://bmjopen.bmj.com/site/about/guidelines.xhtml

STANDARD PROTOCOL ITEMS: RECOMMENDATIONS FOR INTERVENTIONAL TRIALS

SPIRIT 2013 Checklist: Recommended items to address in a clinical trial protocol and related documents*

Section/item	Item No	Description	Addressed on page number
**Administrative information**			
Title	1	Descriptive title identifying the study design, population, interventions, and, if applicable, trial acronym	___1
Trial registration	2a	Trial identifier and registry name. If not yet registered, name of intended registry	___1
	2b	All items from the World Health Organization Trial Registration Data Set	___NA
Protocol version	3	Date and version identifier	in supplementary file (protocol)
Funding	4	Sources and types of financial, material, and other support	___36
Roles and responsibilities	5a	Names, affiliations, and roles of protocol contributors	___1
	5b	Name and contact information for the trial sponsor	___36
	5c	Role of study sponsor and funders, if any, in study design; collection, management, analysis, and interpretation of data; writing of the report; and the decision to submit the report for publication, including whether they will have ultimate authority over any of these activities	in supplementary file (protocol)
	5d	Composition, roles, and responsibilities of the coordinating centre, steering committee, endpoint adjudication committee, data management team, and other individuals or groups overseeing the trial, if applicable (see Item 21a for data monitoring committee)	in supplementary file (protocol)

For peer review only

Introduction

Background and rationale	6a	Description of research question and justification for undertaking the trial, including summary of relevant studies (published and unpublished) examining benefits and harms for each intervention	__5 - 8
	6b	Explanation for choice of comparators	__12 - 13
Objectives	7	Specific objectives or hypotheses	__8
Trial design	8	Description of trial design including type of trial (eg, parallel group, crossover, factorial, single group), allocation ratio, and framework (eg, superiority, equivalence, noninferiority, exploratory)	__10

Methods: Participants, interventions, and outcomes

Study setting	9	Description of study settings (eg, community clinic, academic hospital) and list of countries where data will be collected. Reference to where list of study sites can be obtained	__8 - 9
Eligibility criteria	10	Inclusion and exclusion criteria for participants. If applicable, eligibility criteria for study centres and individuals who will perform the interventions (eg, surgeons, psychotherapists)	__15
Interventions	11a	Interventions for each group with sufficient detail to allow replication, including how and when they will be administered	__12 - 13
	11b	Criteria for discontinuing or modifying allocated interventions for a given trial participant (eg, drug dose change in response to harms, participant request, or improving/worsening disease)	__NA
	11c	Strategies to improve adherence to intervention protocols, and any procedures for monitoring adherence (eg, drug tablet return, laboratory tests)	__12 - 13
	11d	Relevant concomitant care and interventions that are permitted or prohibited during the trial	__17
Outcomes	12	Primary, secondary, and other outcomes, including the specific measurement variable (eg, systolic blood pressure), analysis metric (eg, change from baseline, final value, time to event), method of aggregation (eg, median, proportion), and time point for each outcome. Explanation of the clinical relevance of chosen efficacy and harm outcomes is strongly recommended	___14
Participant timeline	13	Time schedule of enrolment, interventions (including any run-ins and washouts), assessments, and visits for participants. A schematic diagram is highly recommended (see Figure)	in supplementary file (protocol)

For peer review only - http://bmjopen.bmj.com/site/about/guidelines.xhtml

Sample size	14	Estimated number of participants needed to achieve study objectives and how it was determined, including clinical and statistical assumptions supporting any sample size calculations	___21 - 22
Recruitment	15	Strategies for achieving adequate participant enrolment to reach target sample size	___21 - 22
**Methods: Assignment of interventions (for controlled trials)**			
Allocation:			
Sequence generation	16a	Method of generating the allocation sequence (eg, computer-generated random numbers), and list of any factors for stratification. To reduce predictability of a random sequence, details of any planned restriction (eg, blocking) should be provided in a separate document that is unavailable to those who enrol participants or assign interventions	___11 - 12
Allocation concealment mechanism	16b	Mechanism of implementing the allocation sequence (eg, central telephone; sequentially numbered, opaque, sealed envelopes), describing any steps to conceal the sequence until interventions are assigned	___12
Implementation	16c	Who will generate the allocation sequence, who will enrol participants, and who will assign participants to interventions	___12
Blinding (masking)	17a	Who will be blinded after assignment to interventions (eg, trial participants, care providers, outcome assessors, data analysts), and how	___10
	17b	If blinded, circumstances under which unblinding is permissible, and procedure for revealing a participant's allocated intervention during the trial	___10
**Methods: Data collection, management, and analysis**			
Data collection methods	18a	Plans for assessment and collection of outcome, baseline, and other trial data, including any related processes to promote data quality (eg, duplicate measurements, training of assessors) and a description of study instruments (eg, questionnaires, laboratory tests) along with their reliability and validity, if known. Reference to where data collection forms can be found, if not in the protocol	___15 - 20
	18b	Plans to promote participant retention and complete follow-up, including list of any outcome data to be collected for participants who discontinue or deviate from intervention protocols	___15 - 20

For peer review only - http://bmjopen.bmj.com/site/about/guidelines.xhtml

Data management	19	Plans for data entry, coding, security, and storage, including any related processes to promote data quality (eg, double data entry; range checks for data values). Reference to where details of data management procedures can be found, if not in the protocol	___22 - 23
Statistical methods	20a	Statistical methods for analysing primary and secondary outcomes. Reference to where other details of the statistical analysis plan can be found, if not in the protocol	___23 - 27
	20b	Methods for any additional analyses (eg, subgroup and adjusted analyses)	___23 - 24
	20c	Definition of analysis population relating to protocol non-adherence (eg, as randomised analysis), and any statistical methods to handle missing data (eg, multiple imputation)	in supplementary file (protocol)
**Methods: Monitoring**			
Data monitoring	21a	Composition of data monitoring committee (DMC); summary of its role and reporting structure; statement of whether it is independent from the sponsor and competing interests; and reference to where further details about its charter can be found, if not in the protocol. Alternatively, an explanation of why a DMC is not needed	in supplementary file (protocol)
	21b	Description of any interim analyses and stopping guidelines, including who will have access to these interim results and make the final decision to terminate the trial	in supplementary file (protocol)
Harms	22	Plans for collecting, assessing, reporting, and managing solicited and spontaneously reported adverse events and other unintended effects of trial interventions or trial conduct	in supplementary file (protocol)
Auditing	23	Frequency and procedures for auditing trial conduct, if any, and whether the process will be independent from investigators and the sponsor	in supplementary file (protocol)
**Ethics and dissemination**			
Research ethics approval	24	Plans for seeking research ethics committee/institutional review board (REC/IRB) approval	__35
Protocol amendments	25	Plans for communicating important protocol modifications (eg, changes to eligibility criteria, outcomes, analyses) to relevant parties (eg, investigators, REC/IRBs, trial participants, trial registries, journals, regulators)	in supplementary file (protocol)

For peer review only - http://bmjopen.bmj.com/site/about/guidelines.xhtml

Consent or assent	26a	Who will obtain informed consent or assent from potential trial participants or authorised surrogates, and how (see Item 32)	in supplementary file (protocol)
	26b	Additional consent provisions for collection and use of participant data and biological specimens in ancillary studies, if applicable	in supplementary file (protocol)
Confidentiality	27	How personal information about potential and enrolled participants will be collected, shared, and maintained in order to protect confidentiality before, during, and after the trial	___22 - 23
Declaration of interests	28	Financial and other competing interests for principal investigators for the overall trial and each study site	___35
Access to data	29	Statement of who will have access to the final trial dataset, and disclosure of contractual agreements that limit such access for investigators	in supplementary file (protocol)
Ancillary and post-trial care	30	Provisions, if any, for ancillary and post-trial care, and for compensation to those who suffer harm from trial participation	in supplementary file (protocol)
Dissemination policy	31a	Plans for investigators and sponsor to communicate trial results to participants, healthcare professionals, the public, and other relevant groups (eg, via publication, reporting in results databases, or other data sharing arrangements), including any publication restrictions	__3 & 28
	31b	Authorship eligibility guidelines and any intended use of professional writers	__35
	31c	Plans, if any, for granting public access to the full protocol, participant-level dataset, and statistical code	in supplementary file (protocol)
**Appendices**			
Informed consent materials	32	Model consent form and other related documentation given to participants and authorised surrogates	in supplementary file (protocol)
Biological specimens	33	Plans for collection, laboratory evaluation, and storage of biological specimens for genetic or molecular analysis in the current trial and for future use in ancillary studies, if applicable	in supplementary file (protocol)

*It is strongly recommended that this checklist be read in conjunction with the SPIRIT 2013 Explanation & Elaboration for important clarification on the items. Amendments to the protocol should be tracked and dated. The SPIRIT checklist is copyrighted by the SPIRIT Group under the Creative Commons "Attribution-NonCommercial-NoDerivs 3.0 Unported" license.

For peer review only - http://bmjopen.bmj.com/site/about/guidelines.xhtml
