# Supplementary material for: Protocol for a four parallel-arm, single-blind, cluster-randomised trial to assess the effectiveness of three types of dual active ingredient treated nets compared to pyrethroid-only long-lasting insecticidal nets to prevent malaria transmitted by pyrethroid insecticide-resistant vector mosquitoes in Tanzania
Source: BMJ Open. 2021 Mar 8;11(3):e046664. doi: 10.1136/bmjopen-2020-046664 (PMC7942254; doi:10.1136/bmjopen-2020-046664)
Supplement: Reviewer comments [file bmjopen-2020-046664.reviewer_comments.pdf]

PEER REVIEW HISTORY

BMJ Open publishes all reviews undertaken for accepted manuscripts. Reviewers are asked to complete a checklist review form (**http://bmjopen.bmj.com/site/about/resources/checklist.pdf**) and are provided with free text boxes to elaborate on their assessment. These free text comments are reproduced below.

ARTICLE DETAILS

**TITLE (PROVISIONAL)**	Protocol for a four parallel-arm, single-blind, cluster-randomized trial to assess the effectiveness of three types of dual active ingredient treated nets compared to pyrethroid-only long-lasting insecticidal nets to prevent malaria transmitted by pyrethroid insecticide-resistant vector mosquitoes in Tanzania
**AUTHORS**	Mosha, Jacklin; Kulkarni, Manisha; Messenger, Louisa; Rowland, Mark; Matowo, Nancy; Pitt, Catherine; Lukole, Eliud; Taljaard, Monica; Thickstun, Charles; Manjurano, Alphaxard; Mosha, Franklin; Kleinschmidt, Immo; Protopopoff, Natacha

This article was not externally reviewed at BMJ Open. Protocols that have been independently assessed prior to submission to BMJ Open are usually fast-tracked to publication on the grounds that further substantial changes will not be possible. This independent assessment will usually be external, independent review for both a substantial grant award from a non-commercial or government funder and ethics approval.
